# Quantifying connectivity between local *Plasmodium falciparum* malaria parasite populations using identity by descent

**DOI:** 10.1371/journal.pgen.1007065

**Published:** 2017-10-27

**Authors:** Aimee R. Taylor, Stephen F. Schaffner, Gustavo C. Cerqueira, Standwell C. Nkhoma, Timothy J. C. Anderson, Kanlaya Sriprawat, Aung Pyae Phyo, François Nosten, Daniel E. Neafsey, Caroline O. Buckee

**Affiliations:** 1 Center for Communicable Disease Dynamics, Department of Epidemiology, Harvard T.H. Chan School of Public Health, Boston, Massachusetts, United States of America; 2 Infectious Disease and Microbiome Program, Broad Institute, Cambridge, Massachusetts, United States of America; 3 Department of Genetics, Texas Biomedical Research Institute, San Antonio, Texas, United States of America; 4 Shoklo Malaria Research Unit, Mahidol-Oxford Tropical Medicine Research Unit, Faculty of Tropical Medicine, Mahidol University, Mae Sot, Thailand; 5 Centre for Tropical Medicine and Global Health, Nuffield Department of Medicine Research building, University of Oxford, Old Road campus, Oxford, United Kingdom; 6 Department of Immunology and Infectious Disease, Harvard T.H. Chan School of Public Health, Boston, Massachusetts, United States of America; Imperial College London, UNITED KINGDOM

## Abstract

With the rapidly increasing abundance and accessibility of genomic data, there is a growing interest in using population genetic approaches to characterize fine-scale dispersal of organisms, providing insight into biological processes across a broad range of fields including ecology, evolution and epidemiology. For sexually recombining haploid organisms such as the human malaria parasite *P*. *falciparum*, however, there have been no systematic assessments of the type of data and methods required to resolve fine scale connectivity. This analytical gap hinders the use of genomics for understanding local transmission patterns, a crucial goal for policy makers charged with eliminating this important human pathogen. Here we use data collected from four clinics with a catchment area spanning approximately 120 km of the Thai-Myanmar border to compare the ability of divergence (*F*_*ST*_) and relatedness based on identity by descent (IBD) to resolve spatial connectivity between malaria parasites collected from proximal clinics. We found no relationship between inter-clinic distance and *F*_*ST*_, likely due to sampling of highly related parasites within clinics, but a significant decline in IBD-based relatedness with increasing inter-clinic distance. This association was contingent upon the data set type and size. We estimated that approximately 147 single-infection whole genome sequenced parasite samples or 222 single-infection parasite samples genotyped at 93 single nucleotide polymorphisms (SNPs) were sufficient to recover a robust spatial trend estimate at this scale. In summary, surveillance efforts cannot rely on classical measures of genetic divergence to measure *P*. *falciparum* transmission on a local scale. Given adequate sampling, IBD-based relatedness provides a useful alternative, and robust trends can be obtained from parasite samples genotyped at approximately 100 SNPs.

## Author summary

The spatiotemporal dispersal of organisms can inform efforts to conserve endangered species, to contain the spread of drug resistance, and to eliminate disease. As genomic data become increasingly more affordable and accessible via public depositories, the demand for methods capable of extracting fine-scale population structure from genomic data grows. However, to the best of our knowledge, there are no guidelines regarding the type of data and methods required to resolve local spatial trends over sexually recombining haploid organisms, such as the malaria parasite. The approach we present here compares relatedness based on identity by descent, which accounts for recombination while distinguishing genetic identity due to inheritance from genetic identity due to chance, to a classic population genetic measure of divergence, using data from sexually recombining malaria parasites. Using identity by descent, we uncover a significant decrease in highly related malaria parasites collected from proximal clinics on the Thai-Myanmar border, a region where human mobility is high. Our results demonstrate the power of analyses based on identity by descent to detect recent and local trends. Similar analyses could be used to inform the molecular epidemiology of other sexually recombining organisms.

## Introduction

Molecular tools show great promise for helping us understand and contain the spatial spread of pathogens, and the application of population genetic approaches to monitoring and controlling infectious diseases is becoming routine. Routes and volumes of non-sexually recombining pathogens, such as the influenza and Ebola viruses, can be tracked using genomic surveillance [1], enabling time-calibrated phylogenies [2], which can be spatially projected [3], and used to jointly estimate transmission chains [4–7]. For sexually recombining pathogens such as the human malaria parasite *Plasmodium falciparum*, however, these methods are not readily applicable, especially on local spatial scales relevant for control and elimination strategies [8]. Furthermore, *P*. *falciparum* evolves more slowly than viral pathogens, and non-sampled asymptomatic infections, complex within-host dynamics, and extensive within-host diversity (multiple-genotype infections) obscure inference [5,7,9]. As a result, despite increasing efforts to collect genomic data for epidemiological surveillance of malaria on local spatial scales, methods for making sense of them, and guidelines for study design, are lacking.

On large or continental scales, or where recombination is limited, *P*. *falciparum* genetic data have been usefully employed to identify spatial relationships between parasite populations using standard approaches [10]. For example, microsatellite analyses have been used to infer the origins of drug resistant genotypes [11–13] or outbreaks [14], to monitor population dynamics [15,16] and to explore population structure in West Africa [17]; single nucleotide polymorphisms (SNPs) in non-recombining mitochondrion and apicoplast genomes have been used to infer the evolutionary trajectory of the parasite [18]; and whole genome data have been used to interrogate population structure across continents and within Southeast Asia, including Cambodia, the epicenter of drug resistant malaria [19–22]. Explicitly spatial methods applied to these data include tests and scans of spatial autocorrelation [23,24], which are typically suited to highly resolved geo-referenced data. Non-spatial methods include principal component and phylogenetic analyses, as well as many model-based Bayesian methods, including STRUCTURE [25], ChomoPainter and fineSTRUCTURE [26].

Measuring connectivity among proximal populations of *P*. *falciparum* is more challenging, however [27,28]. Classical measures include Wright’s fixation index (*F*_*ST*_) [29,30], a measure of divergence between population pairs, which has been used to recover large-scale population structure in malaria [17,21], but has been shown to be less reliable at smaller spatial scales [20]. More recent studies have investigated relatedness using identity by descent (IBD) and identity by state (IBS), with some promise for smaller spatial scales. Henden and colleagues, for example, constructed networks of related parasites within and across countries using IBD inferred under a probabilistic model that accounts for recombination [31]. On a micro-geographic scale, Omedo and colleagues reported trends in relatedness using IBS [32], which approximates IBD [33], and has been used elsewhere to infer relatedness between malaria parasites [34]. These studies emphasize the need for tools on local scales that can account for transmission between local hotspots, particularly in areas considering or implementing elimination programs, and suggest that IBD-based measures are promising approaches.

IBD is a fundamental concept in population genetics, relating ancestry to variability due to recombination [35]. *F*_*ST*_ can also be interpreted as a measure of IBD stemming from remote inbreeding [36], but unlike IBD, *F*_*ST*_ relies on allelic variation providing a traceable history of co-ancestry. Since recombination works on shorter times scales than mutation and genetic drift, estimates of IBD provide insight into more recent demographic events than *F*_*ST*_ [35], and IBD-based analyses have been used extensively in human genetics (e.g. to impute genotypes, to map disease loci, and to infer demographic histories [35,37]). Increasingly, it is thought that much of the useful signal in the malaria genome lies in the pattern of recombination, rather than variation at any one locus, and IBD is gaining popularity in malaria research and policy (e.g. to monitor disease transmission [38], relatedness within multiple-genotype infections [39], to aid surveillance of antimalarial resistance [40], and to detect signals of selection [31]).

**Fig 1:**
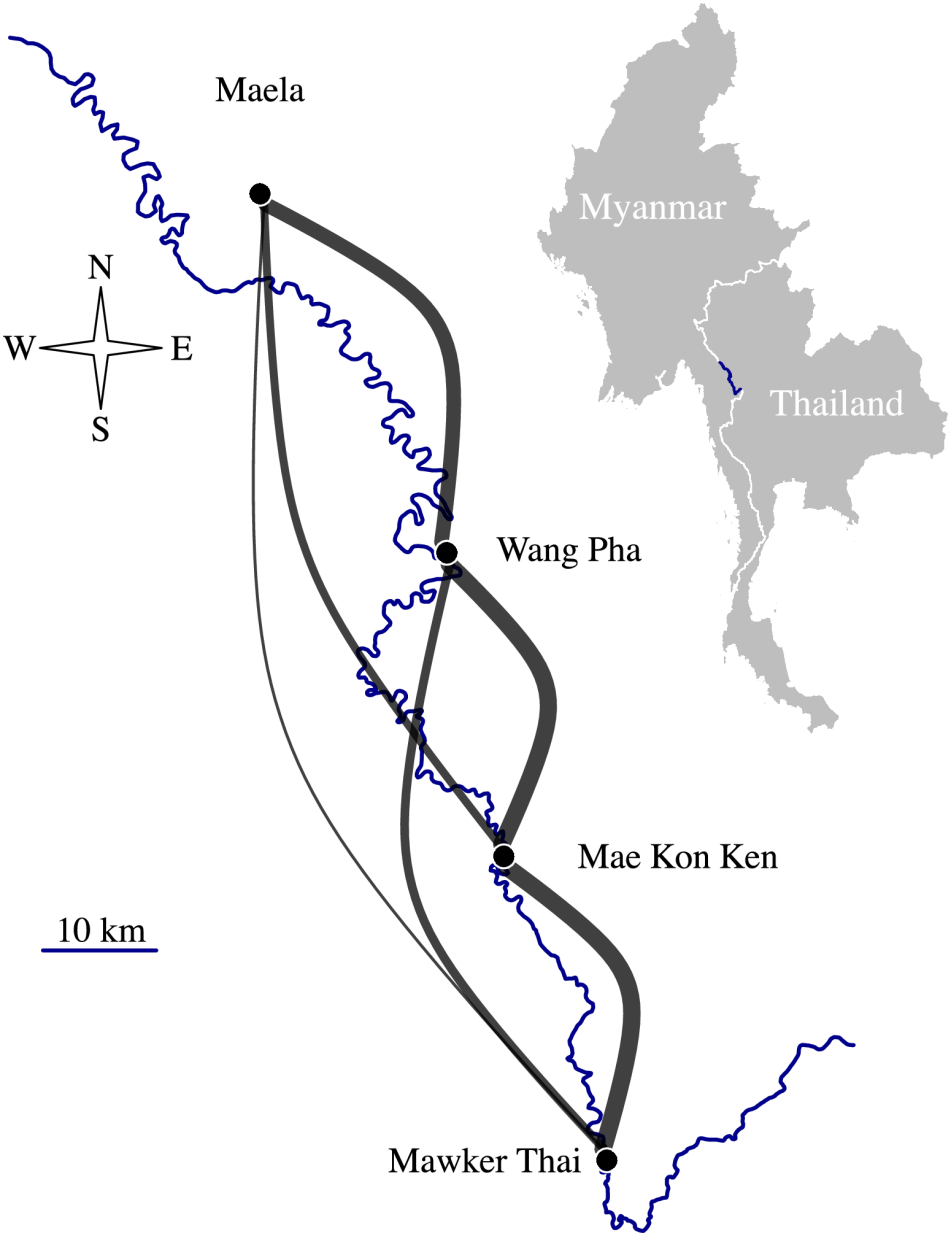
SMRU clinics on the Thai-Myanmar border. The border between Myanmar and Thailand (also the Moei river) is depicted in blue. Grey edges are proportional to inter-clinic proportions of highly related barcode parasite sample pairs (pairs with ${\hat{\pi }}_{IBD}>0.5$). Latitudes and longitudes, respectively, are: 17.128107, 98.382152 (Maela); 16.83014, 98.53737 (Wang Pha); 16.5781479, 98.5846176 (Mae Kon Ken); 16.3258896, 98.670166 (Mawker Thai).

To explore the utility of IBD for estimating connectivity between very local parasite populations, we analyzed one of the largest joint data sets of both genotyping and sequencing data, collected between 2001–2014 from four Shoklo Malaria Research Unit (SMRU) clinics on the Thai-Myanmar border (Fig 1). The border is characterized by mobile migrant populations, villagers, and refugees from Myanmar, and is an area of low and declining malaria transmission [41–43]. This region is therefore representative of many near-elimination settings where remaining pockets of transmission are often found in border areas, and where human mobility is potentially difficult to measure for political or logistic reasons. Here, we focus specifically on measures that capture connectivity: *F*_*ST*_ between population pairs and relatedness between parasite sample pairs. We show that, unlike *F*_*ST*,_ IBD-based relatedness decreases significantly over inter-clinic distance. Importantly, where a tradeoff must be made between sequencing effort and sample sizes, we show that robust spatial trends can be recovered using 93-SNP barcodes, providing a cheap and simple approach to implementing these analyses in the field.

## Results

The genomic data included 1173 single-infection parasite samples genotyped at 93 SNPs [44,45], henceforth referred to as barcode data, and 178 single-infection parasite samples for which whole genome sequences (WGS) were available, henceforth referred to as WGS data [40]. The combined SMRU catchment area spans approximately 120 km of the border, with a population that includes villagers, mobile migrant workers, and refugees entering Thailand from Myanmar [41,44,46]. Previous analyses of these data have shown that despite a decrease in *P*. *falciparum* multiple-genotype infections over the last decade, there has been no evidence of a change in genetic diversity, geographic divergence or effective population size (see S1 Table), likely due to extensive human mobility [45].

### Divergence based on *F*_*ST*_ does not correlate with inter-clinic distance

**Fig 2:**
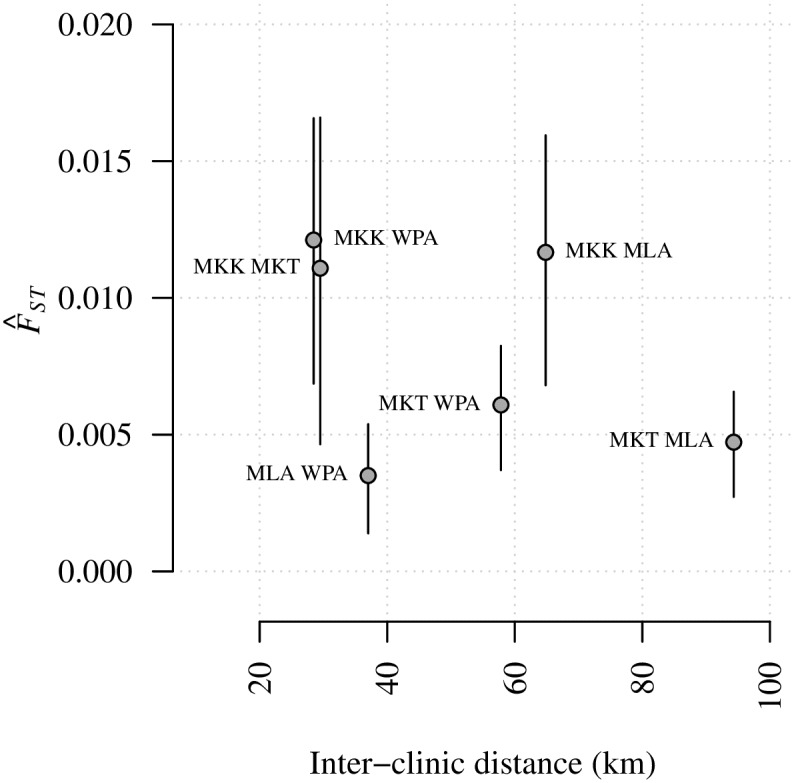
*F*_*ST*_ estimates based on 2001–2014 barcode data plotted with respect to inter-clinic distance. Annotations refer to site comparisons using abbreviated clinic names (MLA for Maela, 212 parasite samples; WPA for Wang Pha, 457 parasite samples; MKK for Mae Kon Ken, 116 parasite samples; and MKT for Mawker Thai, 388 parasite samples). All parasite samples were single-infection. For a clinic pair, A and B say, the *F*_*ST*_ estimate was based on *n*_A_ + *n*_B_ parasite samples, where *n* denotes the number of parasite samples per clinic. Error bars represent 95% confidence intervals based on bootstrapping over SNPs.

**Fig 3:**
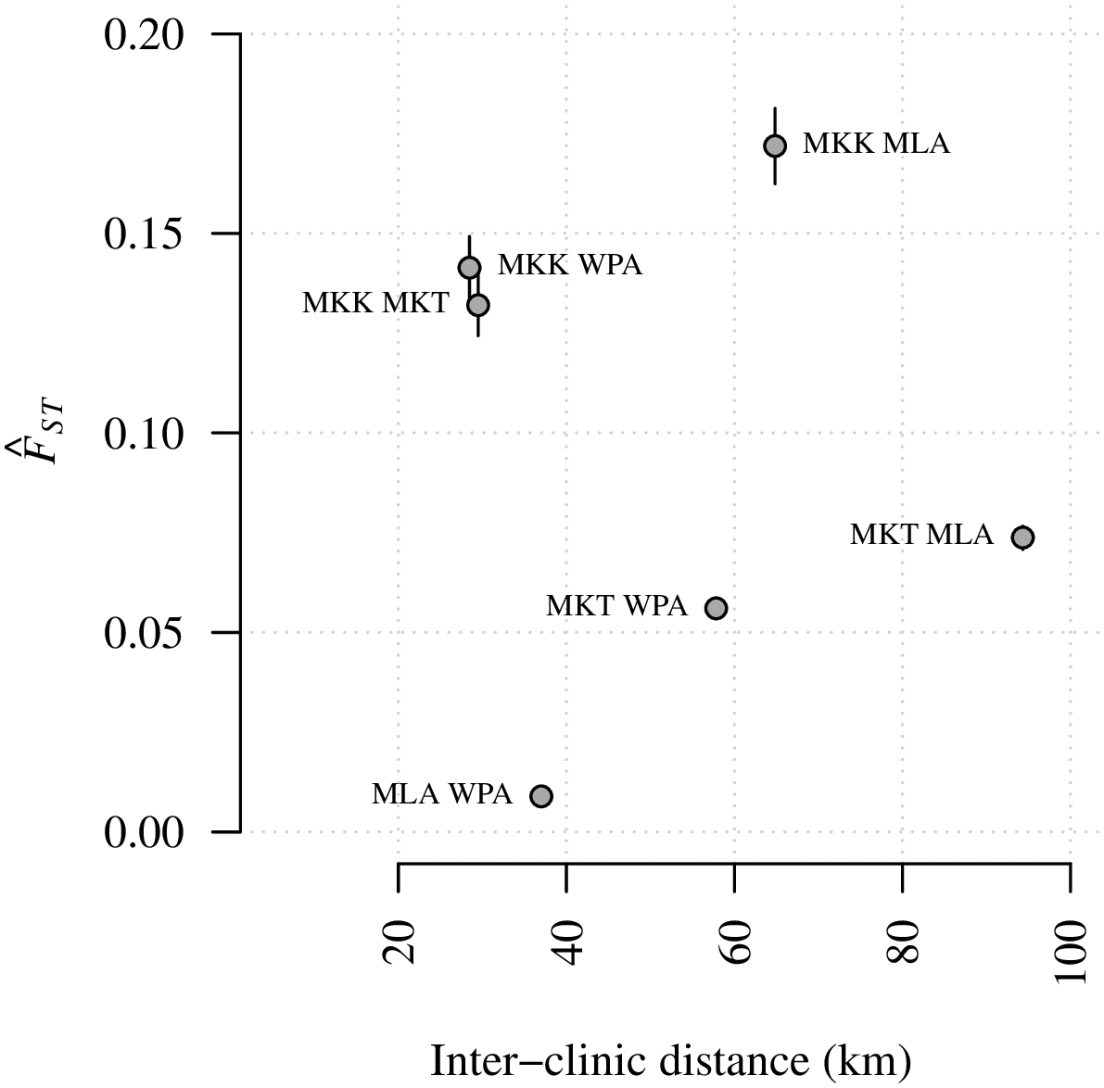
*F*_*ST*_ estimates based on 2001–2014 WGS data plotted with respect to inter-clinic distance. Annotations refer to site comparisons using abbreviated clinic names (MLA for Maela, 55 parasite samples; WPA for Wang Pha, 103 parasite samples; MKK for Mae Kon Ken, 4 parasite samples; and MKT for Mawker Thai, 16 parasite samples). All parasite samples were single-infection. For clinic pair, A and B say, the *F*_*ST*_ estimate was based on *n*_A_ + *n*_B_ parasite samples, where *n* denotes the number of parasite samples per clinic. Error bars represent 95% confidence intervals, based on boostrapping over SNPs.

First we explored spatial structure between parasites collected from different clinics on the Thai-Myanmar border using *F*_*ST*_, a standard measure of divergence between populations, and one that has been applied frequently in the context of malaria. *F*_*ST*_ estimates were calculated using Hudson’s estimator [47–49], which is recommended for small and unequal sample sizes [49,50]. Estimates based on barcode and WGS data across all available years were statistically different from zero with p-values < 0.001 (Tables A and B of S1 Text). Those based on barcode data were low (Fig 2), indicative of migration between populations. Those based on WGS data were an order of magnitude larger (Fig 3), but there was no evidence of spatial trends between clinics based on either barcode or WGS data (Table 1). We observed a positive correlation between *F*_*ST*_ estimates and within-clinic relatedness based on IBD (Fig G in S1 Text), and this appears to explain not only the difference in estimates using barcode versus WGS, but also the lack of spatial trend (S1 Text).

**Table 1:** Spatial trends in *F*_*ST*_ estimates based on barcode data.

Data	Year/s (no. of parasite samples)	*β estimate (p-value)	†β estimate (p-value)	‡β estimate (p-value)
Barcode	2001–2010 (1173)	-6.16e-05 (0.443)	-6.27e-05 (0.440)	-3.46e-03 (0.376)
Barcode	2008 (410)	-5.69e-05 (0.560)	-5.90e-05 (0.558)	-3.27e-03 (0.524)
Barcode	2009 (256)	1.27e-04 (0.443)	1.33e-04 (0.440)	5.18e-03 (0.575)
Barcode	2010 (152)	9.40e-06 (0.954)	7.92e-06 (0.957)	-7.09e-04 (0.946)
WGS	2001–2010 (178)	-6.16e-05 (0.443)	-6.27e-05 (0.440)	-3.46e-03 (0.376)
WGS	2014 (44)	-5.69e-05 (0.560)	-5.90e-05 (0.558)	-3.27e-03 (0.524)

*β represent spatial trends based on untransformed variables;

†β and ‡β represent spatial trends based on transformations that are linearly related under one and two-dimensional models of isolation by distance, respectively [51]. P-values are two-tailed and exact, based on all 6! = 720 permutations of the six inter-clinic *F*_*ST*_ estimates per trend.

### Relatedness based on IBD decreases with inter-clinic distance

**Fig 4:**
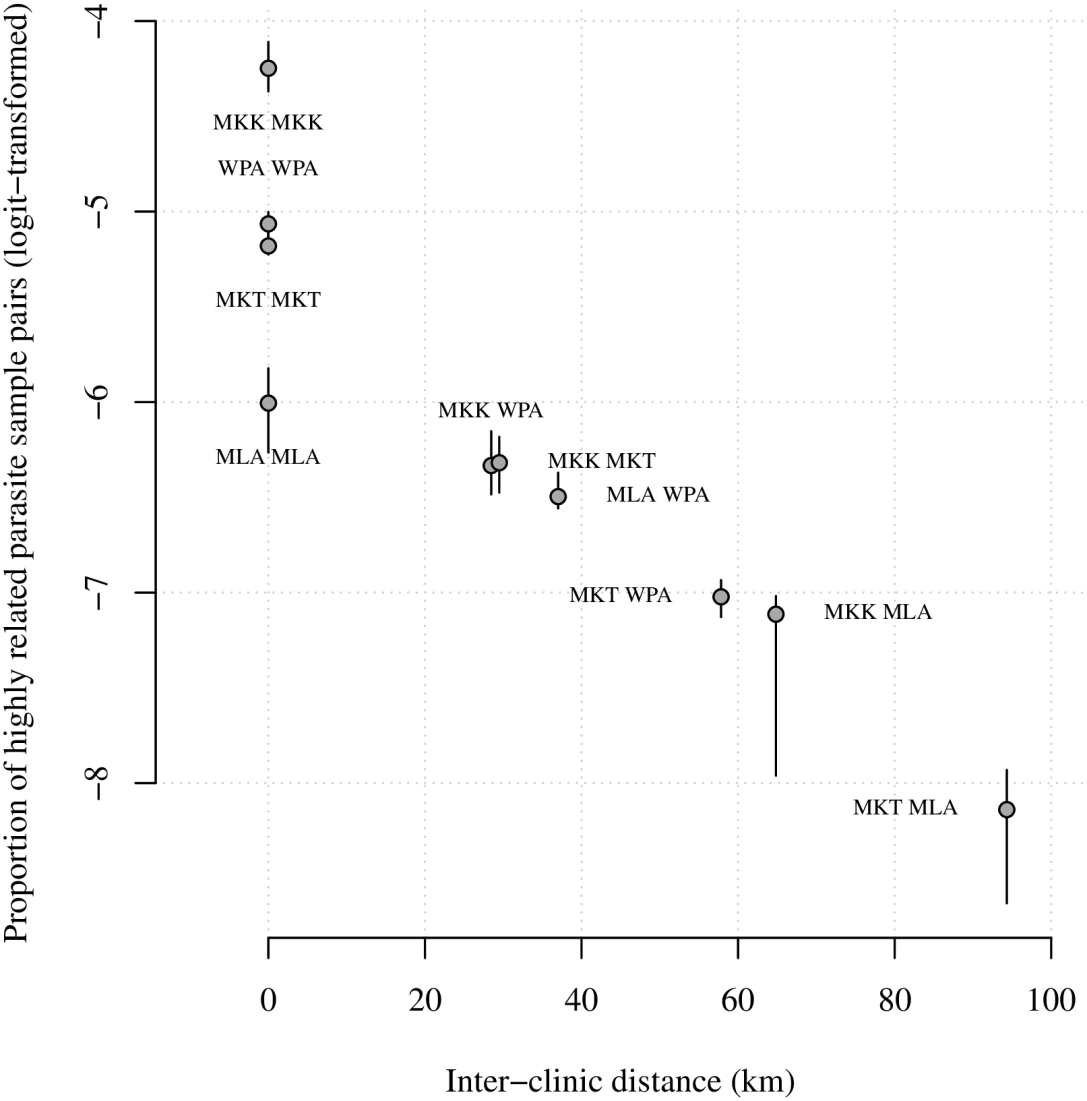
Logit-transformed proportions of highly related 2001–2014 barcode parasite sample pairs with respect to inter-clinic distance. Annotations refer to site comparisons using abbreviated clinic names (MLA for Maela, 212 parasite samples; WPA for Wang Pha, 457 parasite samples; MKK for Mae Kon Ken, 116 parasite samples; and MKT for Mawker Thai, 388 parasite samples). All parasite samples were single-infection. For inter-clinic pair A and B say, the proportion was based on *n*_A_ × *n*_B_ parasite sample pairs, where *n* denotes the number of parasite samples per clinic. Error bars represent 95% confidence intervals based on bootstrapping over highly related parasite sample pair labels (equal to 1 if ${\hat{\pi }}_{IBD}>0.5$ and 0 otherwise), and are therefore zero where there are no ${\hat{\pi }}_{IBD}>0.5$.

**Fig 5:**
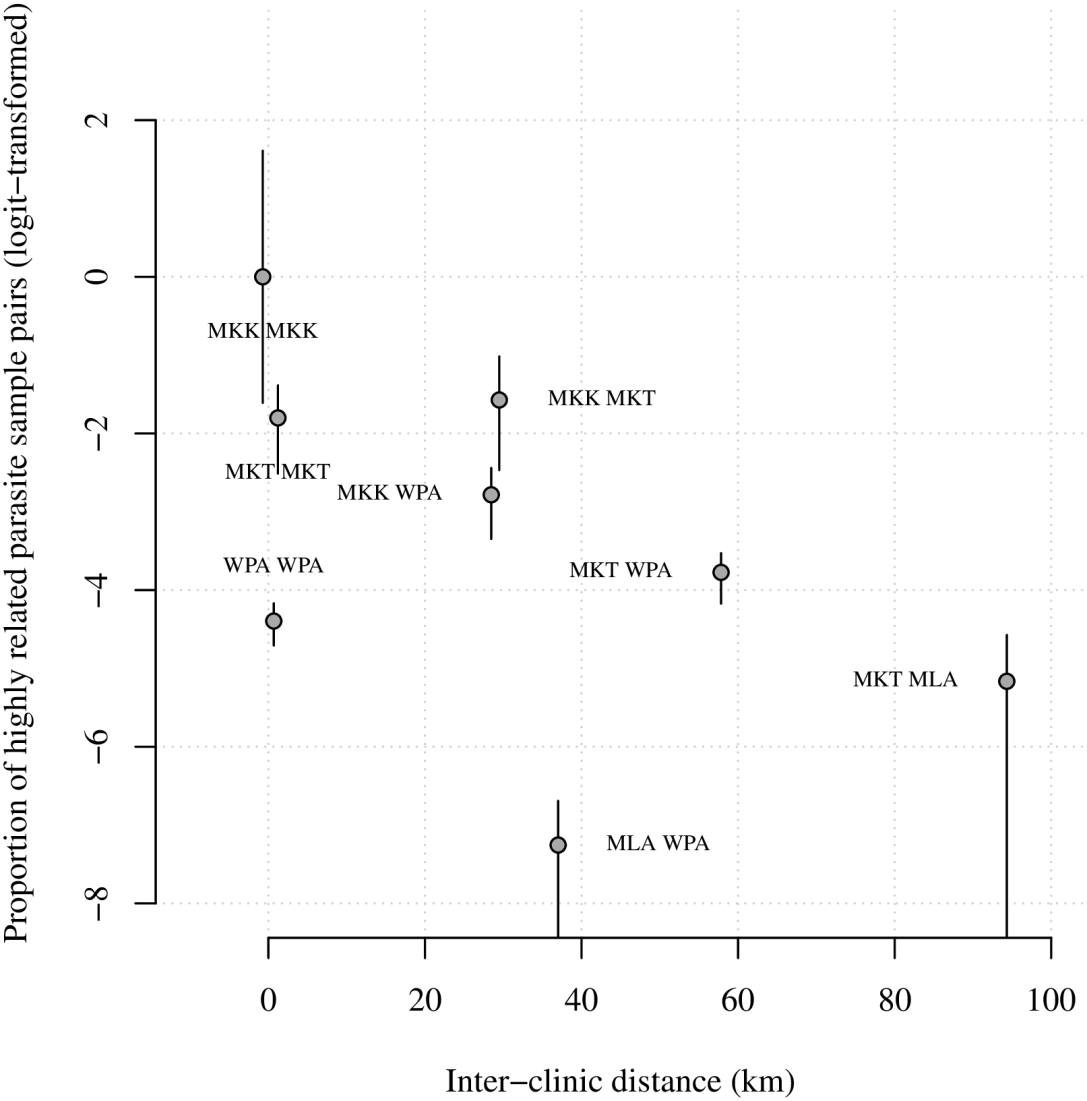
Logit-transformed proportions of highly related 2001–2014 WGS parasite sample pairs plotted with respect to inter-clinic distance. Annotations refer to site comparisons using abbreviated clinic names (MLA for Maela, 55 parasite samples; WPA for Wang Pha, 103 parasite samples; MKK for Mae Kon Ken, 4 parasite samples; and MKT for Mawker Thai, 16 parasite samples). All parasite samples were single-infection. For inter-clinic pair A and B say, the proportion was based on *n*_A_ × *n*_B_ parasite sample pairs, where *n* denotes the number of parasite samples per clinic. Error bars represent 95% confidence intervals based on bootstrapping over highly related parasite sample pair labels (equal to 1 if ${\hat{\pi }}_{IBD}>0.5$ and 0 otherwise), and are therefore zero where there are no ${\hat{\pi }}_{IBD}>0.5$.

We define relatedness using the expected fraction IBD, ${\hat{\pi }}_{IBD}$, a probabilistic measure of the fraction of the genome that a pair of parasites inherited from a recent common ancestor [52]. For a given pair of clinics (e.g. Maela and Wang Pha) we obtained a single *F*_*ST*_ estimate versus many ${\hat{\pi }}_{IBD}$ (*n*_Maela_ × *n*_Wang Pha,_ where *n* denotes the number of parasite samples per clinic). For comparison with *F*_*ST*_ estimates, we plotted proportions of highly related parasite sample pairs (those with ${\hat{\pi }}_{IBD}>0.5$) within and across clinics (Figs 4 and 5 and Fig O and P of S2 Text). However, to leverage the wealth of information across the many parasite sample pairs, spatial trends were estimated using individual ${\hat{\pi }}_{IBD}$. Specifically, we regressed highly related parasite sample pair labels (equal to one if ${\hat{\pi }}_{IBD}>0.5$ and zero otherwise) onto spatial and temporal predictors within a logistic regression framework (see Materials and methods).

Unlike *F*_*ST*_, IBD-based relatedness decreased with inter-clinic distance (Figs 1, 4 and 5), even after adjusting for heterogeneous temporal sampling within the regression model (Tables 2 and 3). Considering barcode data collected from 2001–2010, highly related parasite sample pairs were negatively associated with distance (km) both before (β^unadjusted^ ΔDistance = -0.026, p-value = 0.002) and after (β^adjusted^ ΔDistance = -0.023, p-value = 0.002) adjusting for temporal differences between parasite sample collection dates (Table 2). The spatial trend was of the same order as the temporal trend (β^adjusted^ ΔWeeks = -0.021, p-value = 0.002). The impact of distance decreased with time, but the interaction was very small (β^adjusted^ ΔWeeks × ΔDistance = 0.0001, p-value = 0.002). Importantly, the spatial and temporal trends were also negative upon exclusion of repeat barcodes within clinics (Table C of S2 Text).

**Table 2:** Trends in highly related barcode parasite sample pairs.

Predictor	Year 2001–2010 (1173 parasite samples)		Year 2008 (410 parasite samples)		Year 2009 (256 parasite samples)		Year 2010 (152 parasite samples)	
	β^unadjusted^ (p-value)	β^adjusted^ (p-value)	β^unadjusted^ (p-value)	β^adjusted^ (p-value)	β^unadjusted^ (p-value)	β^adjusted^ (p-value)	β^unadjusted^ (p-value)	β^adjusted^ (p-value)
**Intercept**	-5.54e+00 (1.001)	-4.64e+00 (1.001)	-5.62e+00 (0.002)	-5.40e+00 (0.047)	-4.25e+00 (0.992)	-4.00e+00 (0.992)	-4.19e+00 (0.997)	-3.89e+00 (0.976)
**ΔDistance**	-2.61e-02 (0.002)	-2.27e-02 (0.002)	-9.29e-03 (0.004)	-1.25e-02 (0.004)	-2.87e-02 (0.002)	-3.07e-02 (0.002)	-2.97e-02 (0.002)	-5.28e-02 (0.002)
**Maela**	-4.63e-01 (0.008)	1.33e-01 (0.387)	1.08e+00 (0.090)	9.94e-01 (0.087)	-4.25e-01 (0.507)	-4.21e-01 (0.488)	-6.39e-01 (0.500)	-8.11e-01 (0.405)
**Wang Pha**	4.78e-01 (0.002)	2.71e-01 (0.011)	9.74e-01 (0.002)	9.56e-01 (0.002)	8.40e-02 (0.719)	3.83e-02 (0.877)	-4.83e-01 (0.326)	-9.41e-01 (0.067)
**Mae Kon Ken**	1.30e+00 (0.002)	1.04e+00 (0.002)	1.88e+00 (0.003)	1.88e+00 (0.003)	3.85e-01 (0.357)	3.98e-01 (0.372)	-1.44e+01 (0.098)	-1.47e+01 (0.052)
**Mawker Thai**	3.62e-01 (0.002)	9.34e-01 (0.002)	2.05e+00 (0.002)	2.08e+00 (0.002)	8.77e-01 (0.007)	9.31e-01 (0.004)	-2.17e-01 (0.729)	-5.44e-01 (0.444)
**Season**	NA (NA)	2.22e-01 (0.003)	NA (NA)	1.18e-01 (0.506)	NA (NA)	8.22e-02 (0.696)	NA (NA)	1.25e+00 (0.007)
**ΔWeeks**	NA (NA)	-2.05e-02 (0.002)	NA (NA)	-1.64e-02 (0.018)	NA (NA)	-3.25e-02 (0.007)	NA (NA)	-5.13e-02 (0.046)
**ΔWeeks × Season**	NA (NA)	-1.27e-03 (0.002)	NA (NA)	-7.30e-03 (0.611)	NA (NA)	1.07e-02 (0.529)	NA (NA)	-9.03e-02 (0.029)
**ΔWeeks × ΔDistance**	NA (NA)	1.17e-04 (0.002)	NA (NA)	2.19e-04 (0.134)	NA (NA)	2.22e-04 (0.314)	NA (NA)	1.60e-03 (0.002)

P-values are two-tailed Monte Carlo estimates based on 1000 permutations of highly related parasite sample pair labels (equal to 1 if ${\hat{\pi }}_{IBD}>0.5\;$ and 0 otherwise).

**Table 3:** Trends in highly related WGS parasite sample pairs.

Predictor	Year 2001–2014 (178 parasite samples)			Year 2014 (44 parasite samples)	
	β^unadjusted^ (p-value)	β^adjusted^ (p-value)	β^adjusted 2014^ (p-value)	β^unadjusted^ (p-value)	β^adjusted^ (p-value)
**(Intercept)**	-4.65e+00 (0.355)	-3.90e+00 (0.936)	-4.44e+00 (0.565)	-1.05e+00 (1.000)	-1.85e+01 (0.002)
**ΔDistance (km)**	-8.60e-04 (0.885)	-7.58e-03 (0.373)	-2.00e-02 (0.035)	-2.33e-02 (0.002)	-2.64e-02 (0.002)
**Maela**	-1.49e+01 (0.002)	-1.56e+01 (0.002)	-1.54e+01 (0.002)	-1.55e+01 (0.012)	-1.80e+01 (0.002)
**Wang Pha**	2.53e-01 (0.403)	-7.68e-01 (0.027)	-2.52e-01 (0.490)	-4.40e-01 (0.321)	-4.42e-01 (0.330)
**Mae Kon Ken**	4.65e+00 (0.950)	2.00e+00 (1.000)	1.05e+00 (1.001)	1.05e+00 (0.619)	1.07e+00 (0.638)
**Mawker Thai**	2.85e+00 (0.310)	1.84e-01 (0.809)	-7.61e-01 (0.345)	-7.47e-01 (0.102)	-9.49e-01 (0.039)
**Season**	NA (NA)	1.99e+00 (0.002)	1.48e+00 (0.002)	NA (NA)	1.82e+01 (0.002)
**ΔWeeks**	NA (NA)	-1.48e-02 (0.002)	-1.48e-02 (0.002)	NA (NA)	-1.38e-02 (0.779)
**ΔWeeks × Season**	NA (NA)	6.61e-04 (0.514)	4.17e-03 (0.002)	NA (NA)	-1.11e-01 (0.066)
**ΔWeeks × ΔDistance**	NA (NA)	-1.26e-04 (0.002)	2.92e-05 (0.090)	NA (NA)	5.95e-04 (0.218)
**Year**	NA (NA)	NA (NA)	1.97e+00 (0.005)	NA (NA)	NA (NA)

P-values are two-tailed Monte Carlo estimates based on 1000 permutations of highly related parasite sample pair labels (equal to 1 if ${\hat{\pi }}_{IBD}>0.5\;$ and 0 otherwise).

WGS data showed similarly negative spatial trends (Fig 5 and Table 3). Since contemporaneous WGS data from all four clinics was only available in 2014, we excluded prior years in the regression model, and found significant negative trends both before (β^unadjusted^ ΔDistance = -0.023, p-value = 0.002) and after (β^adjusted^ ΔDistance = -0.026, p-value = 0.002) adjustment for temporal differences between parasite sample collection dates. The trend based on data across all years was also significant, but only after adjustment for an overall increase in highly related parasite sample pairs in 2014 (β^adjusted 2014^ ΔDistance = -0.020, p-value = 0.035).

### Independent evidence of spatial trends present in the data

IBD-based analyses recovered spatial and temporal trends where *F*_*ST*_ did not. Together with evidence of phenotypic differences in parasites across SMRU clinics [44], our results suggest IBD-based analyses are sensitive to local spatial genetic structure. To further validate these findings, we used ChromoPainter [26] to estimate average numbers of segments donated and received between parasites from different clinics, hereafter referred to as clinic-averaged co-ancestries (S3 Text). Like IBD-based analyses, ChromoPainter accounts for recombination [26], and it has been used to study malaria parasite populations in Cambodia [20].

Clinic-averaged WGS co-ancestry estimates showed a similar pattern as proportions of highly related parasite pairs, declining with inter-clinic distance (Fig B in S3 Text), and thereby supporting the spatial genetic structure observed in our IBD-based analyses.

Clinic-averaged barcode co-ancestry estimates declined with distance only when considering both within-clinic and inter-clinic estimates (Fig D in S3 Text). Plots of pairwise estimates suggest that differences between inter-clinic averaged barcode co-ancestry estimates were unresolved because the range of estimates was narrow compared with ${\hat{\pi }}_{IBD}$ (Fig E in S3 Text), despite positive correlation with ${\hat{\pi }}_{IBD}$ (Fig F in S3 Text). This is expected, however, since ChromoPainter is not intended for sparse barcode data.

### Calculating sample sizes and sequencing effort required to recover spatial trends

To assess the sample sizes required to measure connectivity between proximal sites using IBD, we re-estimated trends using random subsets of the data across all years under temporally adjusted logistic regression models. Subsets ranged in size from 50 to 1171 barcode parasite samples, and from 50 to 176 WGS parasite samples. We also considered the impact of sequencing fewer SNPs, since many studies use a 24-SNP barcode (e.g. [38]). We use an ideal set of 24 SNPs with high minor allele frequency (Fig A in S4 Text), following the experimental design of a molecular barcode [53]. Our 24-SNP barcode results therefore represent a best-case scenario; “true” barcodes, which are constructed *a priori*, will almost surely deviate from this ideal due to spatiotemporal variations in minor allele frequencies.

**Fig 6:**
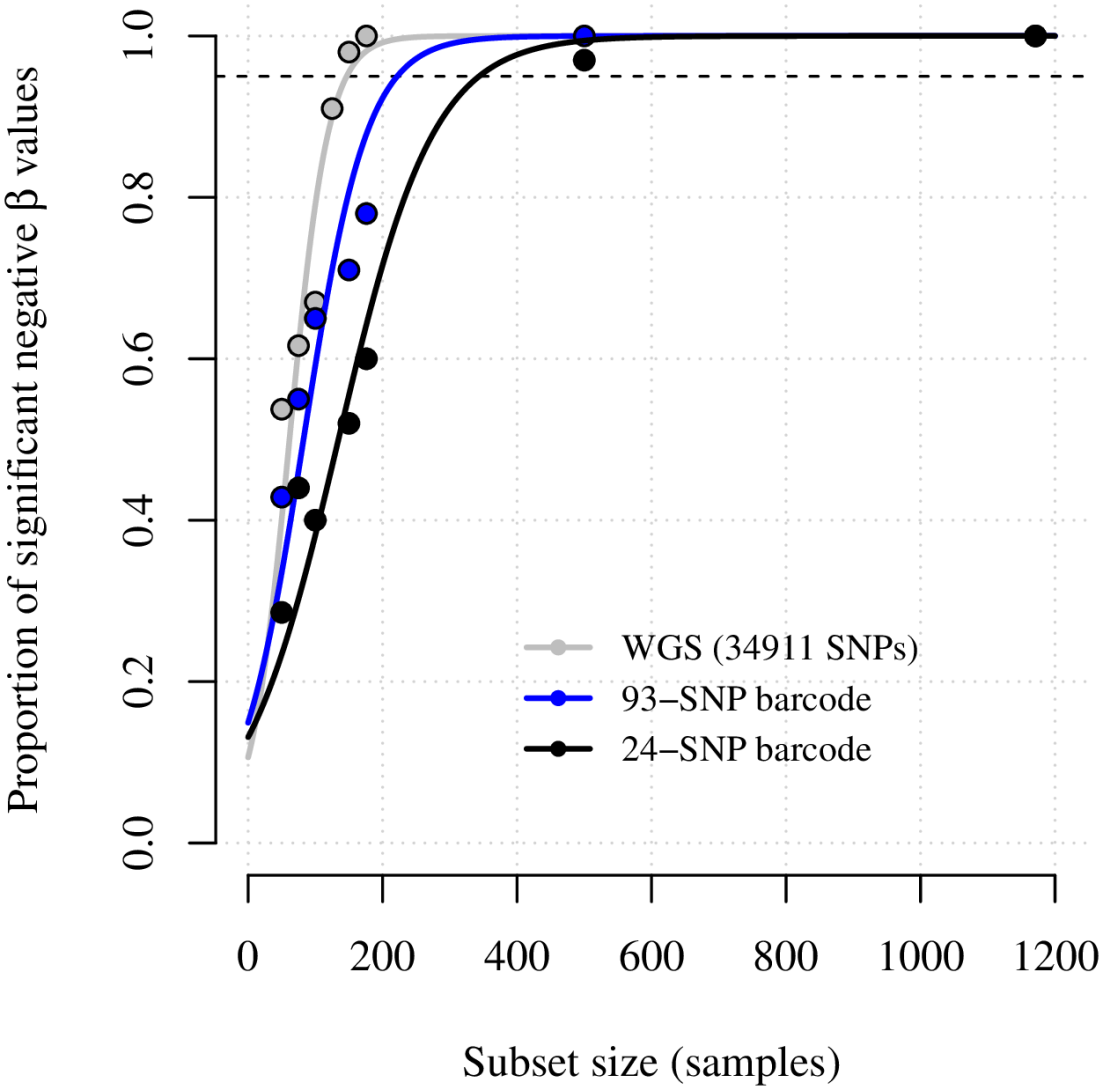
Proportions of significant negative spatial trend estimates with respect to data subset sample size. Spatial trend estimates were based on regression of highly related parasite sample pair labels (equal to 1 if ${\hat{\pi }}_{IBD}>0.5\;$ and 0 otherwise) onto distance, within temporally adjusted models (β^adjusted^ ΔDistance (km) for barcode data, and β^adjusted 2014^ ΔDistance (km) for WGS data).

Fig 6 shows the relationship between sample size and significant negative spatial trends observed for different sequencing approaches. We estimated that approximately 147 WGS parasite samples, 222 93-SNP barcode parasite samples, and 344 24-SNP barcode parasite samples were sufficient to recover significant negative trends 95% of the time (Table 4). It is important to note, however, that spatial trend estimates based on only 24-SNPs converged to -0.004, whereas equivalent estimates based on 93 or more SNPs converged to -0.023 and -0.020, respectively (Tables 2 and 3). The comparatively low spatial trend estimate based on only 24 SNPs was not unexpected. Previous studies have shown barcode size limits resolution of low genome wide identity [38], while simulated data show rapidly decreasing accuracy in ${\hat{\pi }}_{IBD}$ with fewer SNPs [52]. Moreover, due to the bounded nature of ${\hat{\pi }}_{IBD}$, very wide error at low IBD is liable to result in a bias towards 24-SNP ${\hat{\pi }}_{IBD}\;$ that exceed genome-wide ${\hat{\pi }}_{IBD}$ and the 0.5 threshold for highly related parasite sample pairs (Fig B in S4 Text), leading to poor resolution of spatiotemporal trends. We therefore do not recommend the use of 24 SNP barcodes for IBD-based analyses.

**Table 4:** Models fit to significant negative proportions of spatial trends.

Data type	Parameter estimates, for proportions fit to sample subset size *x*, *f*(*x*) = *e*^*cx*−3.66^/(*d* + *e*^*cx*−3.66^)	Correlation between data and fitted points, *f*(*x*)	Predicted number of parasite samples† to recover a statistically significant negative trend 95% of the time, *f*^-1^(0.95)
**WGS**	c = 0.03, d = 0.22	0.98	147
**93-SNP Barcode**	c = 0.02, d = 0.15	0.97	222
**24-SNP Barcode**	c = 0.01, d = 0.17	0.98	344

^†^All parasite samples were single-infection.

The 93 SNP barcode provided a surprisingly robust estimate of geographic structuring, however. Like chromosome painting methods [26], IBD-based analyses capture information based on dependence between SNPs [35]. Although inter-SNP distances are large on the 93-SNP barcode, barcode SNPs are mostly dependent under hmmIBD because the recombination rate is low (Fig C in S4 Text).

## Discussion

Despite the potential utility of genetic data for resolving fine-scale differences in connectivity among proximal populations in *P*. *falciparum* and other species, there are minimal guidelines about how to quantify gene flow between proximal locations. Here we show that IBD-based relatedness provides a more robust measure of local spatial structure than *F*_*ST*_. Moreover, where a trade-off must be made between sample size and sequencing effort, 93 SNPs were sufficient to recover robust spatial trends using relatively few additional parasite samples compared with WGS. This is an important practical insight given the wide availability of historical barcode data, and the relative cost-effectiveness and ease of generating barcode data compared to whole genome sequences. We therefore propose that IBD-based relatedness is not only a useful metric of gene flow between proximal populations, but also that it can be efficiently estimated using 93-SNP barcodes, which are inexpensive and can be generated from parasite DNA extracted from dried blood spots on filter papers.

*F*_*ST*_ estimates were strongly affected by clinics characterized by highly related parasites, and this association appeared to overwhelm spatial trends. With a view to monitoring malaria parasite populations, we consider this apparent sensitivity of *F*_*ST*_ potentially problematic for its routine use. This is especially true of regions of declining transmission, where fewer infections go together with the emergence of increasingly clonal hotspots. Although IBD-based analyses were not completely impervious to high within-clinic relatedness, they retain their ability to recover spatial trends. Furthermore, since IBD-based analyses allow explicit estimation of within-clinic relatedness, its impact on relatedness across populations can be assessed.

We estimated that approximately 147 single-infection WGS parasite samples, or 222 single-infection 93-SNP barcode parasite samples, were required to recover robust spatial trends. In very low transmissions settings, such as those where the number of cases has dropped below the World Health Organization’s pre-elimination threshold of 1 infection per 1000 persons per year, the number of parasite samples required to estimate spatial trends would in many cases exceed the number of cases. Here, ${\hat{\pi }}_{IBD}$ could still be used to assess relatedness between individual cases and suspected source populations, which may be critical given the World Health Organization’s definition of elimination, which requires no local cases for 3 years, but allows for imported ones. Although our analyses suggest 93-SNP barcodes are sufficient to recover robust spatial trends at the population level, we do not recommend using 93-SNP barcodes for standalone analyses of individual parasite sample pairs due to large expected error in that application ([52] and Fig B in S4 Text). In high transmission settings overall relatedness will likely decrease due to increased recombination. To account for low population-level relatedness, one could genotype more SNPs and decrease the threshold for highly related sample pairs. Ideally one would also use a model capable of estimating IBD from complex samples of multiple-genotype infections, since these are liable to increase in abundance with transmission [54]. Henden et al. recently proposed an IBD model that can support parasite samples with one or two parasite strains [31], although it doesn’t currently output ${\hat{\pi }}_{IBD}$ directly. Models capable of supporting parasite samples with three or more strains are lacking.

Although this combined set of barcode and WGS data is one of the largest of its kind, the sampling design was not intended for the question at hand. However, despite uneven sampling in time and space we find evidence of spatial genetic structure on the Thai-Myanmar border, which is consistent with earlier reports of phenotypic differences between parasites from different clinics [44]. Evidence of spatial structure is also supported by results from an independent method ChromoPainter [26]. Akin to IBD-based analyses, ChromoPainter leverages the wealth of haplotypic information in WGS data, but struggles to resolve variation in 93-SNP barcode data, for which it was never intended. Regardless of the method used, evidence of spatial structure calls for a better understanding of the drivers that sustain spatial trends. Epidemiological models parameterized by human mobility data have been used to estimate the spatial spread of pathogens in some cases [55–58], but data on human migration are difficult to obtain, particularly in sparsely populated areas and in regions near international borders, where there are political sensitivities around measuring migration.

Analyses of spatial genetic structure are common beyond malaria (e.g. studies of pollen dispersal [59–62]). Measures used are largely variants of IBS and therefore sensitive to the marker system and reference population [62]. Unlike IBS-based methods, IBD-based methods explicitly account for the marker scheme by conditioning on allele frequencies. They could thus prove useful as IBS surrogates in spatial studies of other recombining organisms [63–66].

In summary, we propose that IBD-based relatedness will prove useful in the malaria field and in other infectious disease systems to compare data collected from local sites, from areas with more complex topologies, and where data are available, to compare human and parasite movement. IBD-based relatedness could also prove useful beyond epidemiological applications to complement spatial analyses of other sexually recombining organisms.

## Materials and methods

### Data

The barcode data were generated as part of a longitudinal trial of artemisinin resistance and its genetic heritability [44], then later reanalyzed to identify correlates of declining malaria transmission [45]. Full details of sample collection and laboratory methods can be found in [44] and [45]. Briefly, 1173 filter paper blood spots were collected between 2001 and 2010 from hyper-parasitaemic patients (> 4% infected red blood cells) with uncomplicated *P*. *falciparum* malaria presenting at four SMRU clinics on the Thai-Myanmar border (Fig 1). DNA extracted using a two-step protocol was successfully genotyped at 93 SNPs using the Illumina GoldenGate platform. The 93 SNPs were distributed across the *P*. *falciparum* genome (Fig A in S4 Text), but not in regions likely under strong selection (supporting information of [45]). In total, 558 parasite samples were considered multiple-infection (containing more than one *P*. *falciparum* genotype), based on 6 or more heteroallelic genotyping outcomes [45], while 1173 were considered single-infection. Analyses in this study were based on single-infection parasite samples only (S2 Table). The WGS data were generated from 178 parasite samples collected between 2001–2014 from the same four clinics (S3 Table). Full details of sample collection and sequencing workflow can be found in [40]. Briefly, parasite samples collected prior to 2010 were derived from a single-infection subset of the aforementioned dried blood spots, selected such that no two showed identical 93-SNP genotypes, and sequenced following hybrid selection on an Illumina HiSeq 2500 platform. Parasite samples collected from 2010 onwards were collected as venous blood and directly sequenced on an Illumina HiSeq 2500 platform following leukocyte depletion. As described by Cerqueira and collegues [40], reads were aligned to the *P*. *falciparum* 3D7 v3 reference genome, genotypes called and sites filtered. Those removed included heterozygous sites, indels, sites with QUAL < 60, GQ < 30, polymorphic sites located in pericentromeric, subtelomeric and hypervariable regions, and sites occurring in genes belonging to large antigenic gene families. In addition to the sites listed above, we removed 121 sites with reference or alternative allele assignments indicating potential indels, monomorphic sites, sites lacking genotype calls in 20% or more of the parasite samples, and mitochondrial and apicoplast sites, leaving a total of 34911 polymorphic biallelic SNPs.

### Data analyses

Aside from IBD estimates generated using hmmIBD (v2.0.0) [52], and co-ancestry estimates generated by the ChromoPainter package within fineSTRUCTURE version 2 [26], all data analyses were performed in R [67]. P-values less than 0.05 were considered significant and were calculated by permutation. They were exact if the number of possible permutations was less than 1000, otherwise they were Monte Carlo estimates [68]. Monte Carlo p-values can overestimate true p-values [69]; however, overestimation is small when the number of randomly sampled permutations, *n*, is large (at least 99 [68]). We use *n* = 100 when assessing the sensitivity of spatial trends to sample size (see below) and in sensitivity tests (Fig Q in S2 Text), otherwise *n* = 1000. All p-values were two-tailed, with the exception of those for *F*_*ST*_ estimates (Tables 1 and 2 of S1 Text), since *F*_*ST*_ is non-negative. Two-tailed p-values were calculated by summation over left and right-hand tails.

#### Estimates of divergence between population pairs

Pairwise divergence estimates were based on Wright’s fixation index (*F*_*ST*_) [29,30]. To estimate *F*_*ST*_, we used Hudson’s estimator [48], whose explicit formulation can be found in [47] and [49]. We chose this particular estimator because it is recommended for small and unequal sample sizes [49,50], and is asymptotically consistent in the number of loci [47,49]. The significance of each pairwise *F*_ST_ estimate was tested by 1000 permutations of the clinic labels, thereby assuming *F*_*ST*_ = 0 under the null hypothesis. Following [70], 95% confidence intervals were obtained by bootstrapping over SNPs 1000 times.

#### Estimates of relatedness between parasite sample pairs

Relatedness estimates were based on the expected fraction IBD, ${\hat{\pi }}_{IBD}$, a probabilistic measure of the fraction of the genome inherited by a pair of parasites from a recent common ancestor. For all pairwise comparisons of parasite samples in the barcode and WGS data sets, we estimated ${\hat{\pi }}_{IBD}$ using hmmIBD [52]. Specifically to estimate ${\hat{\pi }}_{IBD}$, we calculated the posterior probability of the IBD state at the position of each SNP (equation 38 in [71]) using the forward-backward algorithm as described in [71], then averaged these probabilities over the *T* SNPs across the genome,

$${\hat{\pi }}_{\text{IBD}}=\frac{1}{T}\sum_{t=1}^{T}\mathbb{P}\text{(IBD}\;\text{at}\;\text{position}\;t\;\text{|}\;\text{data},\;\text{model}).$$

${\hat{\pi }}_{IBD}$ can be interpreted as the expected number of IBD state assignments over the genome, divided by the number of SNPs, and is thus a measure of the fraction of the genome inherited by a pair of parasites from a recent common ancestor. Unlike estimates based on the Viterbi path [71], it is not dependent on a single sequence assignment. hmmIBD requires the position of each SNP measured in base pairs, since the probability of transitioning between IBD and non IBD states is considered a function of the distance between SNPs (S4 Text and [52]). When inferring IBD, sequencing data are sometimes pruned to account for linkage disequilibrium [72]. We did not prune our WGS data, however. hmmIBD also requires point estimates of allele frequencies in order to calculate the probability of observing concordance or discordance between genotype calls across parasite sample pairs. Given little evidence of differences in allele frequency point estimates across clinics or over years (S4 Table), we used allele frequency estimates based on data across all years and clinics for both barcode and WGS data respectively.

#### Highly related parasite sample pairs

To investigate spatial trends we focused on highly related parasite sample pairs (those with ${\hat{\pi }}_{IBD}>0.5$), since they were considered more likely representative of recent migration (S2 Text). The 0.5 threshold was based on empirical density plots of ${\hat{\pi }}_{IBD}$ (Fig A to J in S2 Text). It is approximately equal to the mean relatedness between progeny derived from experimental *P*. *falciparum* crosses [73]. Large translations around 0.5 recovered significant negative spatial trends (see section below for details) over highly related barcode parasite sample pairs (Fig Q in S2 Text, top row), while only small translations around 0.5 recovered significant spatial trends in WGS parasite sample pairs (Fig Q in S2 Text, bottom row).

Logit-transformed proportions of highly related parasite sample pairs were calculated within and across clinics and plotted against inter-clinic distance (non-transformed equivalents can be found in S2 Text). 95% confidence intervals were generated by bootstrapping over highly related parasite sample pair labels (equal to 1 if ${\hat{\pi }}_{IBD}>0.5\;$ and 0 otherwise) 1000 times, and are thus zero where there are no ${\hat{\pi }}_{IBD}>0.5.$

#### Clinic-averaged co-ancestry estimates

To support results based on highly related parasite sample pairs, additional evidence of spatial structure in the data was sought. We used the ChromoPainter package implemented within the standard pipeline of FineSTRUCTURE version 2 (fs-2.1.1) [26], to estimate the co-ancestry matrix of the WGS and barcode data. Linked analyses were performed using a uniform recombfile with recombination rate 7.4 × 10^−7^ Morgans per base pair [74], while unlinked analyses were performed by omitting the recomfile. For both linked and unlinked analyses, we set ploidy equal to one. The co-ancestry matrix contains estimated counts of DNA segments received and donated between parasites, where each SNP is considered an independent segment under the unlinked analysis. To construct clinic-averaged co-ancestry estimates, we averaged the received and donated counts within and across clinics. To explore spatial structure and concordance with IBD-based analyses, clinic-averaged co-ancestry estimates were regressed onto inter-clinic distance, and compared with corresponding estimates based on IBD (S3 Text).

#### Spatial trends in divergence

Divergence trends were assessed by regressing *F*_*ST*_ estimates onto inter-clinic great circle distance in kilometers (km), where great circle distance is the distance between two clinics on the earth’s surface. Specifically, we estimated spatial estimates using untransformed variables following the regression model, *F*_*ST*_ = intercept + (*β × inter-clinic distance) + ε, where *β represents the spatial trend, and ε is assumed to be normally distributed with mean zero and variance σ^2^. In addition, we explored trends in transformed variables following [51]. More specifically, we fit *F*_*ST*_ / (1- *F*_*ST*_) = intercept + (†β × inter-clinic distance) + ε, where †β represents the spatial trend under a one-dimensional model of isolation by distance, and ε is defined above; and *F*_*ST*_ / (1- *F*_*ST*_) = intercept + (‡β × log(inter-clinic distance)) + ε, where ‡β represents the spatial trend under a two-dimensional model of isolation by distance, and ε is defined above. Two-tailed exact p-values were generated using all 6! = 720 permutations of the *F*_*ST*_ estimates, thereby assuming no trend under the null hypothesis.

#### Spatial trends in relatedness

IBD-based relatedness trends were assessed by regressing highly related parasite sample pair labels (equal to 1 if ${\hat{\pi }}_{IBD}>0.5$ and 0 otherwise) onto inter-clinic distance (km) under a logistic model framework with and without temporal predictors as outlined below and in S2 Text. Models fit without temporal predictors included an intercept term, inter-clinic distance (ΔDistance, measured in km as defined above for *F*_*ST*_), and a predictor per clinic (equal to one if both parasite samples within a pair were collected in the corresponding clinic and zero otherwise) to account for variance between clinics at ΔDistance = 0. The structure of the temporally adjusted models was based on a preliminary analysis of eight models fit to barcode data (full details can be found in S2 Text). The eight models were compared using the Akaike information criterion (AIC), a model comparison score that favors comparatively low values [75]. Temporal predictors included ΔTime between parasite sample collection dates measured to the nearest day, week, month or year; Season, equal to one if two parasite samples within a pair were collected in spring, summer or both; and interactions between ΔTime, Season and ΔDistance. The chosen model included four temporal predictors: Season, ΔTime measured in weeks (ΔWeeks), and two interaction terms (ΔWeeks × Season and ΔWeeks × ΔDistance), allowing the impact of season and inter-clinic distance to vary with weeks between parasite sample collection dates. To account for an increase in IBD in 2014 (Fig N in S2 Text), we introduced an additional predictor to models fit to WGS data, year 2014, true if both parasite samples within a pair were collected in 2014 and false otherwise. Regression coefficient estimates fit under models with and without temporal predictors were denoted β^adjusted^ and β^unadjusted^, respectively. Those fit under the model with the additional year 2014 predictor were denoted β^adjusted 2014^. Two-tailed Monte Carlo p-values of the regression coefficients were generated by 1000 permutations of highly related parasite sample pair labels (equal to 1 if ${\hat{\pi }}_{IBD}>0.5$ and 0 otherwise), thereby assuming non-intercept regression coefficients equal to zero under the null hypothesis.

#### The sensitivity of spatial trends to the sample size

Sensitivity to sample size was assessed by sequentially sampling random subsets of the data, and re-estimating spatial trends. Spatial trend estimates were generated under the temporally adjusted logistic regression framework, with the year 2014 predictor for WGS data, thereby accounting for fluctuations in sample sizes over clinics and time induced by random sampling. Random subsets ranged from 50 to 176 parasite samples for WGS data, and 50 to 1171 for barcode data. We also considered a smaller barcode by reanalyzing the barcode data using 24 of the 93 SNPs with the highest minor allele frequencies. For each subset size, subsampling was repeated 100 times. For each random subset, two-tailed Monte Carlo p-values were generated by 100 permutations of the highly related parasite sample pair labels (equal to one if ${\hat{\pi }}_{IBD}>0.5$ and zero otherwise). We then calculated the proportion of negative and significant spatial trend estimates for each subset size, *x*, including only estimates generated from logistic regression analyses that successfully converged, and fit logistic regression curves to the proportions using the nonlinear least squares (Table 4). We added two artificial data points equal to 0.025 at *x* = 0, to constrain curves to tend to 0.025 as *x* tends to 0. Finally, we found the sample sizes corresponding to 95% significant negative spatial trend estimates by inversion of the fitted nonlinear curves.

## Supporting information

S1 Table Summary of previous population genetic analyses of the barcode data in [1] Nkhoma SC, et al. Mol Ecol. 2013; 22(2). **†**Given decline in malaria transmission. Figure references refer to Figures in [1].
(PDF)

S2 Table Single-infection barcode data parasite sample counts per clinic and year. Clinic code: MLA (Maela), WPA (Wang Pha), MKK (Mae Kon Ken) and MKT (Mawker Thai).
(PDF)

S3 Table Single-infection WGS data parasite sample counts per clinic and year. Clinic code: MLA (Maela), WPA (Wang Pha), MKK (Mae Kon Ken) and MKT (Mawker Thai).
(PDF)

S4 Table Numbers of SNPs for which clinic and year variables were significant in a simple linear model. The simple linear model was as follows. Non-reference allele frequency = intercept + β_clinic_ clinic + β_year_ year + ε, where ε was normally distributed with mean 0 and variance σ^2^.
(PDF)

S1 Text Additional details and analyses regarding *F*_*ST*._ (PDF)

S2 TextAdditional details and analyses regarding ${\hat{\pi }}_{IBD}$.(PDF)

S3 Text Additional details and plots of co-ancestry estimates. (PDF)

S4 TextAdditional details and plots of 93 and 24-SNP ${\hat{\pi }}_{IBD}$.(PDF)

S1 Data Barcode data. (TXT)
