# Supplementary material for: Quantifying connectivity between local Plasmodium falciparum malaria parasite populations using identity by descent
Source: PLoS Genet. 2017 Oct 27;13(10):e1007065. doi: 10.1371/journal.pgen.1007065 (PMC5678785; doi:10.1371/journal.pgen.1007065)
Supplement: S1 Table — †Given decline in malaria transmission. Figure references refer to Figures in [1]. (PDF) [file pgen.1007065.s001.pdf]

Correlate	Expectation[†]	Observation
Percentage of multiple-genotype infections (samples with >5% het calls)	Decline	Statistically significant decline from 63-14% from 2000-2010 (Figure 3)
Number of heteroallelic calls per multiple-genotype infection	Decline	Statistically significant decline with year, from 31-20 from 2001-2010 (Figure 4)
Probability of sampling identical MLGs within 14 days in 2001-2004 (t1) versus 2007-2010 (t2)	Increase	Increased (Figure 5c)
Genotypic richness, R (proportion of unique genotypes, measured by the number of distinct MLG divided by sample size)	Decline	Significant decline from 0.94-0.71 from t1 to t2 in both Mawker Thai and Maela (Figure 7b)
Slope, β, of the MLG frequency distribution in t1 versus t2	Decline	Significant decline from 3.39-1.37 from t1 to t2 in both Mawker Thai and Maela (Figure 7c)
Multilocus linkage disequilibrium, I_AS, in t1 versus t2 with and without repeated MLGs	Increase	Increased significantly from 0.0040 to 0.0109 from t1 to t2 in Mawker Thai but not Maela (0.0024 to 0.0025 from t1 to t2)
Genetic diversity (Nei's expected heterozygosity index, H_E)	Decrease	No change (0.427 to 0.429 from t1 to t2)
F_{ST} between Maela and Mawker Thai using Weir & Cockerham estimator	Increase	Insignificant increase from 0.007 to 0.013 from t1 to t2
Short-term variance effective population size, N_eV, as a measure of effective population size	Decrease	Increased or remained stable depending on estimator (582 to ∞ using MLNE, 43 to 328 using estimator of moments)
