# Supplementary material for: Quantifying connectivity between local Plasmodium falciparum malaria parasite populations using identity by descent
Source: PLoS Genet. 2017 Oct 27;13(10):e1007065. doi: 10.1371/journal.pgen.1007065 (PMC5678785; doi:10.1371/journal.pgen.1007065)
Supplement: S2 Table — Clinic code: MLA (Maela), WPA (Wang Pha), MKK (Mae Kon Ken) and MKT (Mawker Thai). (PDF) [file pgen.1007065.s002.pdf]

Clinic	2001	2002	2003	2004	2005	2006	2007	2008	2009	2010	Total
MLA	17	39	35	28	2	0	5	37	26	23	212
WPA	0	0	0	12	0	0	20	228	120	77	457
MKK	0	0	0	0	0	0	4	57	40	15	116
MKT	12	58	67	22	19	12	3	88	70	37	388
Total	29	97	102	62	21	12	32	410	256	152	1173
