# Supplementary material for: Quantifying connectivity between local Plasmodium falciparum malaria parasite populations using identity by descent
Source: PLoS Genet. 2017 Oct 27;13(10):e1007065. doi: 10.1371/journal.pgen.1007065 (PMC5678785; doi:10.1371/journal.pgen.1007065)
Supplement: S3 Table — Clinic code: MLA (Maela), WPA (Wang Pha), MKK (Mae Kon Ken) and MKT (Mawker Thai). (PDF) [file pgen.1007065.s003.pdf]

Clinic	2001-2004	2008	2011-2012	2014	Total
MLA	33	12	2	8	55
WPA	3	36	48	16	103
MKK	0	0	0	4	4
MKT	0	0	0	16	16
Total	36	48	50	44	178
