# Supplementary material for: Quantifying connectivity between local Plasmodium falciparum malaria parasite populations using identity by descent
Source: PLoS Genet. 2017 Oct 27;13(10):e1007065. doi: 10.1371/journal.pgen.1007065 (PMC5678785; doi:10.1371/journal.pgen.1007065)
Supplement: S4 Table — The simple linear model was as follows. Non-reference allele frequency = intercept + βclinic clinic + βyear year + ε, where ε was normally distributed with mean 0 and variance σ2. (PDF) [file pgen.1007065.s004.pdf]

	Before Bonferroni correction		After Bonferroni correction	
	Clinic	Year	Clinic	Year
Single-genotype barcode	11 (11.83%)	9 (9.68%)	0 (0%)	0 (0%)
WGS	2326 (6.66%)	945 (2.70%)	4.58 (1.31%)	0 (0%)
