# Supplementary material for: Quantifying connectivity between local Plasmodium falciparum malaria parasite populations using identity by descent
Source: PLoS Genet. 2017 Oct 27;13(10):e1007065. doi: 10.1371/journal.pgen.1007065 (PMC5678785; doi:10.1371/journal.pgen.1007065)
Supplement: S1 Text — (PDF) [file pgen.1007065.s005.pdf]

# S1 Text

Throughout this document, pairs of clinics are denoted by their abbreviated names (MLA for Maela, WPA for Wang Pha, MKK for Mae Kon Ken and MKT for Mawker Thai).

Table A: One-tailed Monte Carlo p-value estimates for $\hat{F}_{ST}$ based on barcode data. For a given comparison between clinics, estimates were based on 1000 random permutations of the clinic labels of the parasite samples from said clinics.

| | 2001-2010 | 2008 | 2009 | 2010 |
|---|---|---|---|---|
| MKK WPA | 0.001 | 0.001 | 0.002 | 0.156 |
| MKK MKT | 0.001 | 0.001 | 0.001 | 0.004 |
| MLA WPA | 0.001 | 0.005 | 0.053 | 0.422 |
| MKT WPA | 0.001 | 0.001 | 0.001 | 0.006 |
| MKK MLA | 0.001 | 0.001 | 0.092 | 0.189 |
| MKT MLA | 0.001 | 0.003 | 0.002 | 0.029 |

Table B: One-tailed p-values for $\hat{F}_{ST}$ based on WGS data. †With the exception of the compairison between MKK and MLA in 2014, all p-values are Monte Carlo estimates based on 1000 random permutations as described in Table A. The p-value for MKK and MLA in 2014 is exact, since there were fewer than 1000 possible permutations given $n_{\text{MKK}} = 4$ parasite samples from MKK, and $n_{\text{MLA}} = 8$ parasite samples from MLA, and so all $(n_{\text{MKK}} + n_{\text{MLA}})!/(n_{\text{MKK}}! \times n_{\text{MLA}}!) = 495$ possible permutations were enumerated.

| | 2001-2014 | 2014 |
|---|---|---|
| MKK WPA | 0.001 | 0.096 |
| MKK MKT | 0.080 | 0.070 |
| MLA WPA | 0.001 | 0.006 |
| MKT WPA | 0.001 | 0.012 |
| MKK MLA | 0.001 | 0.004† |
| MKT MLA | 0.001 | 0.117 |

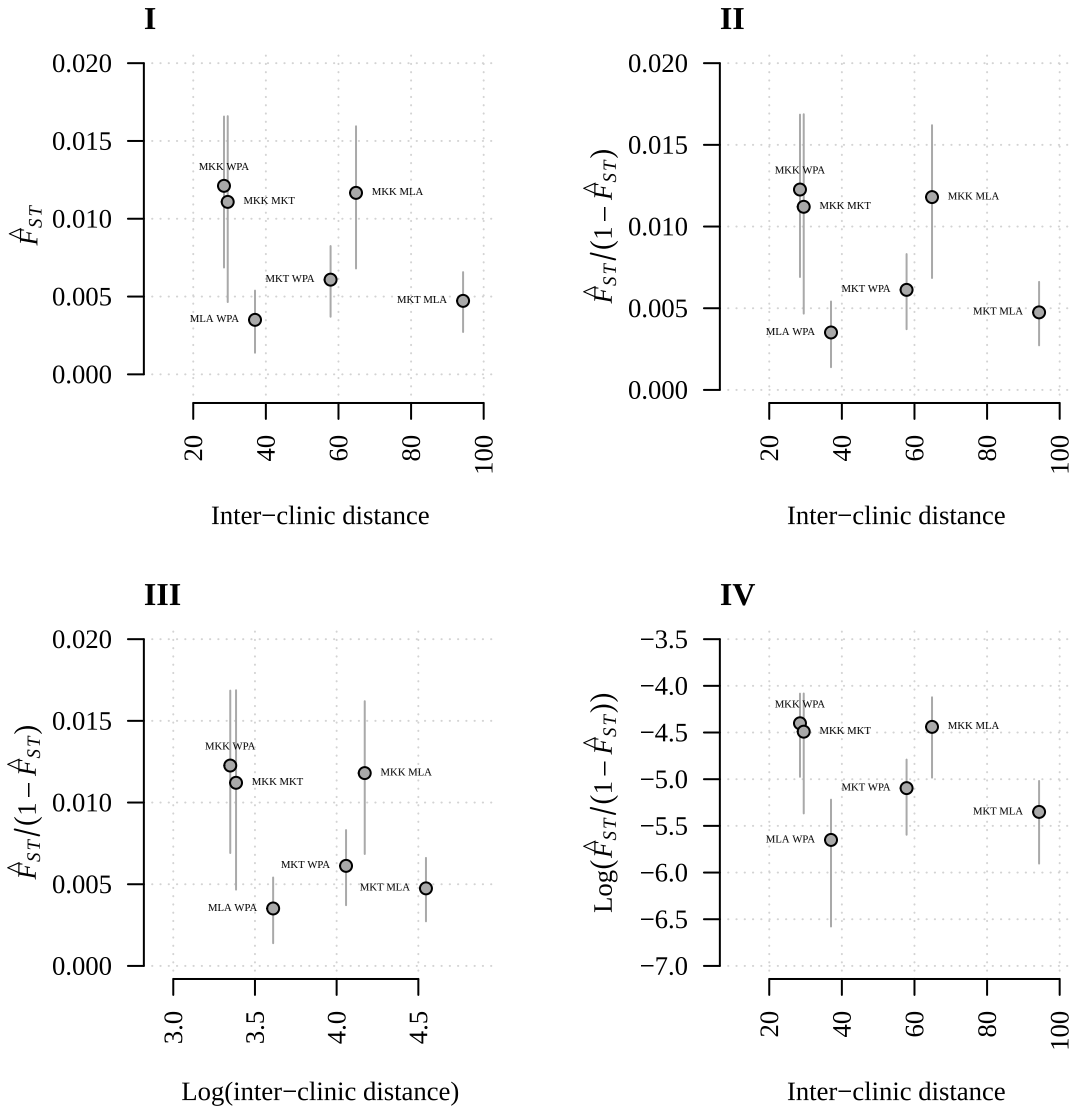


Figure A: Estimates of $F_{ST}$ based on 2001-2010 barcode data against inter-clinic distance (km). The transformations in plots I and II are predicted to be linearly related under one and two-dimensional models of isolation by distance, respectively (Rousset 1997). The logistic transformation (plot IV) was added for comparison with logit-transformed estimates based on IBD in Figure 4 of the main text. Error bars represent 95% confidence intervals based on bootstrapping SNPs over 1000 replicates.

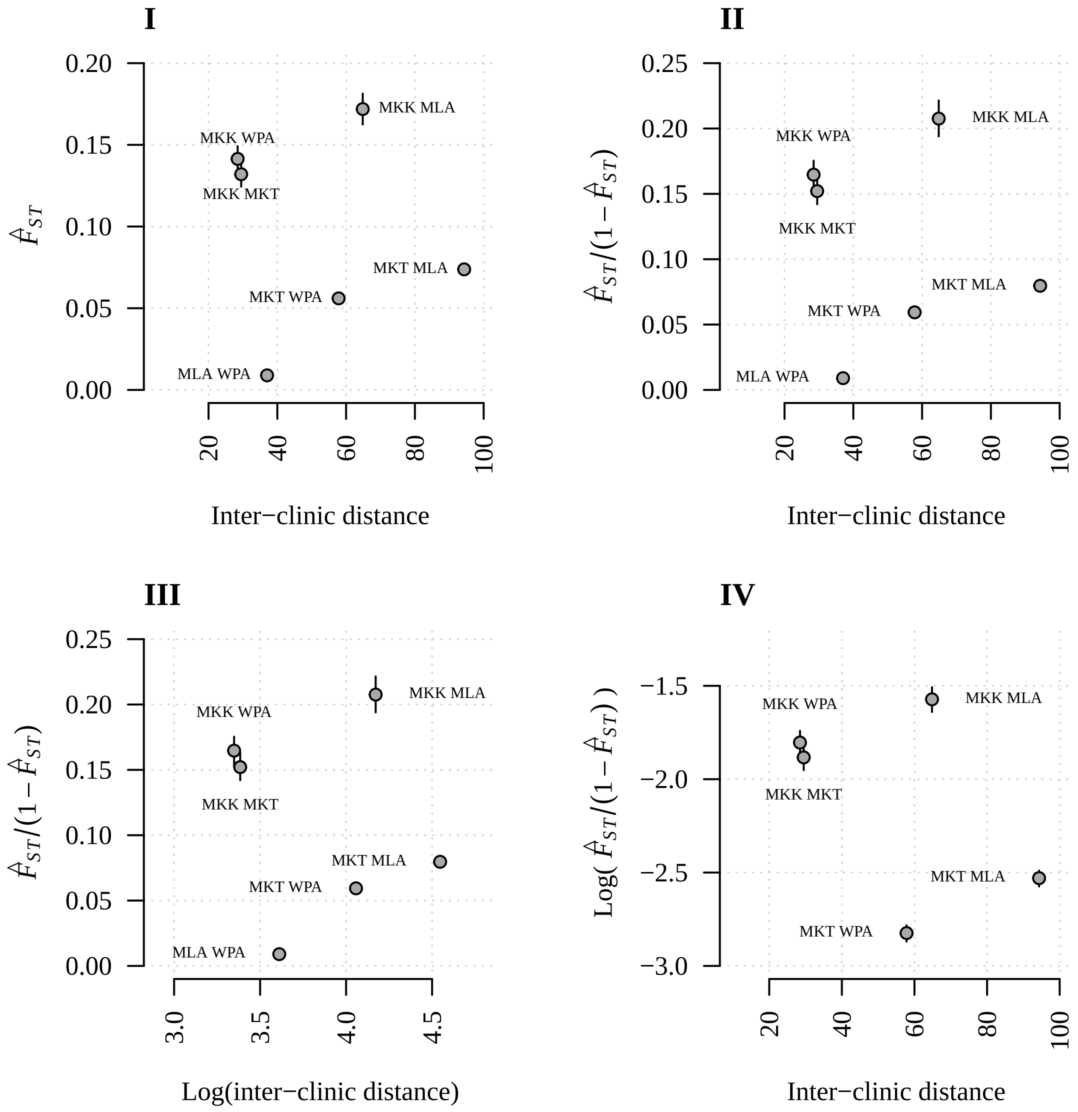


Figure B: Estimates of $F_{ST}$ based on 2001-2014 WGS data against inter-clinic distance (km). The transformations in plots II and III are predicted to be linearly related under one and two-dimensional models of isolation by distance, respectively (Rousset 1997). The logistic transformation (plot IV) was added for comparison with logit-transformed estimates based on IBD in Figure 5 of the main text. Error bars represent 95% confidence intervals based on bootstrapping SNPs over 1000 replicates.

# Understanding why $F_{ST}$ estimates based on WGS data are an order of magnitude larger than those based on barcode data

## $F_{ST}$ estimates and minor allele frequency thresholds

Some multilocus $F_{ST}$ estimators are sensitive to minor allele frequencies (MAFs), especially when marker ascertainment is not based an an out group and when estimators average over many ratios, each based on a different locus (Bhatia et al. 2013). We estimated multilocus $F_{ST}$ using Hudson's estimator, which takes a ratio of averages, rather than an average of ratios, and so should be comparatively robust to marker ascertainment (Bhatia et al. 2013). Indeed, Fig C shows that WGS-based $F_{ST}$ estimates generated using different MAF thresholds (that is to say, including only those SNPs with frequency estimates greater than the stated MAF threshold) were relatively stable.

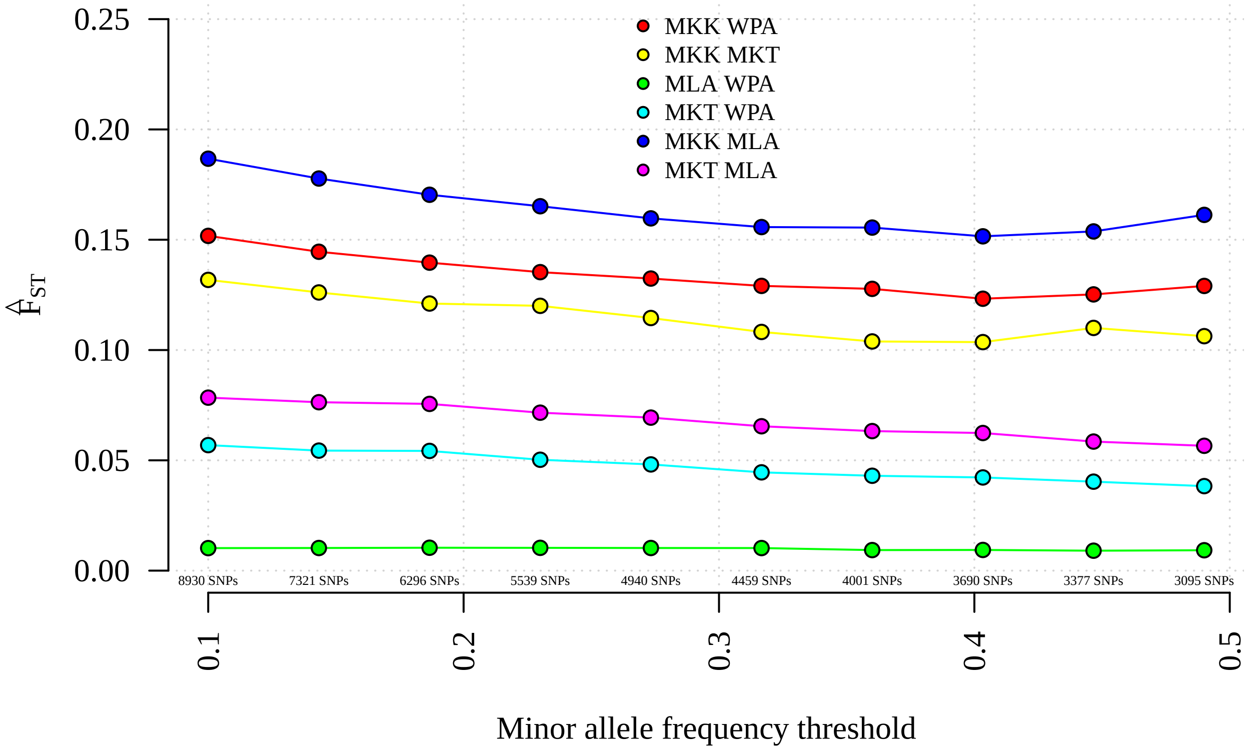


Figure C: Estimates of $F_{ST}$ based on 2001-2014 WGS data given different minor allele frequency thresholds.

## $F_{ST}$ estimates, sample sizes and within-clinic relatedness estimates

While exploring the differences between $F_{ST}$ estimates based on barcode and WGS data, we noticed an association between estimates and sample sizes (Fig D), which was surprising given that the Hudson estimator is recommended for small and unequal sample sizes (Willing, Dreyer, and Oosterhout 2012; Bhatia et al. 2013). In an attempt to remove potential confounding due to unequal sample sizes across clinics, we down-sampled both barcode and WGS data to their respective minimum per-clinic sample size. Specifically, from each clinic we drew 100 random subsets of 116 barcode parasite samples (the minimum per-clinic sample size for the barcode data), and 100 random subsets of 4 WGS parasite samples (the minimum per-clinic sample size for the WGS data), then re-estimated $F_{ST}$ for each random subset. Estimates based on down-sampled barcode data were robust, and most fell within the 95% confidence intervals of the estimates based on the non-downsampled barcode data (Fig E). $F_{ST}$ estimates based on down-sampled WGS data were not robust, and most fell outside the 95% confidence intervals of the estimates based on the non-downsampled WGS data (Fig E). We initially concluded, therefore, that despite using a estimator recommended for small and unequal sample sizes (Willing, Dreyer, and Oosterhout 2012; Bhatia et al. 2013), we have too few WGS parasite samples to inform meaningful $F_{ST}$ estimates based on WGS data.

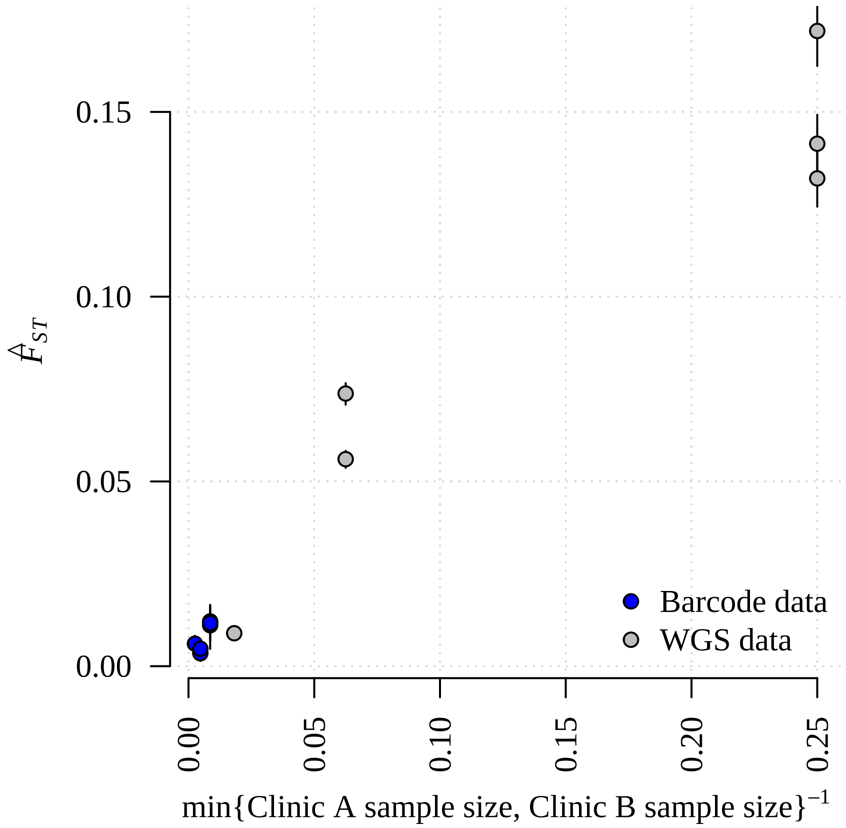


Figure D: $F_{ST}$ estimates plotted against the inverse of the corresponding minority per-clinic sample size, 1/min{Clinic A sample size, Clinic B sample size}.

However, upon further investigation as to why results based on *P. falciparum* data from the Thai-Myanmar border might deviate from those reported in the literature, we noticed a decline in the proportion of highly related parasite sample pairs (equation (1)) with sample sizes across clinics (Fig F), which in turn was associated with $\hat{F}_{ST}$.

$$\hat{R} = \frac{1}{N} \sum_{N} \mathbb{I}\big(\hat{\pi}_{\text{IBD}} > 0.5\big), \tag{1}$$

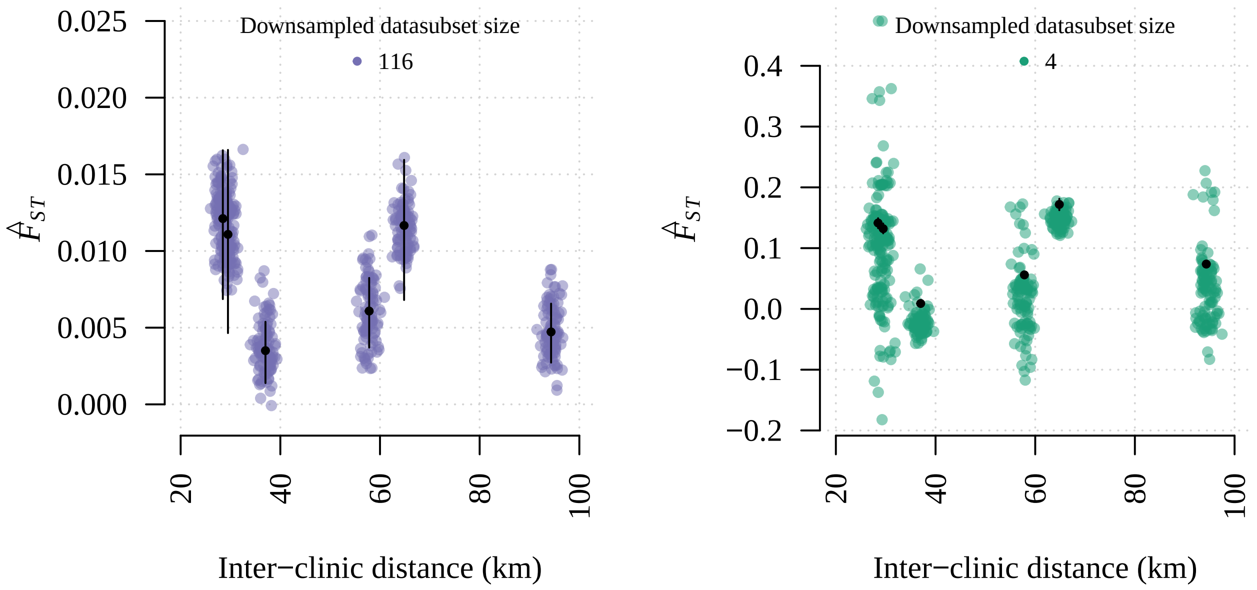


Figure E: Estimates of $F_{ST}$ based on full and downsampled data. Plots on the left and right show estimates based on barcode and WGS data, respectively. Black points and error bars show estimates generated using non-downsampled barcode and WGS data and their 95% confidence intervals, which were generated by booting SNPs over 1000 replicates. Coloured points represent $F_{ST}$ estimates based on randomly drawn downsampled data.

where $N$ is the number of parasite sample pairs, $\hat{\pi}_{\text{IBD}}$ is defined in the main text and $\mathbb{I}$ is the indicator function. More specifically, $\hat{F}_{ST}$ increased with dominant within-clinic $\hat{R}$ (Fig G), where for clinics A and B say,

$$\text{dominant within-clinic } \hat{R} = \max\{\text{within-clinic A } \hat{R},\ \text{within-clinic B } \hat{R}\}. \tag{2}$$

WGS inter-clinic $\hat{R}$ were also associated with dominant within-clinic $\hat{R}$ (right-hand plot Fig H), although not as markedly as $\hat{F}_{ST}$. $F_{ST}$ estimates were not strongly associated with inter-clinic $\hat{R}$ (Fig I).

These observations were based on very few point estimates. Nevertheless, an association between $F_{ST}$ and dominant within-clinic $\hat{R}$ is not unexpected given the definition $F_{ST}$, which can be expressed as a normalized measure of genetic variation between populations (Nei 1973). We therefore conclude that the capacity to detect spatial trends in the data from the Thai-Myanmar border using $\hat{F}_{ST}$ is potentially overwhelmed by within-clinic relatedness, which increased with decreasing WGS per-clinic sample size, likely due to decreased transmission (Nkhoma et al. 2013; Carrara et al. 2013), rendering WGS-based $\hat{F}_{ST}$ unstable (Fig E).

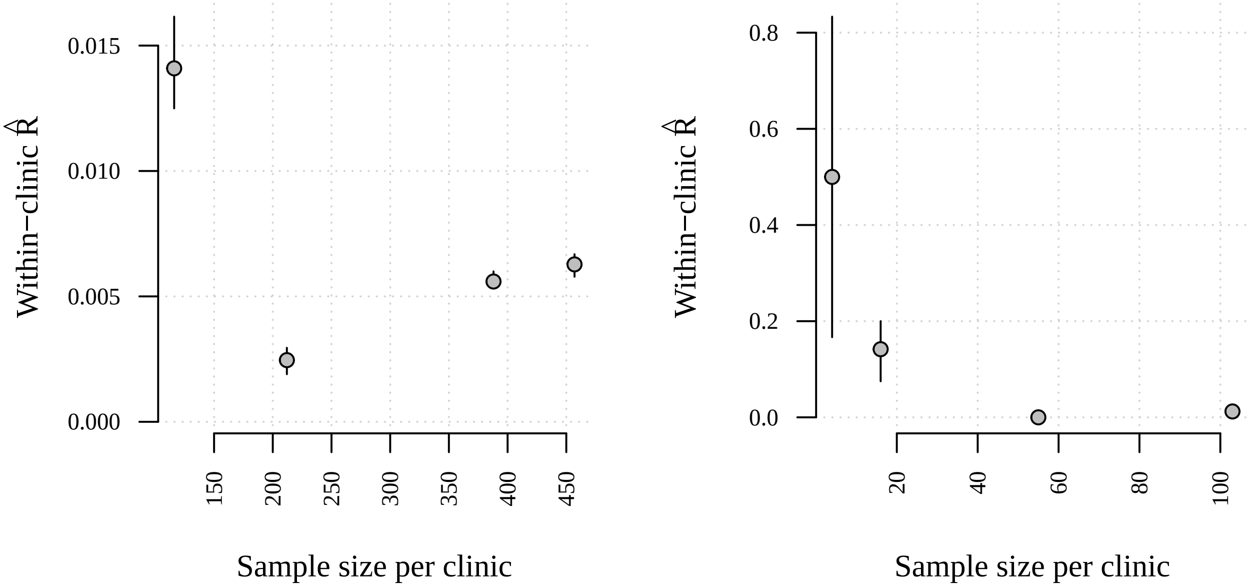


Figure F: Within-clinic $\hat{R}$ against per-clinic sample size. The plot on the left is based on barcode data. The plot on the right is based on WGS data.

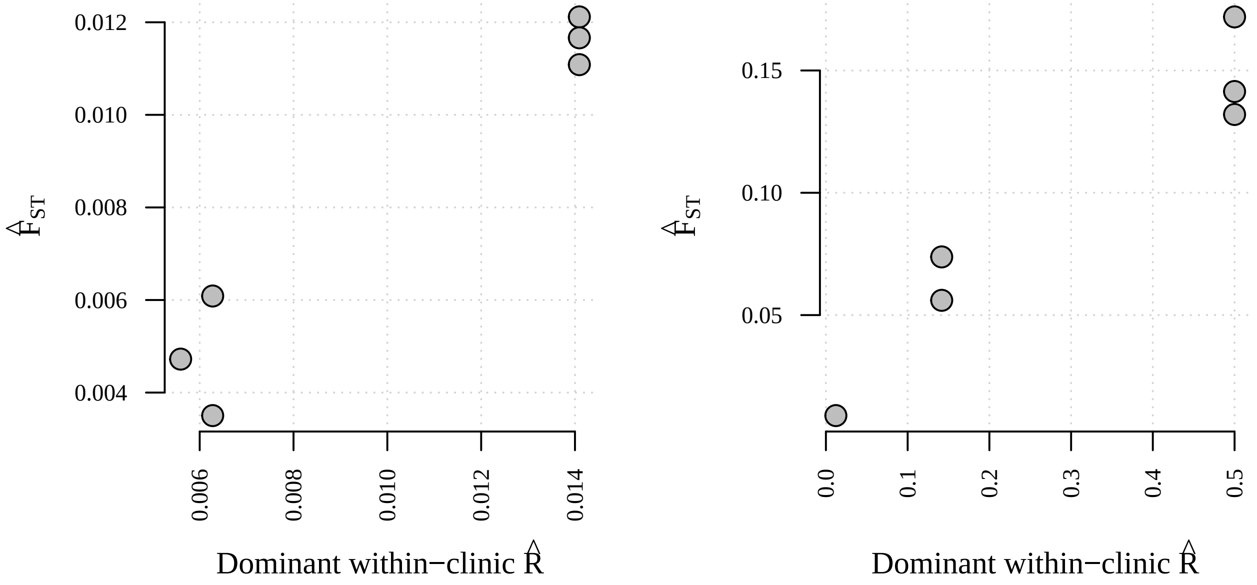


Figure G: $\hat{F}_{ST}$ against dominant within-clinic $\hat{R}$. The plot on the left is based on barcode data. The plot on the right is based on WGS data.

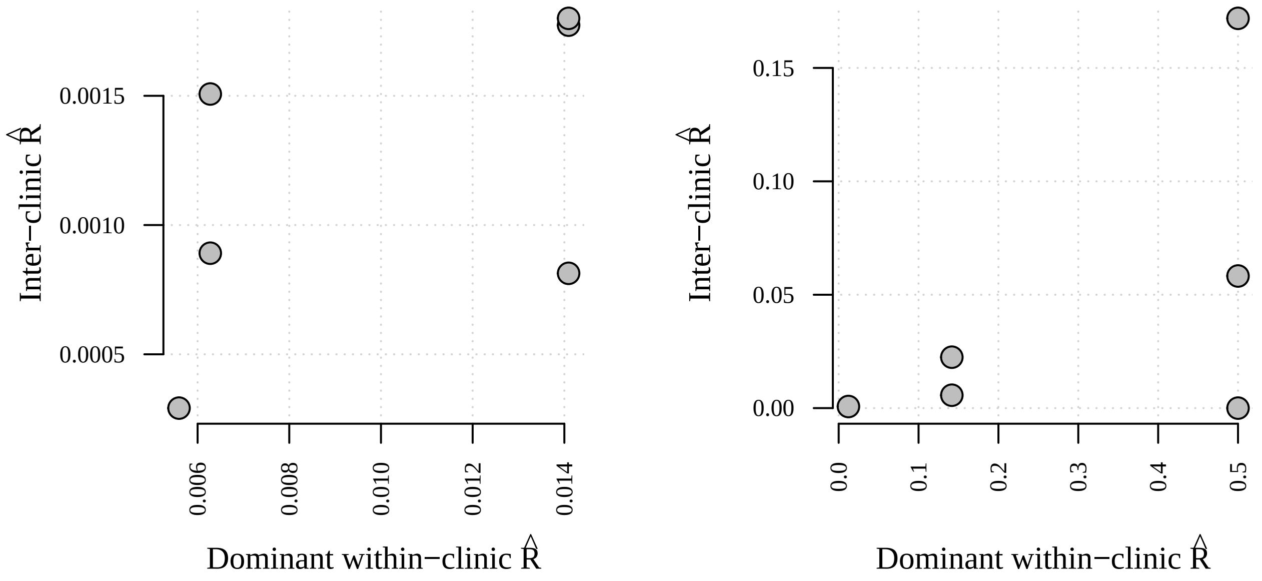


Figure H: Inter-clinic $\hat{R}$ against dominant within-clinic $\hat{R}$. The plot on the left is based on barcode data. The plot on the right is based on WGS data.

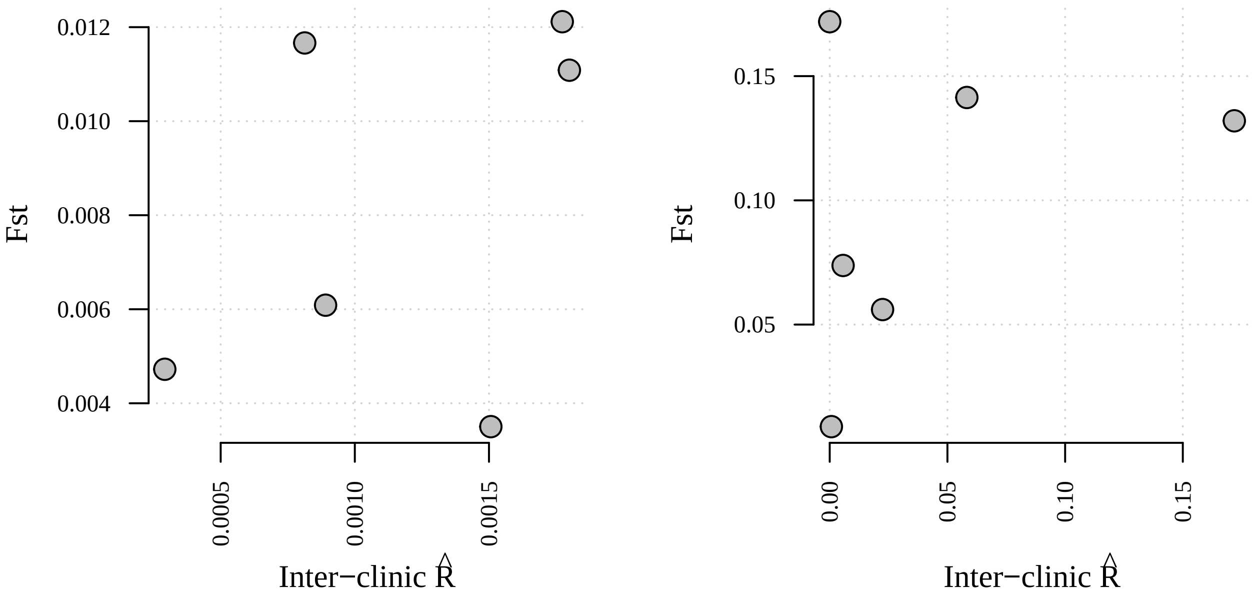


Figure I: $\hat{F}_{ST}$ and inter-clinic $\hat{R}$. The plot on the left is based on barcode data. The plot on the right is based on WGS data.

# References

Bhatia, Gaurav, Nick Patterson, Sriram Sankararaman, and Alkes L Price. 2013. “Estimating and interpreting F ST: The impact of rare variants.” *Genome Research* 23 (9): 1514–21.

Carrara, Verena I., Khin Maung Lwin, Aung Pyae Phyo, Elizabeth Ashley, Jacher Wiladphaingern, Kanlaya Sriprawat, Marcus Rijken, et al. 2013. “Malaria Burden and Artemisinin Resistance in the Mobile and Migrant Population on the Thai-Myanmar Border, 1999-2011: An Observational Study.” *PLoS Medicine* 10 (3): 1999–2011.

Nei, Masatoshi. 1973. “Analysis of Gene Diversity in Subdivided Populations.” *Proceedings of the National Academy of Sciences* 70 (12): 3321–3.

Nkhoma, Standwell C., Shalini Nair, Salma Al-Saai, Elizabeth Ashley, Rose McGready, Aung P. Phyo, Francois Nosten, and Tim J C Anderson. 2013. “Population genetic correlates of declining transmission in a human pathogen.” *Molecular Ecology* 22 (2): 273–85.

Willing, Eva Maria, Christine Dreyer, and Cock van Oosterhout. 2012. “Estimates of genetic differentiation measured by fst do not necessarily require large sample sizes when using many snp markers.” *PLoS ONE* 7 (8): e42649.
