# Supplementary material for: Quantifying connectivity between local Plasmodium falciparum malaria parasite populations using identity by descent
Source: PLoS Genet. 2017 Oct 27;13(10):e1007065. doi: 10.1371/journal.pgen.1007065 (PMC5678785; doi:10.1371/journal.pgen.1007065)
Supplement: S2 Text — (PDF) [file pgen.1007065.s006.pdf]

S2 Text

Trends in relatedness

In this section we briefly describe preliminary analyses on which the structures of the temporally unadjusted and adjusted logistic regression models of highly related parasite sample pairs were based. The analyses were performed using barcode data since they were most comprehensively sampled.

Motivation for regressing highly related parasite sample pairs onto distance

Empirical density plots (Fig A to J) suggest that $\hat{\pi}_{\text{IBD}}$ for pairs of parasite samples within and across clinics loosely follow a mixture distribution over three classes: 1) near zero $\hat{\pi}_{\text{IBD}}$ shown in green; 2) low to intermediate $\hat{\pi}_{\text{IBD}}$ shown in shades of blue; and 3) high $\hat{\pi}_{\text{IBD}}$, which notably rarefies with distance, and is colored red if sufficiently dense to be visible, and otherwise outlined in black.

We interpreted near zero $\hat{\pi}_{\text{IBD}}$ as comparisons between parasites whose relatives were either not sampled or where barcode SNPs were to few to resolve IBD; low to intermediate $\hat{\pi}_{\text{IBD}}$ as comparisons between panmictically mixing parasites; and high $\hat{\pi}_{\text{IBD}}$ as comparisons including recent parasite migrants between clinics.

To distinguish recent migrants, we selected a threshold equal to 0.5 (dotted vertical lines, Fig A to J), classing all comparisons above the threshold highly related. We then constructed logistic regression models, as outlined below, to quantify distance as a correlate of the probability of a comparison being classed highly related.

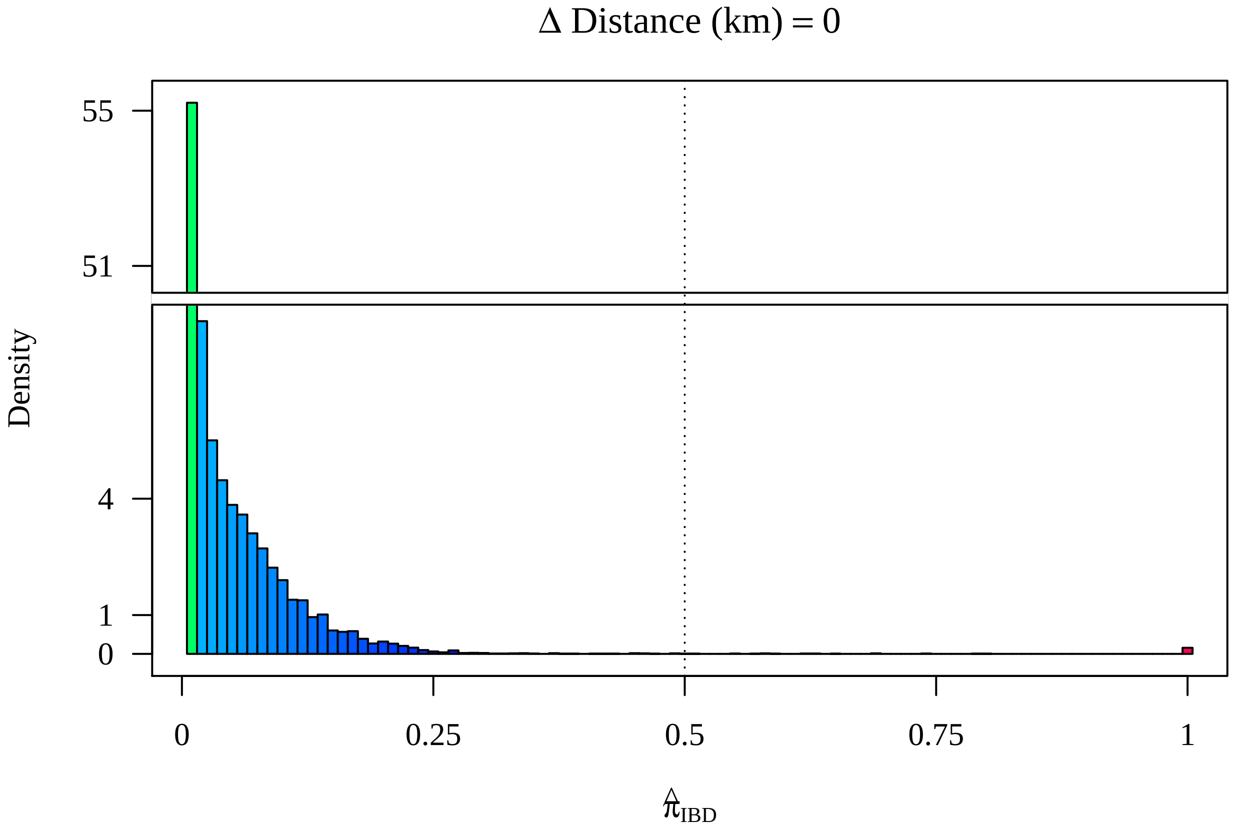

Figure A: Density of $\hat{\pi}_{\text{IBD}}$ for comparisons within Maela.

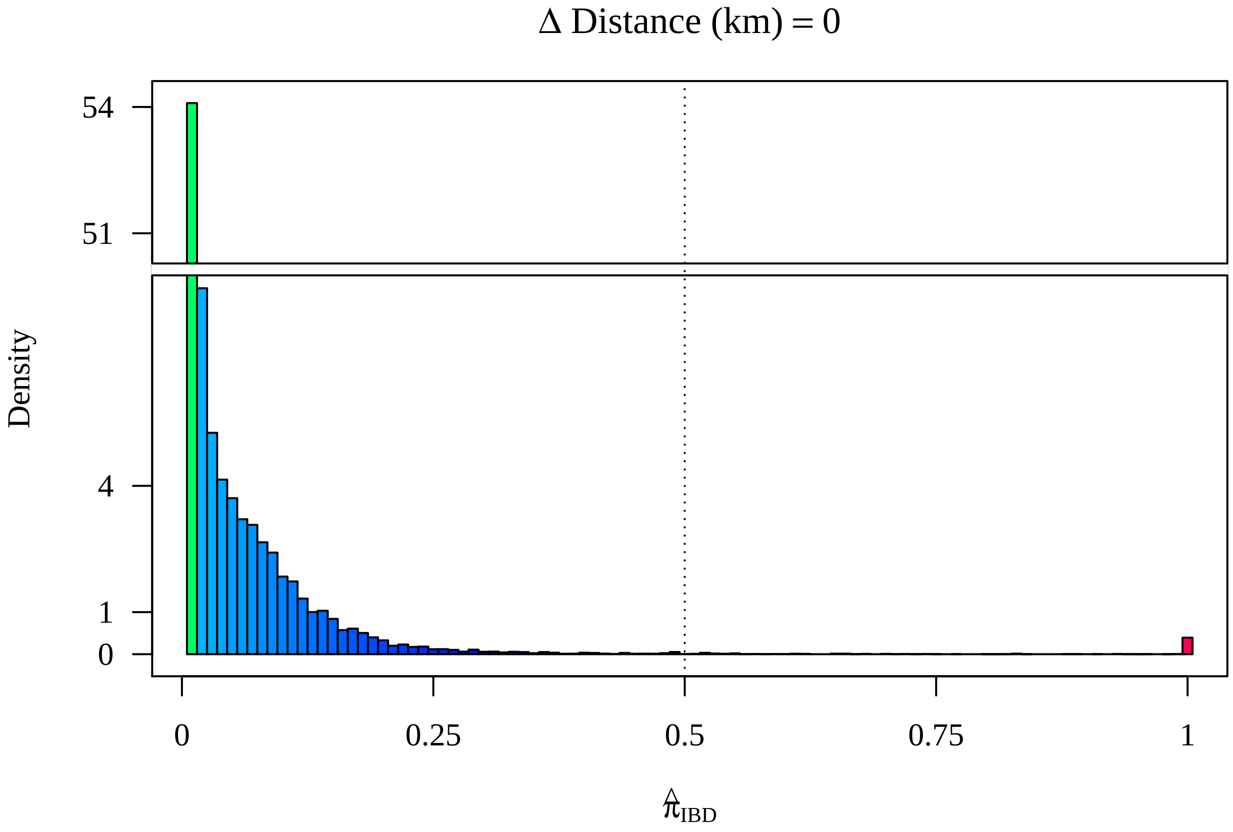

Figure B: Density of $\hat{\pi}_{\text{IBD}}$ for comparisons within Wang Pha.

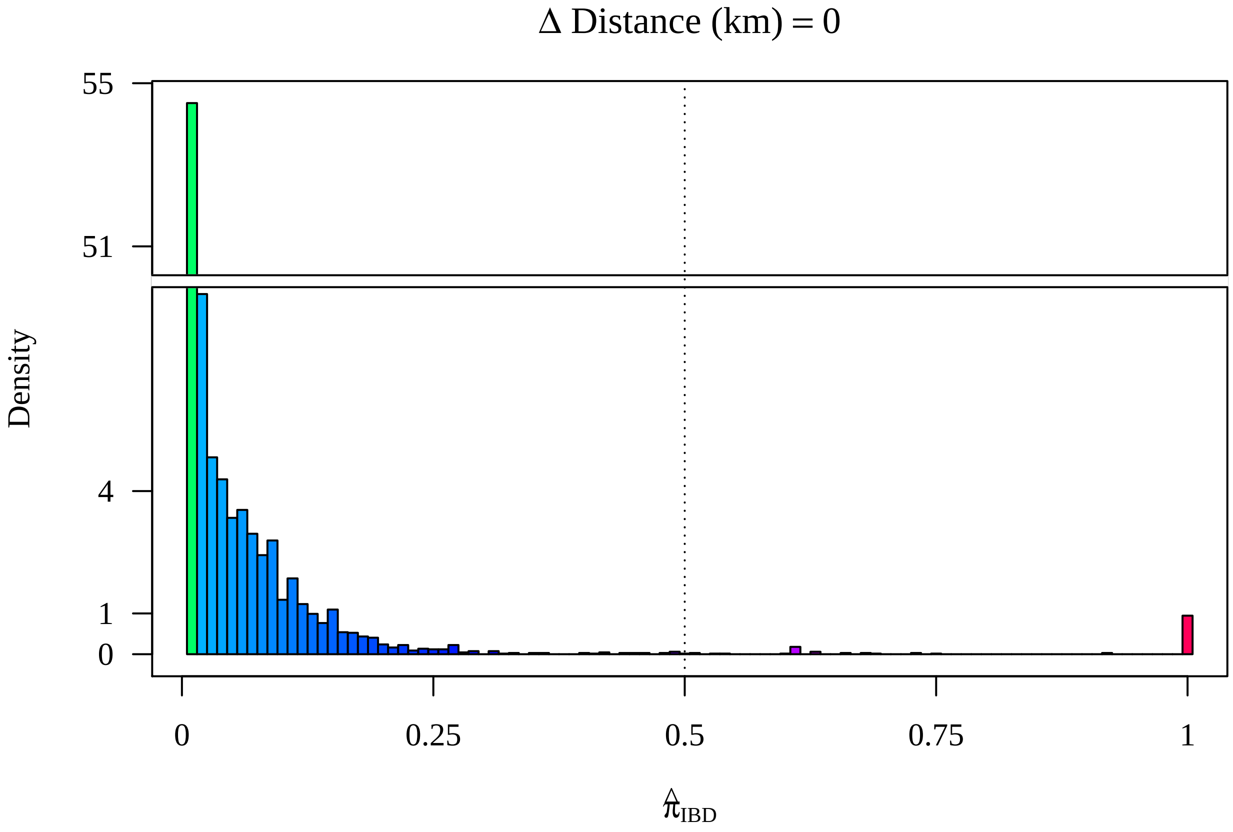

Figure C: Density of $\hat{\pi}_{\mathrm{IBD}}$ for comparisons within Mae Kon Ken.

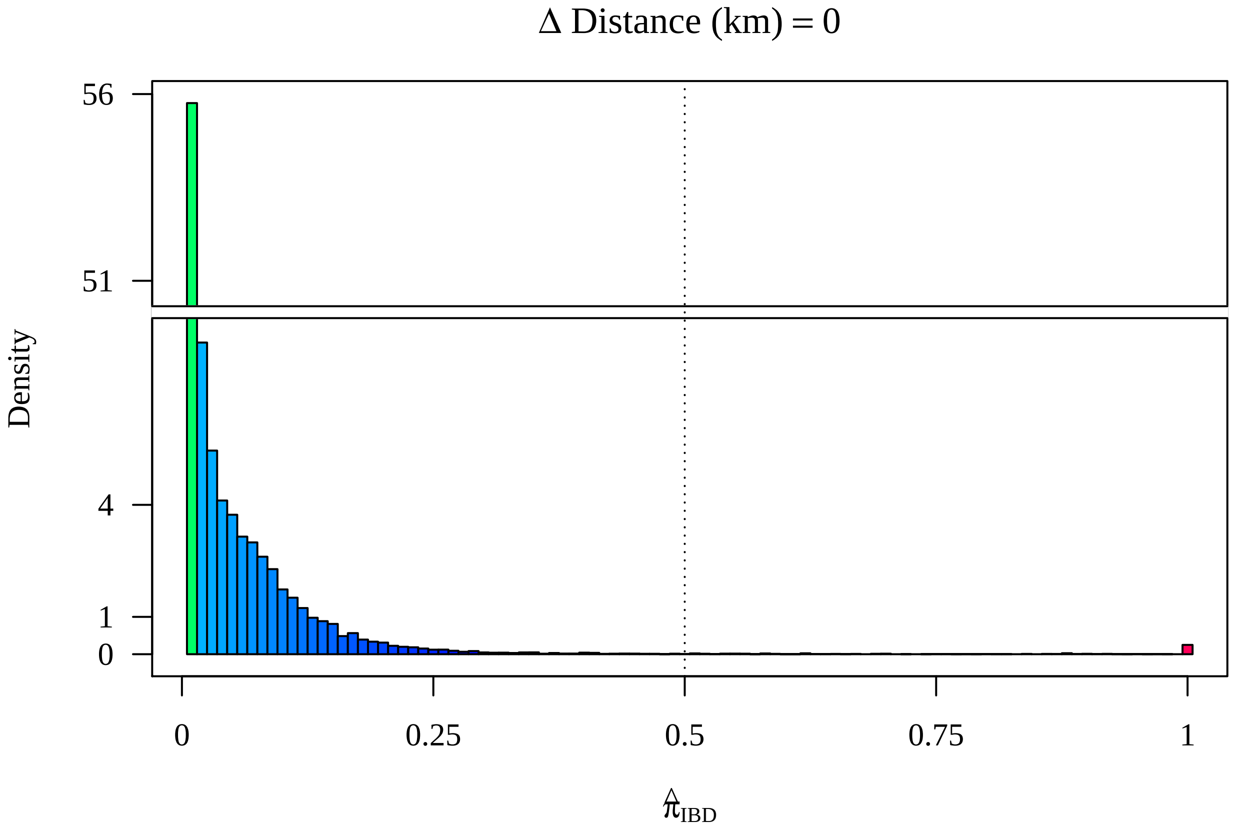

Figure D: Density of $\hat{\pi}_{\text{IBD}}$ for comparisons within Mawker Thai.

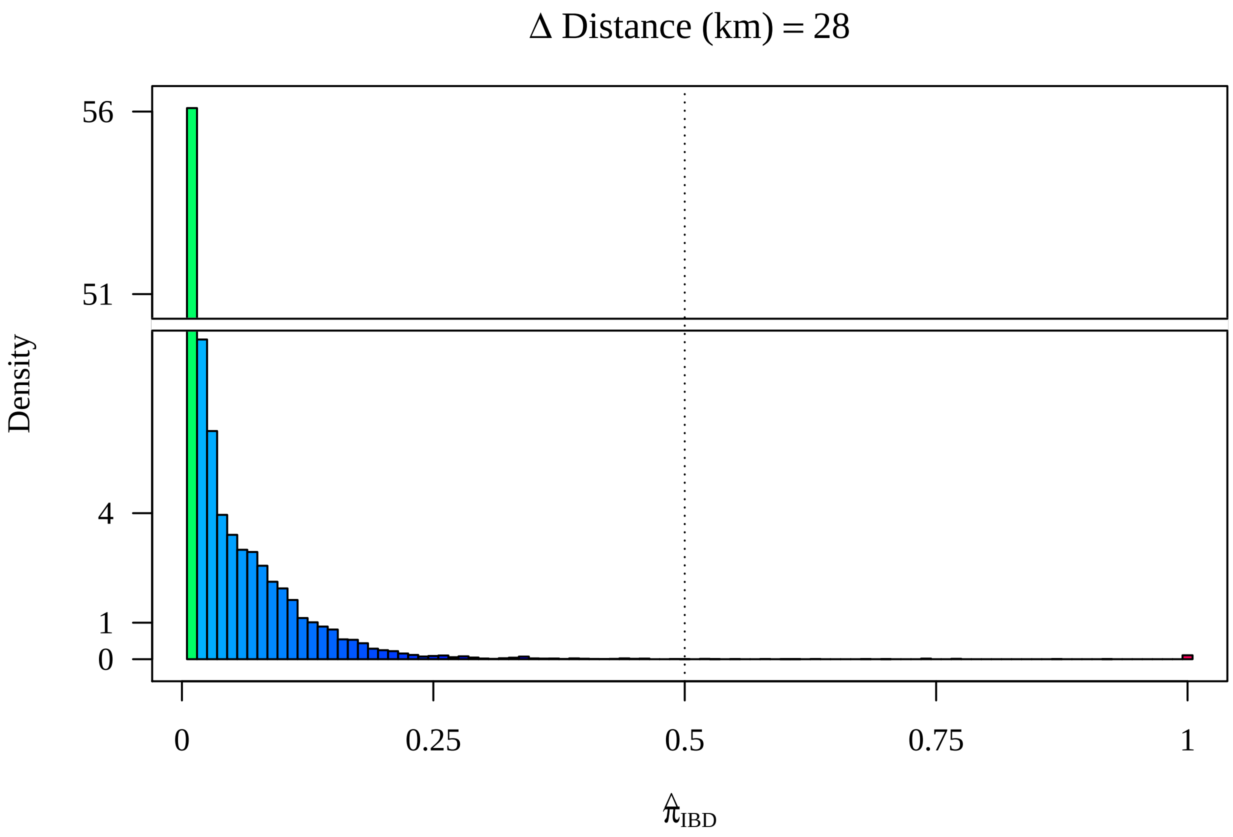

Figure E: Density of $\hat{\pi}_{\text{IBD}}$ for comparisons across Mae Kon Ken and Wang Pha.

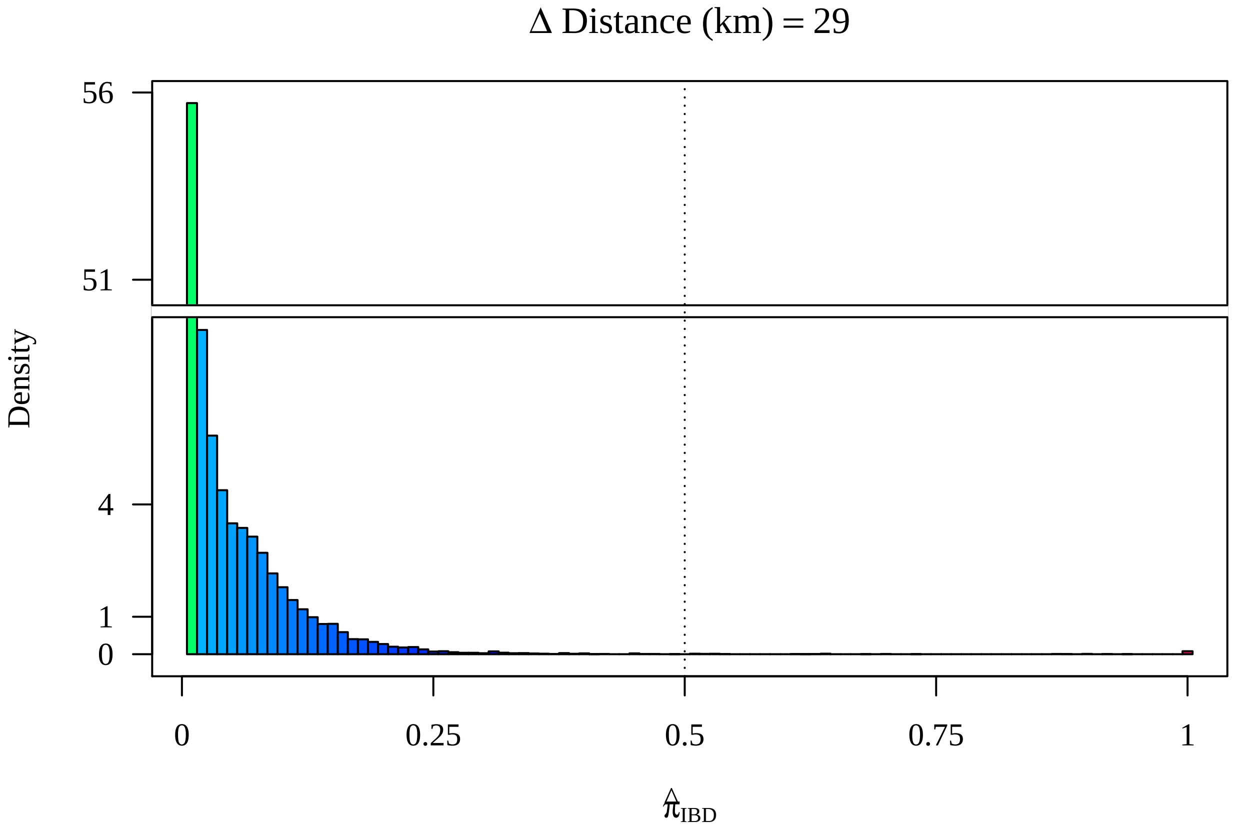

Figure F: Density of $\hat{\pi}_{\rm IBD}$ for comparisons across Mae Kon Ken and Mawker Thai.

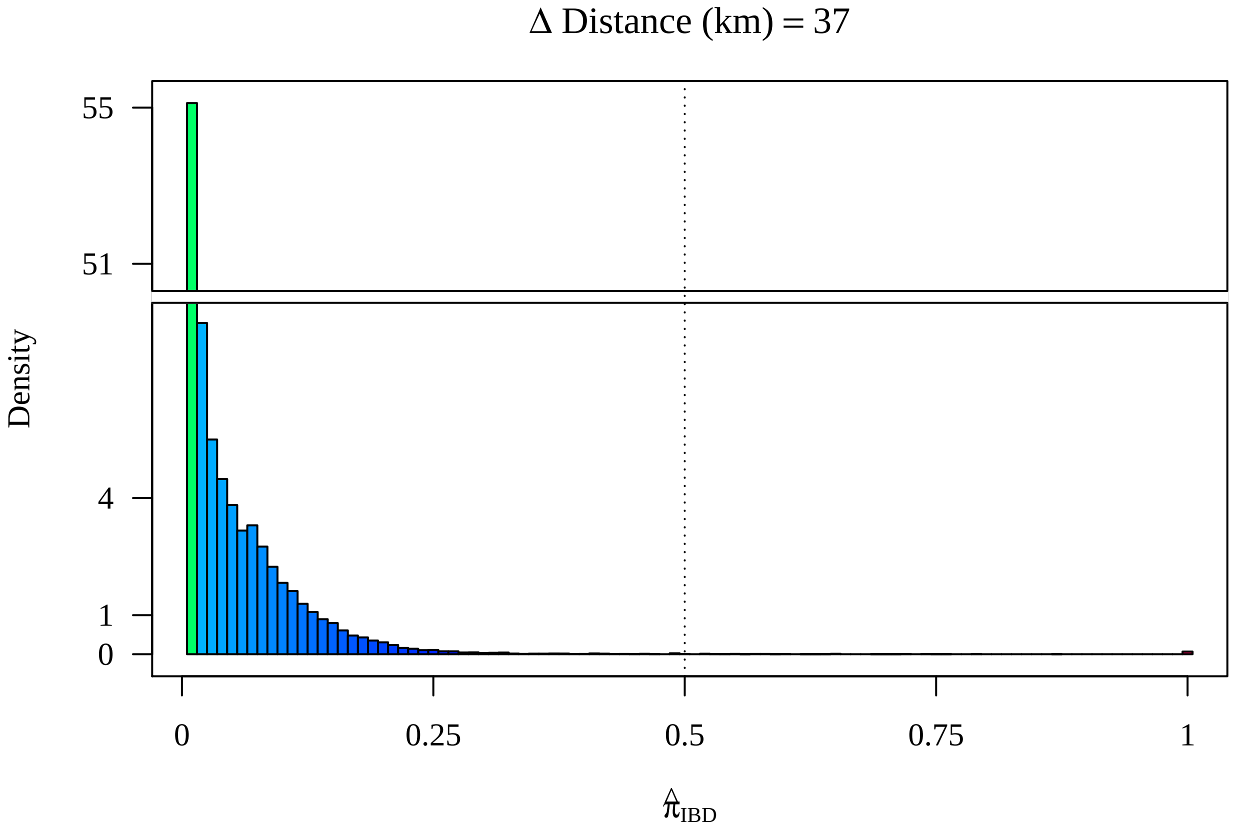

Figure G: Density of $\hat{\pi}_{\text{IBD}}$ for comparisons across Maela and Wang Pha.

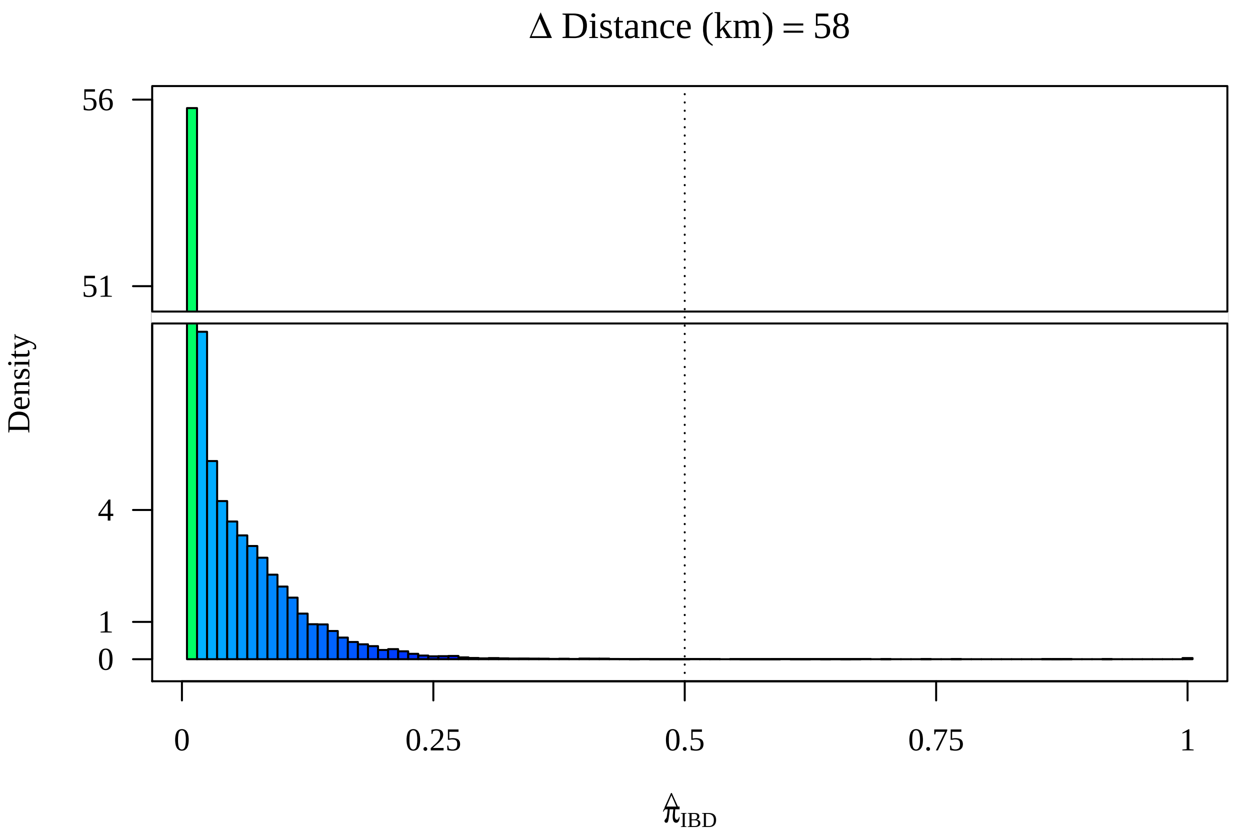

Figure H: Density of $\hat{\pi}_{\text{IBD}}$ for comparisons across Mawker Thai and Wang Pha.

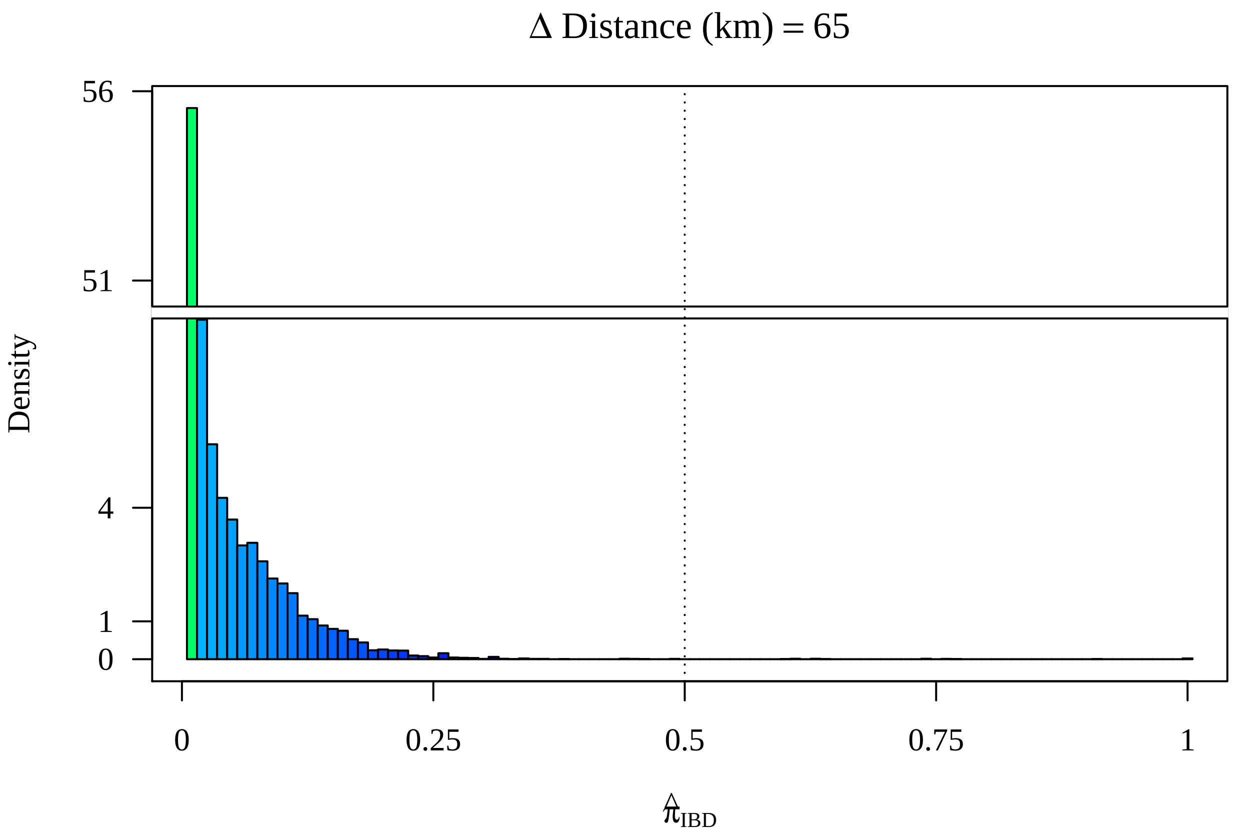

Figure I: Density of $\hat{\pi}_{\mathrm{IBD}}$ for comparisons across Maela and Mae Kon Ken.

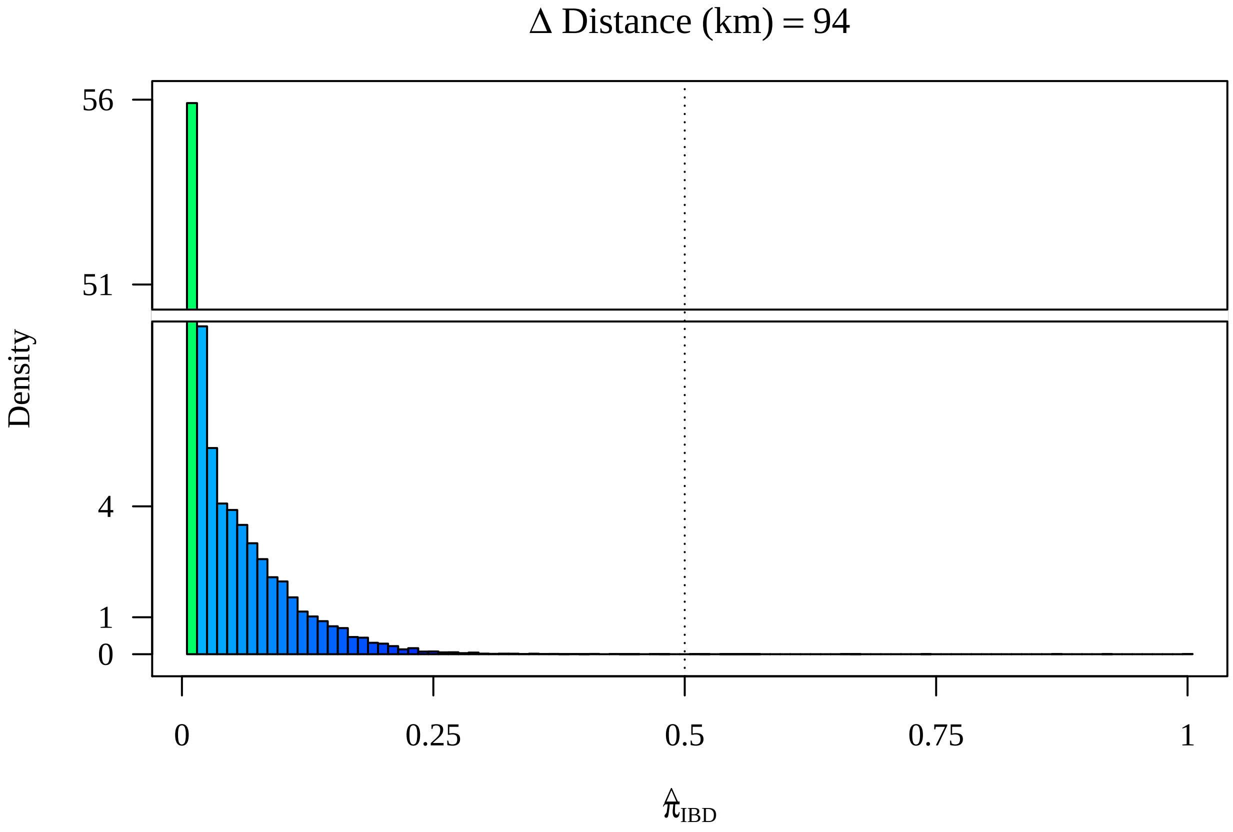

Figure J: Density of $\hat{\pi}_{\text{IBD}}$ for comparisons across Maela and Mawker Thai.

Temporally unadjusted logistic regression model

For $i = 1, \ldots, n$ parasite sample comparisons, the logistic model can be represented as a latent-data formulation as follows:

$$\mathbb{P}(\hat{\pi}_{\mathrm{IBD}_i} > 0.5) = \begin{cases} 1 \text{ if } z_i > 0, \\ 0 \text{ if } z_i < 0, \end{cases}$$

where z_i is a continuous, unobserved outcome (Gelman and Hill 2007). To quantify temporally unadjusted trends in relatedness with distance, the following two models were compared using the Akaike information criterion (AIC), a model comparison score that is penalized by model complexity and favors comparatively low values (Akaike 1998).

1) $z_i = \beta_0 + \beta_1 \Delta\text{Distance}_i + \epsilon_i$
2) $z_i = \beta_0 + \beta_1 \Delta\text{Distance}_i + \beta_2 \text{Wang Pha}_i + \beta_3 \text{Mawker Thai}_i + \beta_4 \text{Maela}_i + \beta_5 \text{Mae Kon Ken}_i + \epsilon_i,$

where ϵ_i is assumed to have a logistic probability distribution, and clinic predictors (Maela, Wang Pha, Mae Kon Ken and Mawker Thai) are equal to TRUE if both parasite samples in comparison i were collected from said clinic, and FALSE otherwise. That is to say, the clinic predictors account for inter-clinic variance at $\Delta\text{Distance}_i = 0$, but do not vary with $\Delta\text{Distance}_i > 0$. The inclusion of the clinic predictors resulted in a decrease in AIC from 2.3074×10^4 to 2.2954×10^4, while β_1 under models 1 and 2 remained comparatively stable (-0.034 and -0.026 respectively). We therefore chose model 2 to assess temporally unadjusted spatial trends.

Temporally adjusted logistic regression model

To quantify temporally adjusted trends in relatedness with distance, the following eight models with temporal predictors were compared.

3) $z_i = \ldots + \beta_6 \Delta\text{Time}_i + \epsilon_i$
4) $z_i = \ldots + \beta_6 \Delta\text{Time}_i + \beta_7 \Delta\text{Distance}_i \times \Delta\text{Time} + \epsilon_i$
5) $z_i = \ldots + \beta_6 \Delta\text{Time}_i + \beta_8 \text{Season}_i + \epsilon_i$
6) $z_i = \ldots + \beta_6 \Delta\text{Time}_i + \beta_7 \Delta\text{Distance}_i \times \Delta\text{Time}_i + \beta_8 \text{Season}_i + \epsilon_i$
5) $z_i = \ldots + \beta_6 \Delta\text{Time}_i + \beta_8 \text{Season}_i + \beta_9 \text{Season}_i \times \Delta\text{Time}_i + \epsilon_i$
8) $z_i = \ldots + \beta_6 \Delta\text{Time}_i + \beta_7 \Delta\text{Distance}_i \times \Delta\text{Time}_i + \beta_8 \text{Season}_i + \beta_9 \text{Season}_i \times \Delta\text{Time}_i + \epsilon_i$
9) $z_i = \ldots + \beta_6 \Delta\text{Time}_i + \beta_7 \Delta\text{Distance}_i \times \Delta\text{Time}_i + \beta_8 \text{Season}_i + \beta_9 \text{Season}_i \times \Delta\text{Time}_i + \beta_{10} \text{Season}_i \times \Delta\text{Distance}_i + \epsilon_i$
10) $z_i = \ldots + \beta_6 \Delta\text{Time}_i + \beta_7 \Delta\text{Distance}_i \times \Delta\text{Time}_i + \beta_8 \text{Season}_i + \beta_9 \text{Season}_i \times \Delta\text{Time}_i + \beta_{10} \text{Season}_i \times \Delta\text{Distance}_i +$
$\beta_{11} \text{Season}_i \times \Delta\text{Distance}_i \times \Delta\text{Time}_i + \epsilon_i,$

where $\ldots$ represent $\beta_0 + \beta_1 \Delta\text{Distance}_i + \beta_2 \text{Wang Pha}_i + \beta_3 \text{Mawker Thai}_i + \beta_4 \text{Maela}_i + \beta_5 \text{Mae Kon Ken}_i$ as in model 2. Fig K shows the AIC scores for the eight models with ΔTime_i measured to the nearest day, week, month or year. There was little difference between the AIC scores for models 6 and 8 (Table A). We chose the latter with ΔTime measured in weeks because the interaction term allowed the impact of ΔDistance to vary with ΔTime, and weeks generated trend estimates of the same order as inter-clinic distance measured in kilometers (Fig L). Importantly, inputs that were independent of ΔTime were relatively robust to changes in the measurement of ΔTime (Table B), and β_1 was relatively robust to changes in model structure (Fig M).

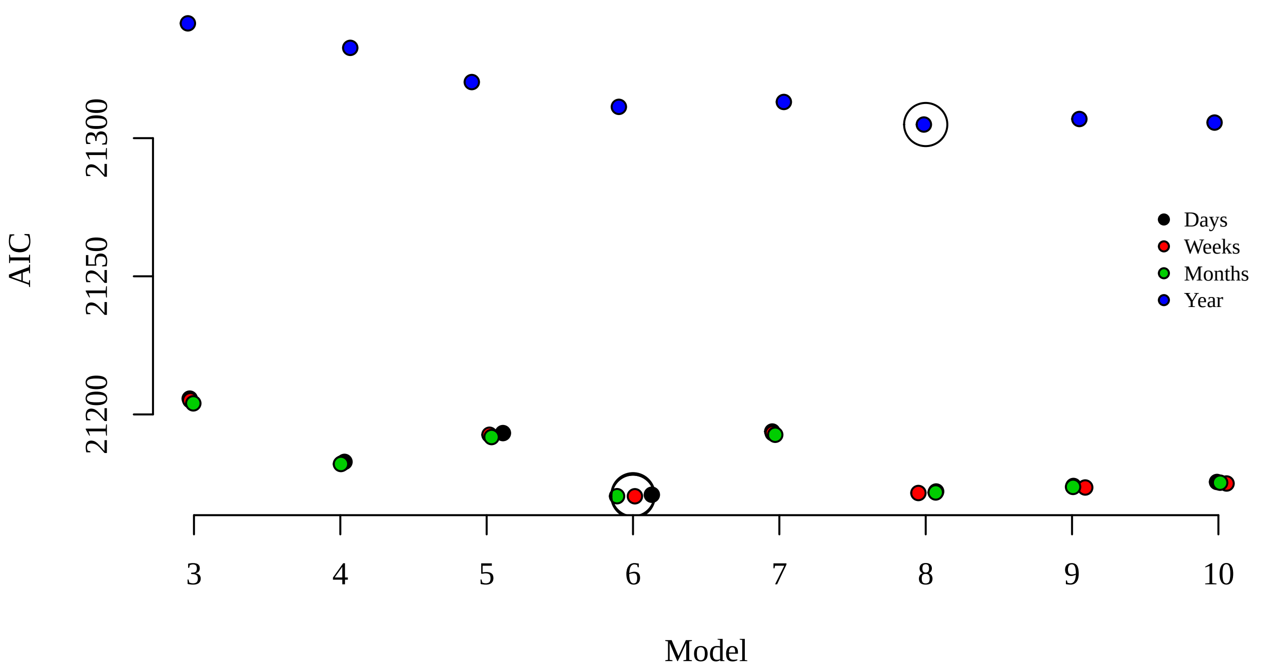

Figure K: AIC scores for models 3 to 10 fit to barcode data (the lowest score per time interval is circled).

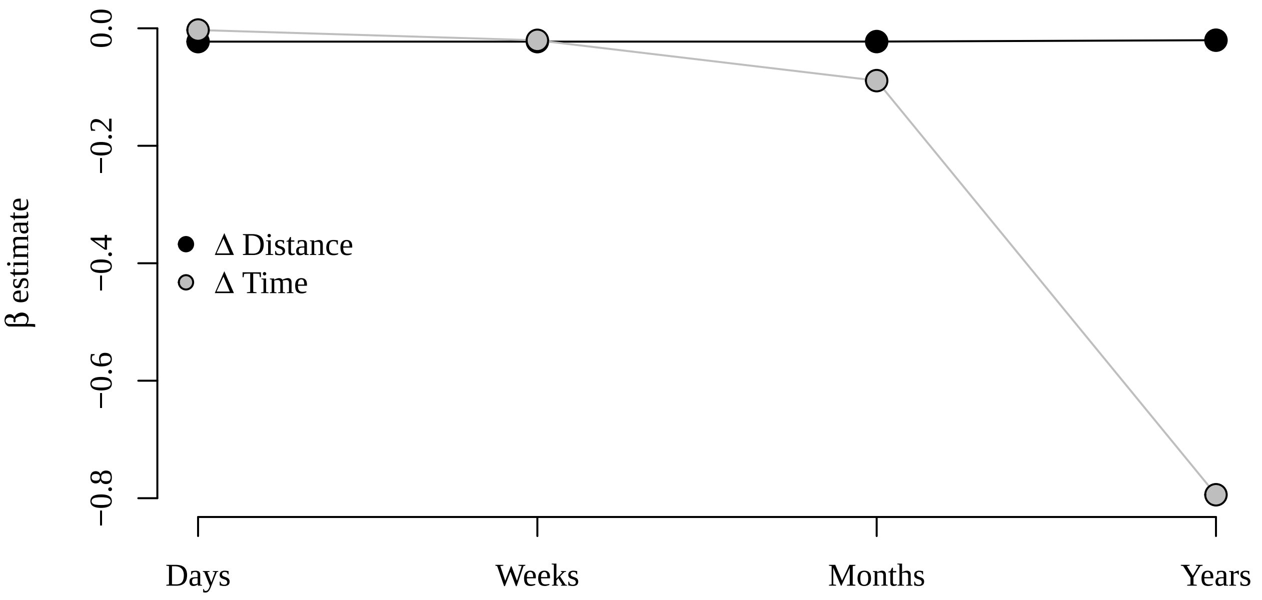

Figure L: Spatial and temporal trend estimates generated under model 8 fit to barcode data.

Table A: AIC scores of models 6 and 8 fit to barcode data with time measured in Days, Weeks, Months and Years.

	Model 6	Model 8
Days	21172	21176
Weeks	21172	21175
Months	21172	21175
Year	21305	21306

Table B: Regression coefficients estimated under model 8 fit to barcode data with time measured in Days, Weeks, Months and Years

	Days	Weeks	Months	Year
Intercept	-4.640	-4.640	-4.640	-4.927
Delta Distance (km)	-0.023	-0.023	-0.023	-0.020
Wang Pha	0.271	0.271	0.272	0.316
Mawker Thai	0.934	0.934	0.934	0.944
Maela	0.133	0.133	0.130	0.170
Mae Kon Ken	1.045	1.045	1.048	1.055
Season	0.222	0.222	0.215	0.329
Delta Time	-0.003	-0.020	-0.089	-0.794
Delta Time x Season	0.000	-0.001	-0.005	-0.173
Delta Time x Delta Distance	0.000	0.000	0.001	0.003

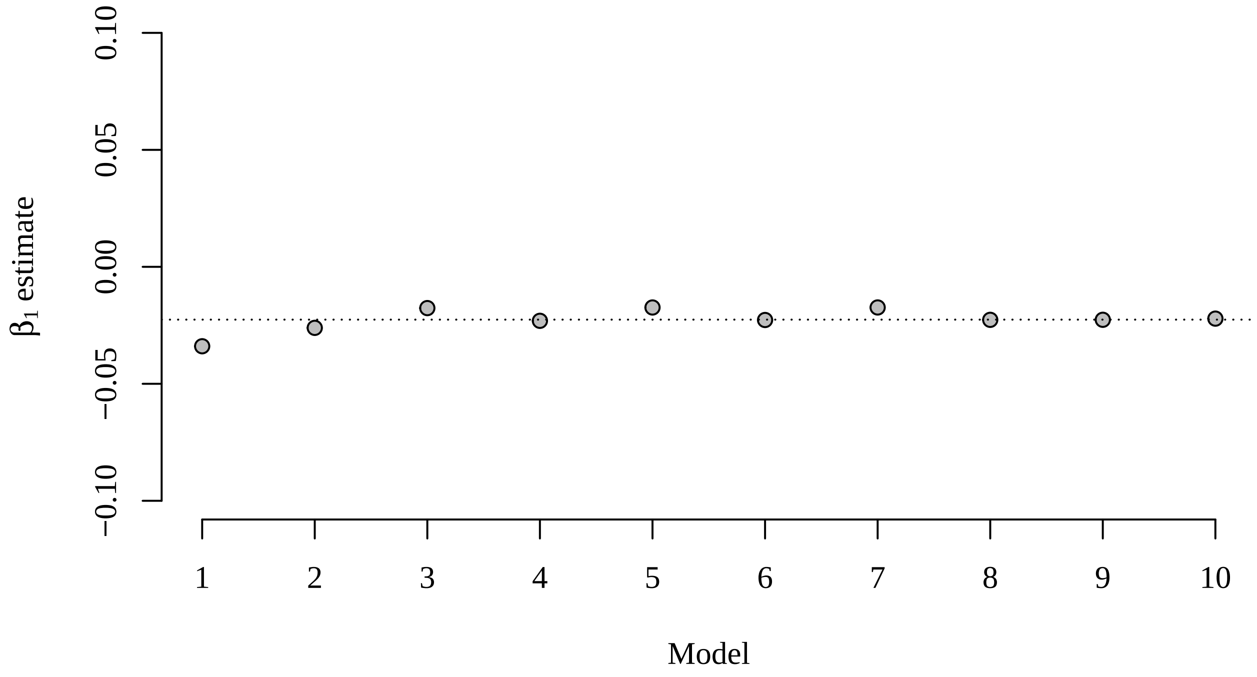

Figure M: Spatial trend estimates, $\hat{\beta}_1$ under models 1 to 10 with time measured in weeks.

Temporally adjusted model with additional year 2014 predictor for WGS data

Temporally unadjusted and adjusted models 2 and 8, respectively, were chosen using barcode data since they were the most comprehensively sampled. For comparison with results based on barcode data, we fit the same two models to WGS data, as well as an additional model,

$$11)\ z_i = \ldots + \beta_6 \Delta\text{Time}_i + \beta_7 \Delta\text{Distance}_i \times \Delta\text{Time}_i + \beta_8 \text{Season}_i + \beta_9 \text{Season}_i \times \Delta\text{Time}_i + \text{Year 2014}_i + \epsilon_i,$$

where Year 2014_i was TRUE if both parasite samples were collected in 2014 and false otherwise, thereby accounting for a large increase in $\hat{\pi}_{\text{IBD}}$ in 2014 (Fig N), and resulting in a decrease in AIC of 66 compared with model 8.

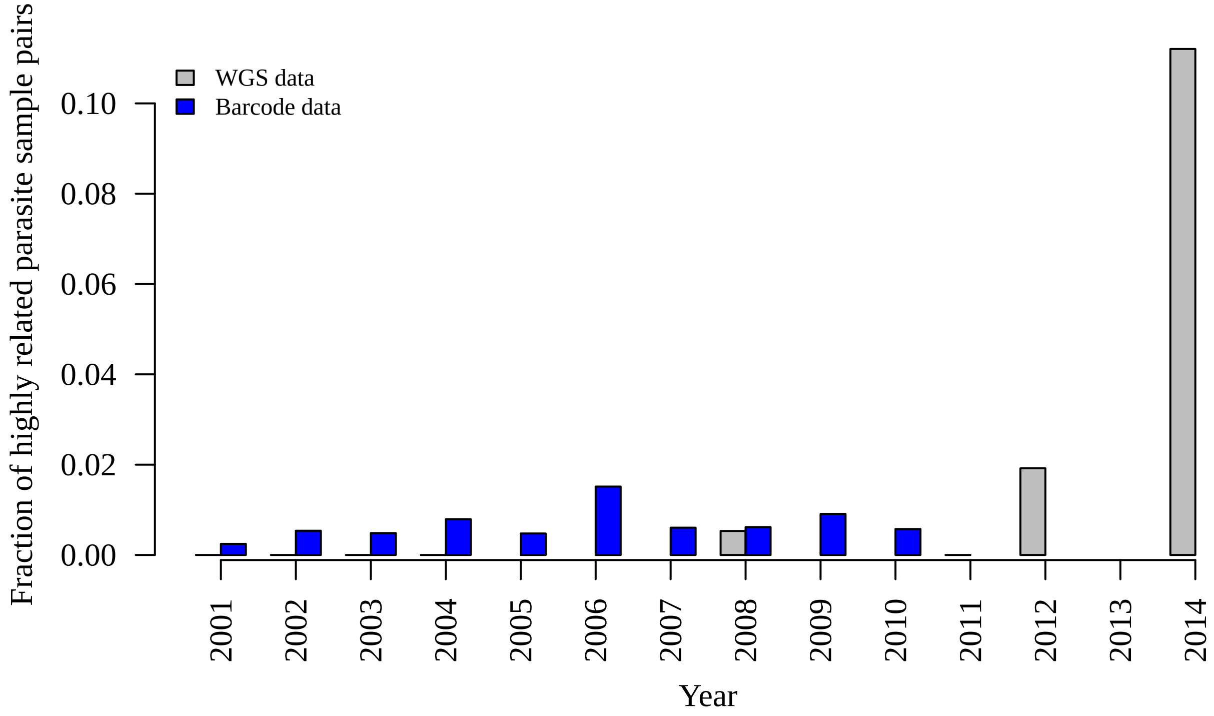

Figure N: Fraction of highly related parasite sample pairs (pairs with $\hat{\pi}_{\mathrm{IBD}} > 0.5$) over time

Proportions of highly related parasite sample pairs within and across clinics

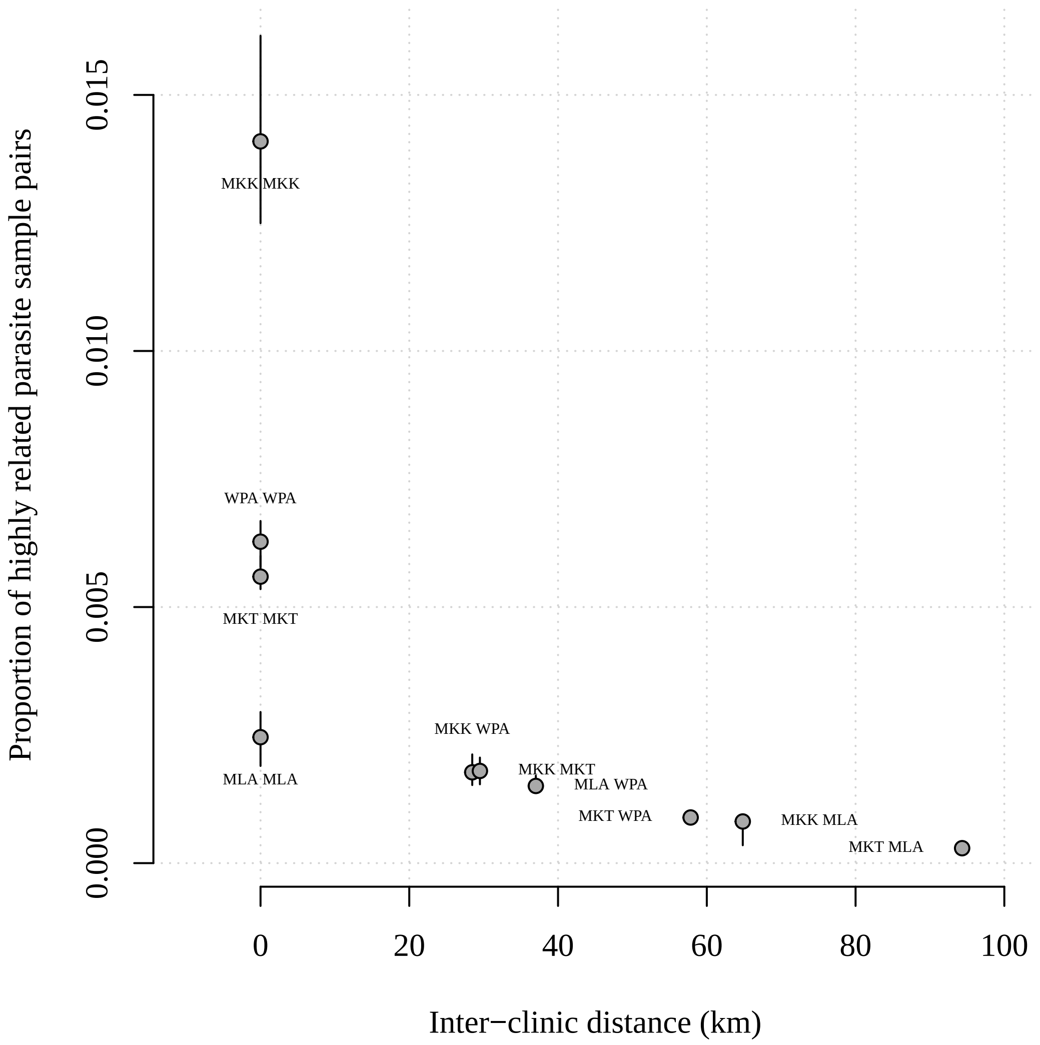

Figure O: Proportions of highly related 2001-2014 barcode parasite sample pairs with respect to inter-clinic distance. Annotations refer to site comparisons using abbreviated clinic names (MLA for Maela, 212 parasite samples; WPA for Wang Pha, 457 parasite samples; MKK for Mae Kon Ken, 116 parasite samples; and MKT for Mawker Thai, 388 parasite samples). All parasite samples were single-infection. For clinic pair A and B say, the proportion was based on $n_A \times n_B$ parasite samples, where n denotes the number of parasite samples per clinic. Error bars represent 95% confidence intervals based on bootstrapping over the clinic labels of highly related parasite sample pairs.

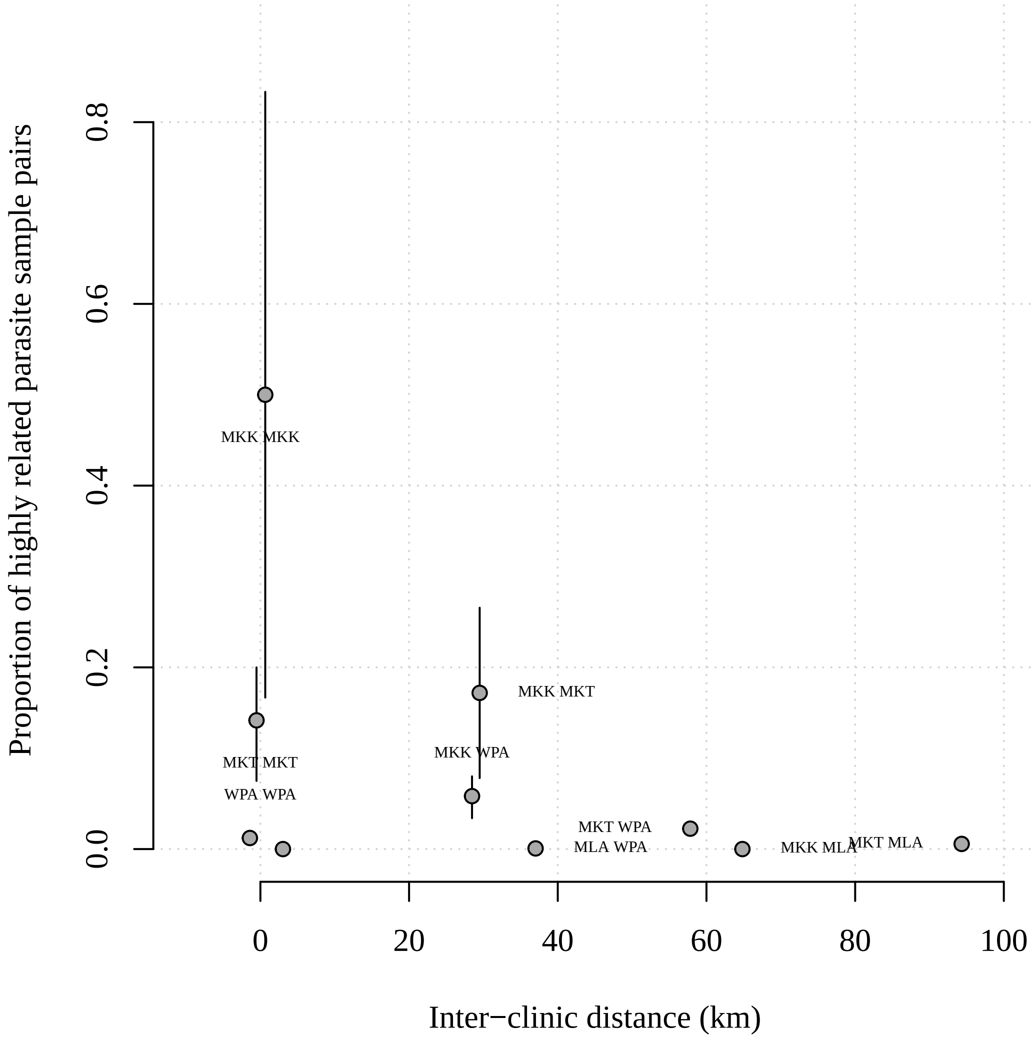

Figure P: Proportions of highly related WGS parasite sample pairs plotted with respect to inter-clinic distance. Annotations refer to site comparisons using abbreviated clinic names (MLA for Maela, 55 parasite samples; WPA for Wang Pha, 103 parasite samples; MKK for Mae Kon Ken, 4 parasite samples; and MKT for Mawker Thai, 16 parasite samples). All parasite samples were single-infection. For clinic pair A and B say, the proportion was based on $n_A \times n_B$ parasite samples, where n denotes the number of parasite samples per clinic. Error bars represent 95% confidence intervals based on bootstrapping over the clinic labels of highly related parasite sample pairs.

Table C: Trends in highly related barcode parasite sample pairs, excluding repeat barcodes within clinics. There were 18 repeat barcodes in Maela, 125 in Wang Pha, 28 in Mae Kon Ken, and 79 in Mawker Thai. Regression coefficient estimates unadjusted and adjusted refer to estimates before and after adjustment for temporal inputs, Season, ΔWeek, ΔWeek $\times$ Season and ΔWeeks $\times$ ΔDistance, respectively. P-values are two-tailed Monte Carlo estimates based on 1000 permutations of highly related parasite sample pair labels (equal to 1 if $\hat{\pi}_{\text{IBD}} > 0.5$ and 0 otherwise).

	Year 2001-2014		Year 2008		Year 2009		Year 2010	
	unadjusted	adjusted	unadjusted	adjusted	unadjusted	adjusted	unadjusted	adjusted
Intercept	-6.23e+00 (1.001)	-5.21e+00 (1.001)	-5.68e+00 (0.414)	-5.79e+00 (0.303)	-5.60e+00 (0.523)	-4.88e+00 (0.858)	-3.96e+00 (0.994)	-3.79e+00 (0.972)
Delta Distance (km)	-1.89e-02 (0.002)	-1.66e-02 (0.002)	-1.22e-02 (0.024)	-1.61e-02 (0.017)	-1.01e-02 (0.236)	-1.84e-02 (0.134)	-3.64e-02 (0.004)	-4.61e-02 (0.004)
Maela	-2.43e-01 (0.315)	1.91e-01 (0.448)	1.09e-01 (0.891)	3.60e-02 (0.973)	-1.40e+01 (0.309)	-1.40e+01 (0.305)	-7.85e-01 (0.514)	-7.26e-01 (0.579)
Wang Pha	2.91e-01 (0.093)	-1.63e-02 (0.946)	1.68e-01 (0.577)	1.68e-01 (0.581)	3.04e-01 (0.604)	1.83e-01 (0.749)	-1.29e+00 (0.047)	-1.42e+00 (0.026)
Mae Kon Ken	1.07e+00 (0.054)	7.10e-01 (0.162)	1.06e+00 (0.146)	1.07e+00 (0.147)	3.68e-01 (0.720)	3.74e-01 (0.733)	-1.46e+01 (0.123)	-1.47e+01 (0.119)
Mawker Thai	2.78e-01 (0.104)	7.24e-01 (0.002)	1.77e+00 (0.006)	1.79e+00 (0.004)	1.31e+00 (0.129)	1.28e+00 (0.131)	-7.25e-01 (0.440)	-7.50e-01 (0.421)
Season	NA (NA)	2.73e-01 (0.060)	NA (NA)	3.46e-01 (0.224)	NA (NA)	-5.92e-01 (0.295)	NA (NA)	8.55e-01 (0.175)
Delta weeks	NA (NA)	-1.88e-02 (0.002)	NA (NA)	-8.03e-04 (0.946)	NA (NA)	-4.12e-02 (0.152)	NA (NA)	-4.40e-02 (0.146)
Delta weeks x Season	NA (NA)	-2.65e-03 (0.002)	NA (NA)	-1.27e-03 (0.946)	NA (NA)	3.06e-02 (0.509)	NA (NA)	-4.94e-02 (0.355)
Delta weeks x Delta Distance	NA (NA)	9.46e-05 (0.002)	NA (NA)	2.39e-04 (0.358)	NA (NA)	6.01e-04 (0.307)	NA (NA)	8.89e-04 (0.119)

Sensitivity of spatial trends to the threshold used to classify highly related parasite sample pairs

Sensitivity to the threshold used to classify highly related sample pairs was assessed by translation of the specified threshold of 0.5. Specifically, for eight translated thresholds ranging from the specified threshold of 0.5 ± 0.3, we re-estimated temporally adjusted spatial trend estimates. Two-tailed Monte Carlo p-values were based on 100 permutations of binary outcomes (equal to one if $\hat{\pi}_{\text{IBD}} > 0.5$, and zero otherwise). Since significant negative trends were recovered for WGS data only when the increase in IBD in 2014 was accounted for (Table 3 main text), we used the temporally adjusted model with the year 2014 predictor to explore sensitivity using WGS data.

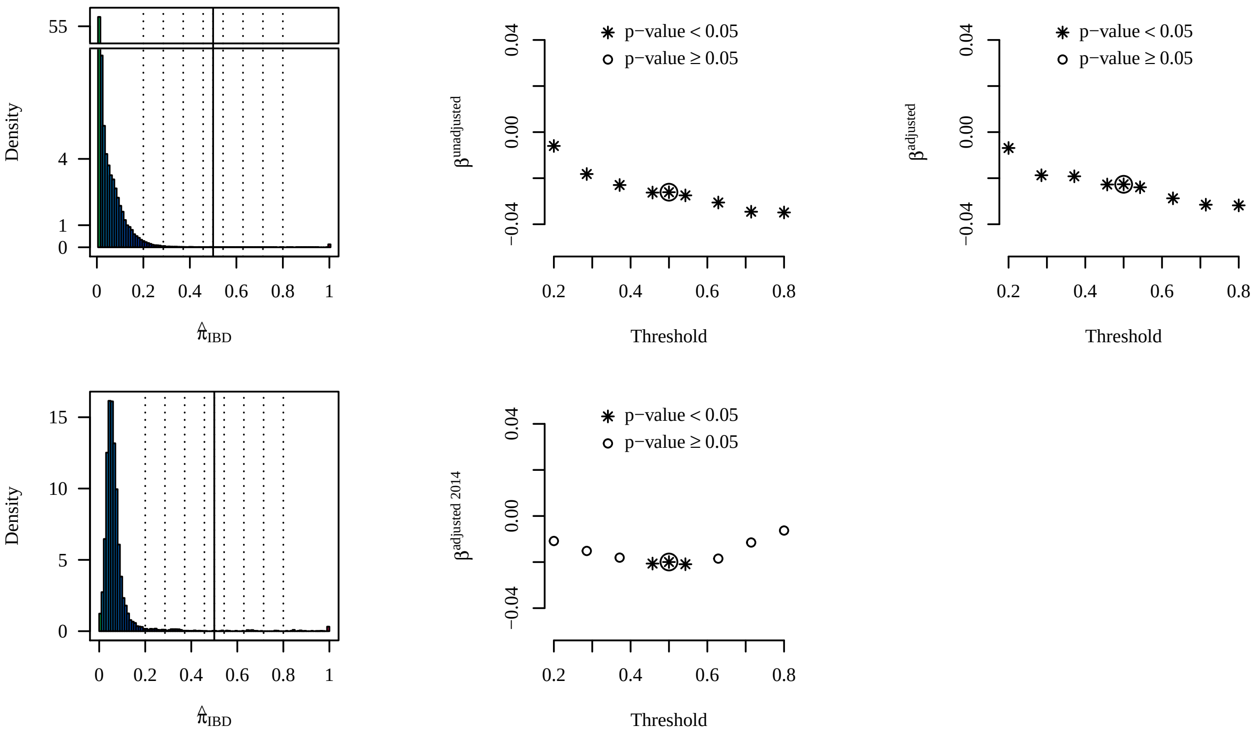

Figure Q: Threshold sensitivity of the spatial trend estimates based on barcode and WGS data. The plots on the left show the relatedness thresholds (solid vertical lines) and their translations (dashed vertical lines) in relation to the empirical distribution of $\hat{\pi}_{\text{IBD}}$ based on barcode data (top) and WGS data (bottom). The plots in the middle and on the right show ΔDistance trend estimates with respect to the relatedness thresholds and their translations. The trend estimates corresponding to the chosen threshold of 0.5 are circled.

Models based directly on $\hat{\pi}_{\text{IBD}}$

Thus far we have considered spatial trends in the $\mathbb{P}(\hat{\pi}_{\text{IBD}_i} > 0.5)$ (see Tables 2 and 3 of the main manuscript for regression coefficient estimates) because empirical density plots suggested $\hat{\pi}_{\text{IBD}}$ were loosely distributed according to different classes, with perceived recent migrants having high $\hat{\pi}_{\text{IBD}}$ (Fig A to S2.J). Alternatively, we could regress $\hat{\pi}_{\text{IBD}}$ directly onto distance by letting $z_i = \hat{\pi}_{\text{IBD}_i}$. Under chosen models 2 and 8 (and 11 for WGS data) with $z_i = \hat{\pi}_{\text{IBD}_i}$, the spatial trend estimates were mostly negative, though very small, and those that are significant in Tables 2 and 3 of the main manuscript are similarly significant here (Table D).

Table D: Regression coefficients estimated under temporally unadjusted and adjusted models (models 2 and 8, respectively) fit to 2001-2010 barcode and 2001-2014 WGS data. The temporally adjusted year model (model 11) was also fit to 2001-2014 WGS data. P-values are two-tailed Monte Carlo estimates based on 1000 permutations of the $\hat{\pi}_{\text{IBD}}$ and are given in parentheses.

	Barcode unadjusted	Barcode adjusted	WGS unadjusted	WGS adjusted	WGS adjusted year
Intercept	0.03245 (0.523)	0.03809 (0.002)	0.07281 (0.562)	0.07872 (0.055)	0.08771 (0.002)
Delta Distance (km)	-0.00003 (0.002)	-0.00009 (0.002)	-0.00007 (0.160)	0.00001 (0.941)	-0.00047 (0.002)
Maela	-0.00071 (0.173)	0.00127 (0.019)	-0.01127 (0.002)	-0.00575 (0.123)	-0.01479 (0.002)
Wang Pha	0.00599 (0.002)	0.00247 (0.002)	0.00679 (0.016)	-0.00199 (0.538)	-0.00910 (0.004)
Mae Kon Ken	0.01162 (0.002)	0.00767 (0.002)	0.42419 (0.002)	0.38026 (0.002)	0.30680 (0.002)
Mawker Thai	0.00307 (0.002)	0.00470 (0.002)	0.12590 (0.002)	0.08179 (0.002)	0.00837 (0.400)
Season	NA	0.00073 (0.004)	NA	0.03907 (0.002)	0.02639 (0.002)
Delta weeks	NA	-0.00004 (0.002)	NA	-0.00005 (0.002)	-0.00006 (0.002)
Delta weeks x Season	NA	-0.00000 (0.030)	NA	-0.00011 (0.002)	-0.00007 (0.002)
Delta weeks x Delta Distance	NA	0.00000 (0.002)	NA	-0.00000 (0.892)	0.00000 (0.002)
Year	NA	NA	NA	NA	0.07690 (0.002)

References

Akaike, Hirotogu. 1998. "Information Theory and an Extension of the Maximum Likelihood Principle." In *Selected Papers of Hirotugu Akaike*, 199–213. Springer New York.

Gelman, Andrew, and Jennifer Hill. 2007. *Data analysis using regression and multilevel/hierarchical models*. New York, NY.
