# Supplementary material for: Quantifying connectivity between local Plasmodium falciparum malaria parasite populations using identity by descent
Source: PLoS Genet. 2017 Oct 27;13(10):e1007065. doi: 10.1371/journal.pgen.1007065 (PMC5678785; doi:10.1371/journal.pgen.1007065)
Supplement: S3 Text — (PDF) [file pgen.1007065.s007.pdf]

# S3 Text

Throughout this document, pairs of clinics are denoted by their abbreviated names (MLA for Maela, WPA for Wang Pha, MKK for Mae Kon Ken and MKT for Mawker Thai).

We estimated clinic-averaged co-ancestries from WGS data assuming SNPs were both linked and unlinked. Linked analyses require a recombination file specifying distances between SNPs. We used a uniform recombination rate equal to $7.4 \times 10^{-7}$ Morgans per base pair (Miles et al. 2016), the same as that specified in hmmIBD (Schaffner et al., n.d.). ChromoPainter essentially treats each unlinked SNP as an independent segment (Lawson et al. 2012), increasing the number sharable segments when SNPs are considered unlinked (compare the ranges of the legends in Fig A). Clinic-averaged co-ancestry estimates based on linked SNPs were more anisotropic than their unlinked counterparts (top versus bottom plot, Fig A). Anisotropy had little impact upon downstream analyses, however, which were otherwise very similar for linked and unlinked estimates (Fig B).

ChromoPainter was designed for dense WGS data (Lawson et al. 2012), with typically 100,000 or more SNPs across the human genome (personal communication). Nevertheless, we tried to estimate clinic-averaged co-ancestries using barcode data. Unsurprisingly, the markers were too far separated to support a linked analysis. We therefore present results from an unlinked analysis of barcode data only.

ChromoPainter does not support missing data. We therefore imputed missing SNPs (both barcode and WGS) by sampling from a Bernoulli distribution with probability equal to the sample allele frequency at the locus corresponding to the missing SNP.

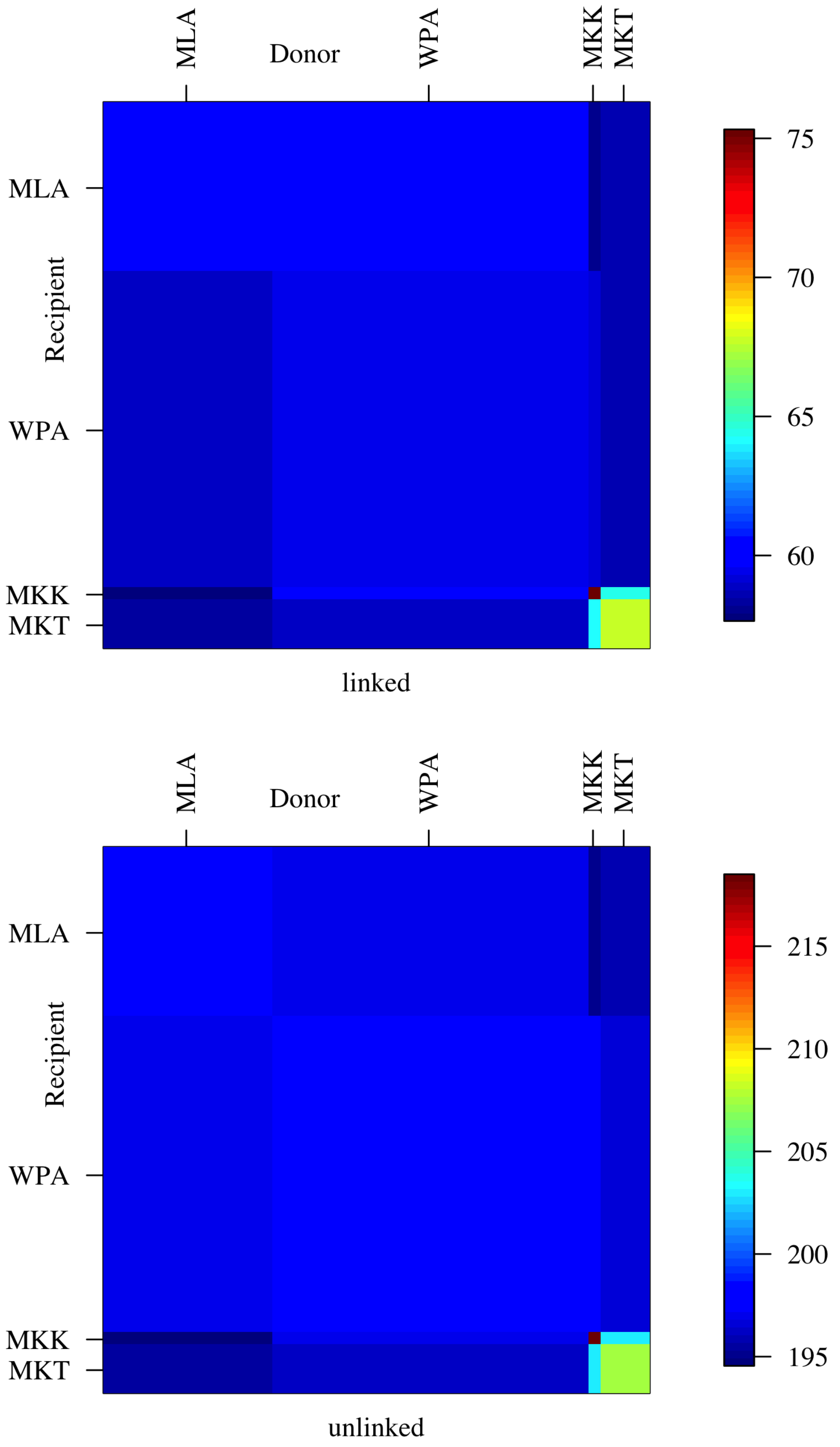


Figure A: Clinic-averaged co-ancestry estimates based on WGS data.

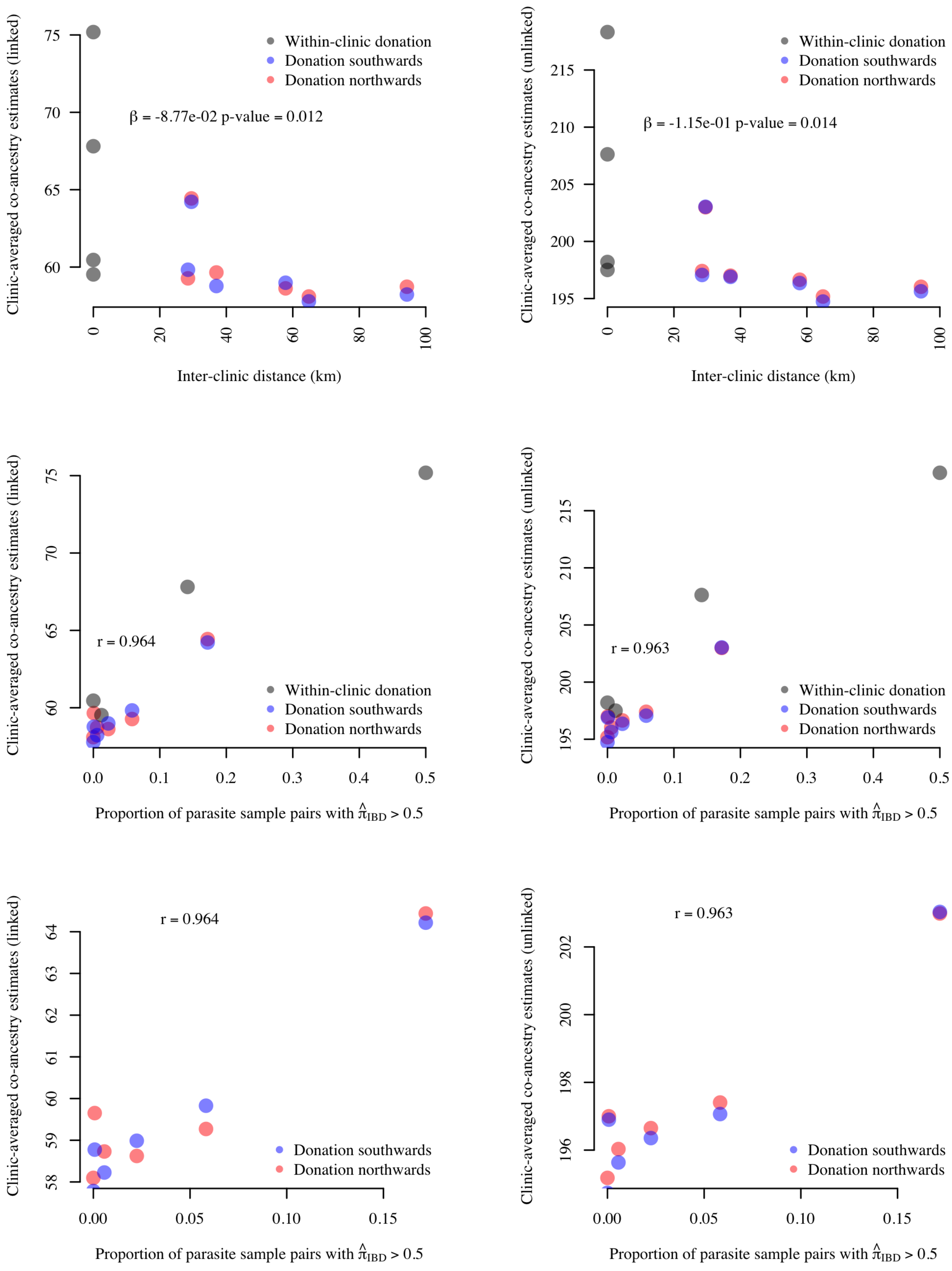


Figure B: Trends and correlates of clinic-averaged co-ancestry estimates based on WGS data. P-values are two-tailed Monte Carlo estimates based on 1000 permutations of the clinic-averaged co-ancestries.

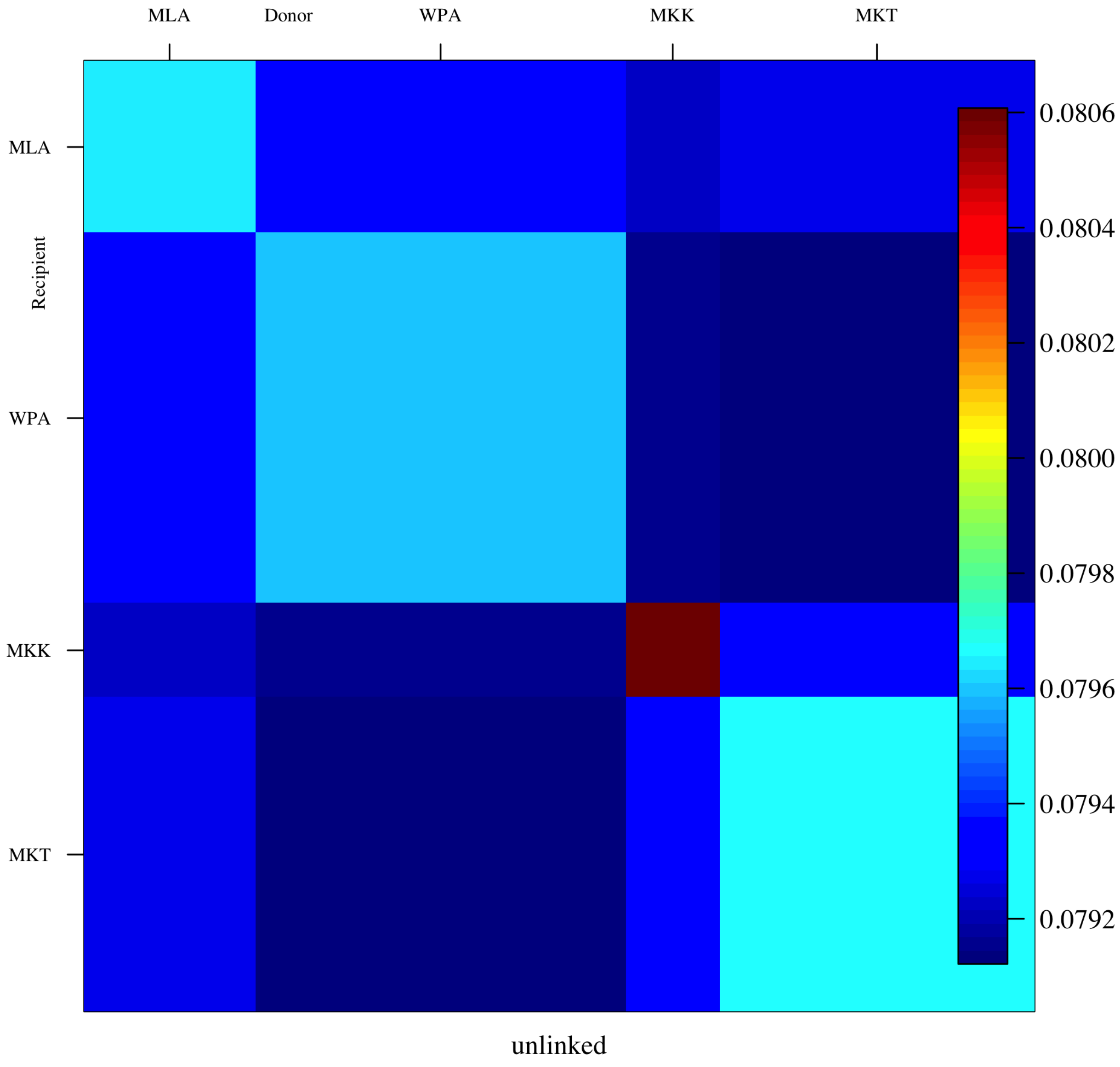


Figure C: Clinic-averaged co-ancestry estimates based on barcode data.

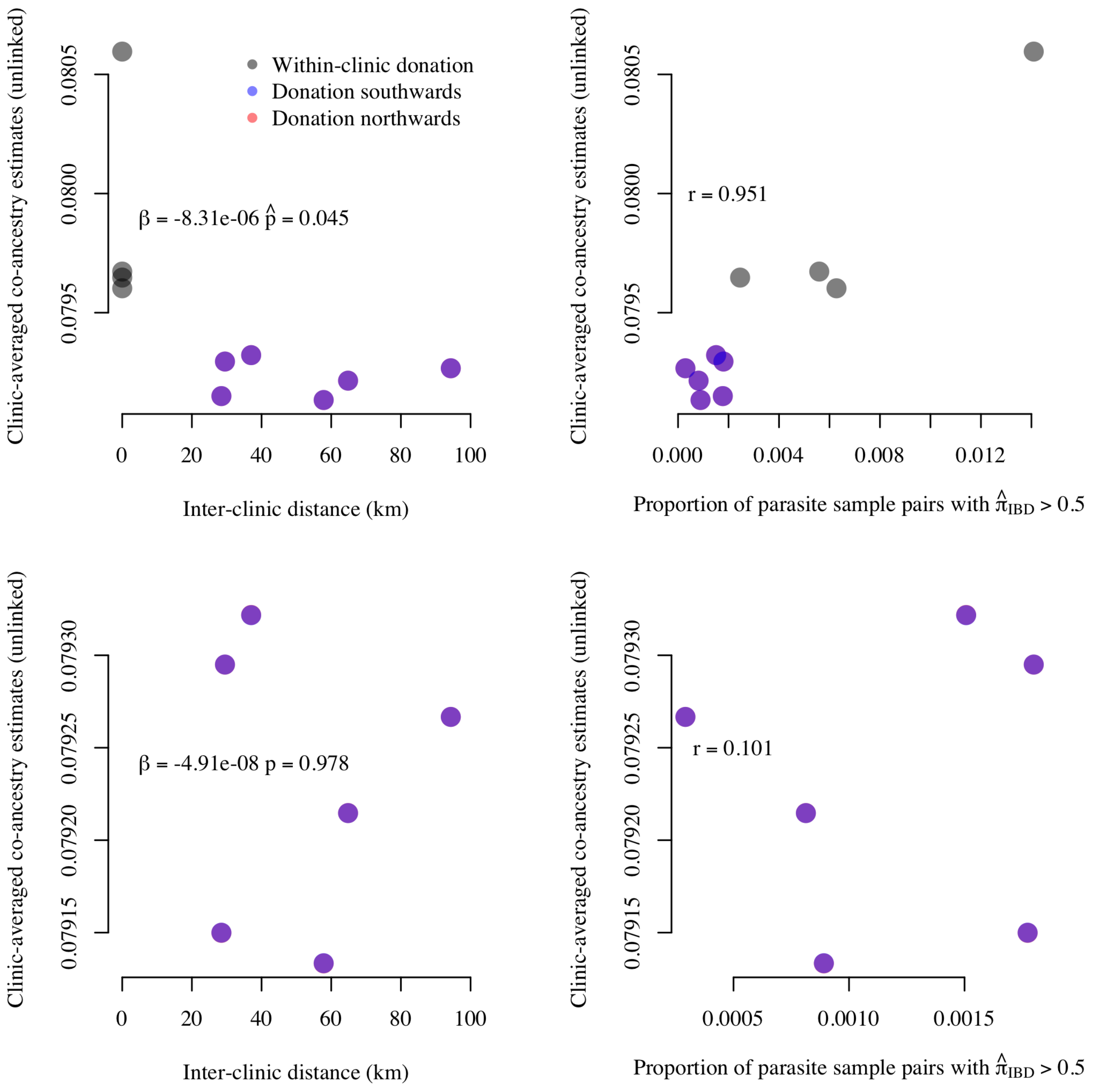


Figure D: Trends and correlates of clinic-averaged co-ancestry estimates based on barcode data. Trend estimates and p-values were based on within and northwards co-ancestries only, since north and southward estimates are practically identical. P-values are two-tailed: $\hat{p}$ denotes a Monte Carlo estimate based on 1000 permutations of the clinic-averaged co-ancestries, $p$ denotes an exact p-value based on $6! = 720$ possible permutations.

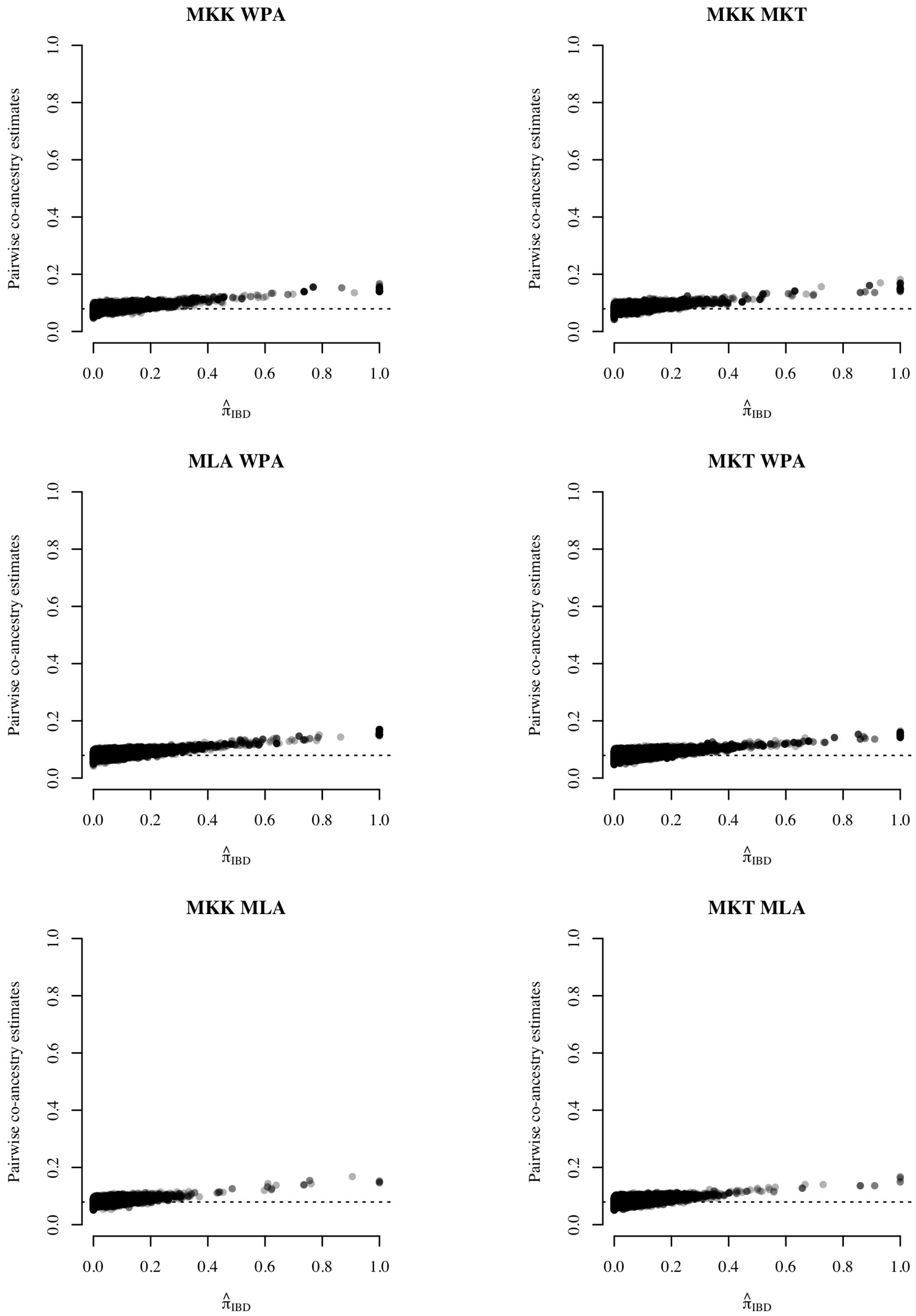


Figure E: Pairwise co-ancestry estimates and $\hat{\pi}_{\mathrm{IBD}}$ based on barcode data plotted on zero-one axes.

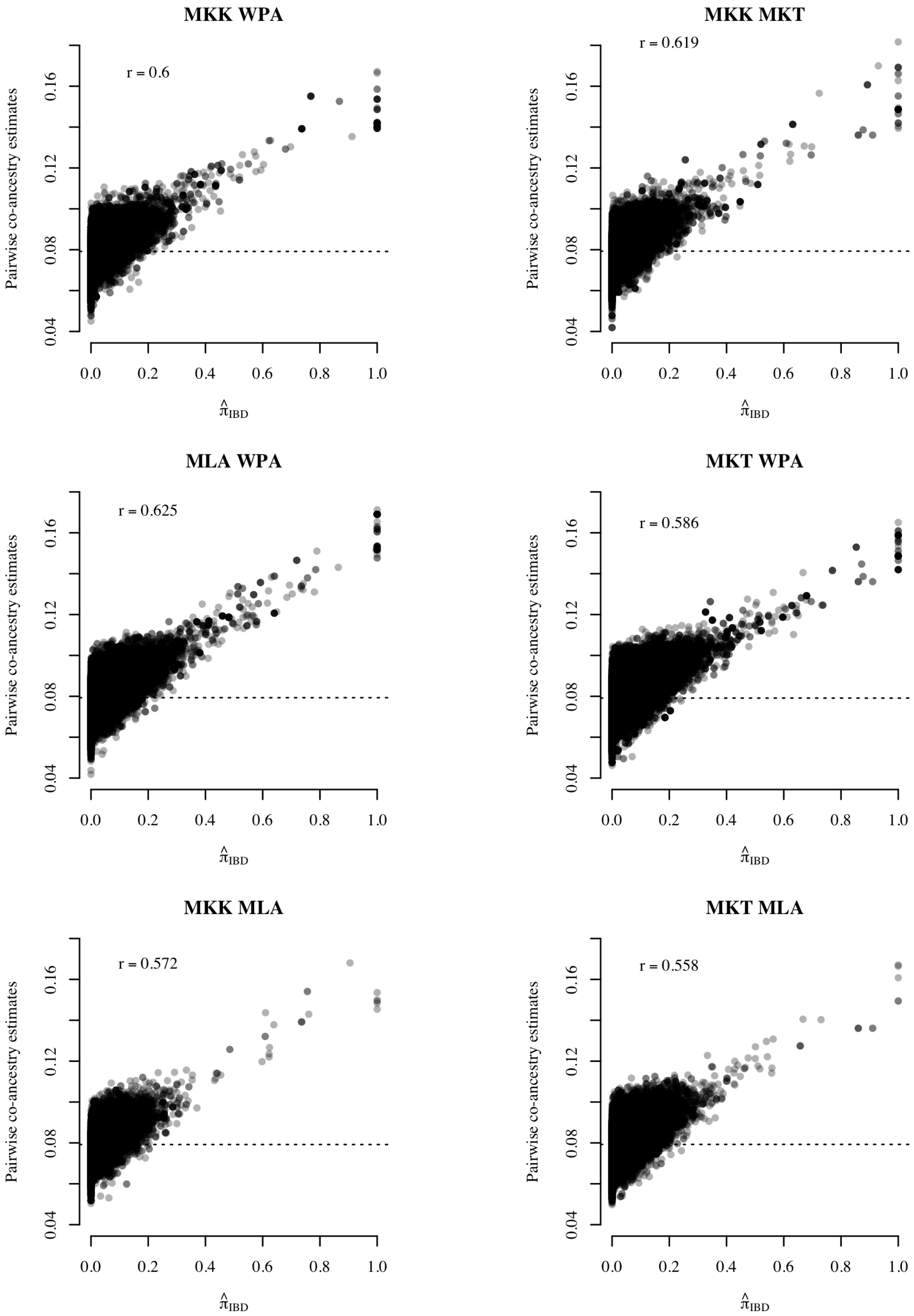


Figure F: Pairwise co-ancestry estimates and $\hat{\pi}_{\text{IBD}}$ based on barcode data.

# References

Lawson, Daniel John, Garrett Hellenthal, Simon Myers, and Daniel Falush. 2012. "Inference of Population Structure using Dense Haplotype Data." *PLoS Genetics* 8 (1): e1002453.

Miles, Alistair, Zamin Iqbal, Paul Vauterin, Richard Pearson, Susana Campino, Michel Theron, Kelda Gould, et al. 2016. "Indels, structural variation and recombination drive genomic diversity in Plasmodium falciparum." *Genome Research* 26 (9): 1288–99.

Schaffner, Stephen F, Aimee R Taylor, Wesley Wong, F Dyann, and Daniel E Neafsey. n.d. "hmmIBD: software to infer pairwise identity by descent between haploid genotypes; 2017. Preprint. Available from: bioRxiv doi: 10.1101/188078. Cited 4 October 2017."
