# Supplementary material for: Quantifying connectivity between local Plasmodium falciparum malaria parasite populations using identity by descent
Source: PLoS Genet. 2017 Oct 27;13(10):e1007065. doi: 10.1371/journal.pgen.1007065 (PMC5678785; doi:10.1371/journal.pgen.1007065)
Supplement: S4 Text — (PDF) [file pgen.1007065.s008.pdf]

S4 Text

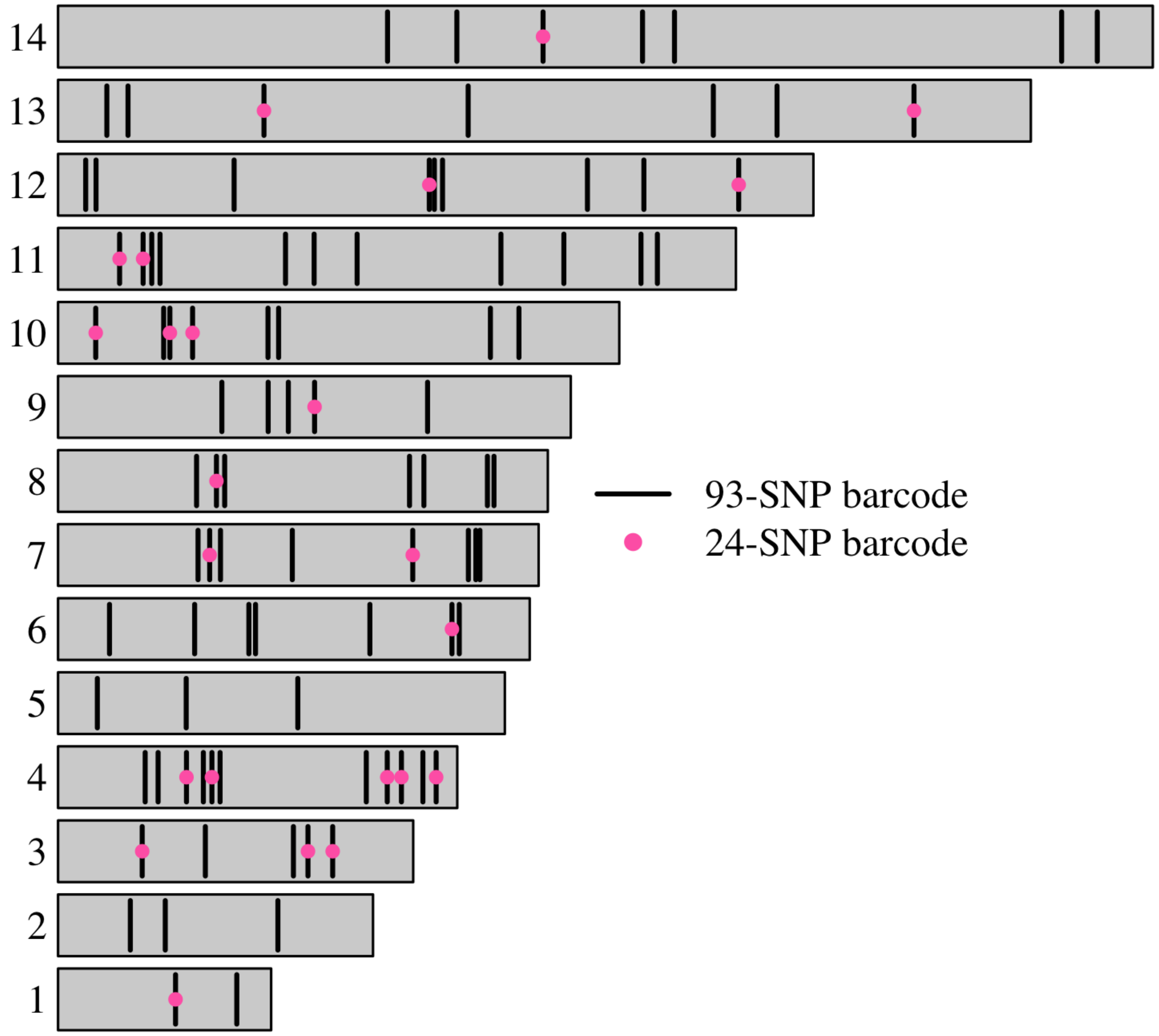

Figure A: 93 and 24-SNP barcode positions over 14 extit{P. falciparum} chromosomes.

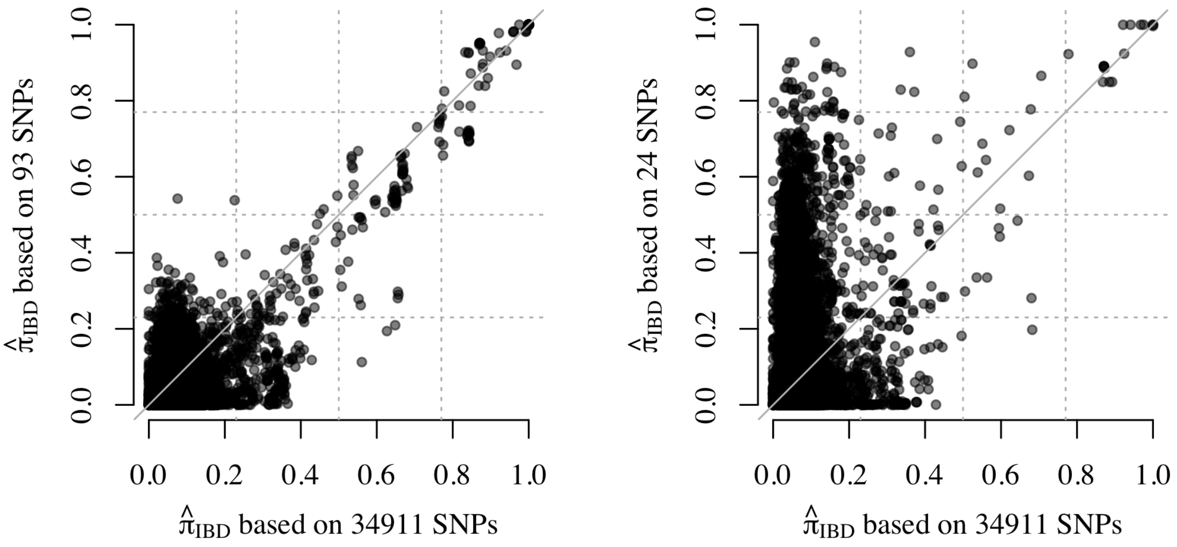

Figure B: Comparison between $\hat{\pi}_{\text{IBD}}$ based on the whole genome and 93 and 24 SNP subsets generated using 2001-2014 WGS data.

Dependence between barcode SNPs under hmmIBD

To capture dependence between SNPs as a function of inter-SNP distance d_t (in base pairs), the hidden Markov model underpinning hmmIBD (see Appendix S1 of Schaffner et al., n.d. for full details) includes a matrix whose elements are probabilities of switching between IBD and not IBD states at successive SNPs, denoted here by SNP_{t-1} and SNP_t,

$$\boldsymbol{A}(t) = \begin{bmatrix} \mathbb{P}\big(\text{SNP}_t = \text{IBD} \mid \text{SNP}_{t-1} = \text{IBD}\big) & \mathbb{P}\big(\text{SNP}_t = \text{not IBD} \mid \text{SNP}_{t-1} = \text{IBD}\big) \\ \mathbb{P}\big(\text{SNP}_t = \text{IBD} \mid \text{SNP}_{t-1} = \text{not IBD}\big) & \mathbb{P}\big(\text{SNP}_t = \text{not IBD} \mid \text{SNP}_{t-1} = \text{not IBD}\big) \end{bmatrix},$$

$$= \begin{bmatrix} 1 - \pi_2(1 - e^{-k\rho d_t}) & \pi_2(1 - e^{-k\rho d_t}) \\ \pi_1(1 - e^{-k\rho d_t}) & 1 - \pi_1(1 - e^{-k\rho d_t}) \end{bmatrix},$$

where π_1 and $\pi_2 = 1 - \pi_1$ are the expected fraction IBD and not IBD, respectively (π_1 is inferred under the model, and is the output of interest, denoted $\hat{\pi}_{\text{IBD}}$, in the current study); ρ is the recombination rate; and k is the number of generations since the most recent common ancestor. Both ρ and k are considered fixed across the genome. Akin to π_1, k is inferred under the model. When distances are large the exponential term tends to zero. That is, $\lim_{d_t \to \infty} e^{-k\rho d_t} = 0$, such that

$$\lim_{d_t \to \infty} \boldsymbol{A}(t) = \begin{bmatrix} 1 - \pi_2 & \pi_2 \\ \pi_1 & 1 - \pi_1 \end{bmatrix},$$

$$= \begin{bmatrix} \mathbb{P}\big(\text{SNP}_t = \text{IBD}\big) & \mathbb{P}\big(\text{SNP}_t = \text{not IBD}\big) \\ \mathbb{P}\big(\text{SNP}_t = \text{IBD}\big) & \mathbb{P}\big(\text{SNP}_t = \text{not IBD}\big) \end{bmatrix}.$$

In other words, when distances are sufficiently large that $e^{-k\rho d_t} \approx 0$, SNPs are effectively independent of one another. Fig C shows that for $\rho = 7.4 \times 10^{-7}$ base pairs per Morgan (Miles et al. 2016), and small numbers of generations, $k < 50$, $e^{-k\rho d_t} \neq 0$ for most distances between barcode SNPs. That is to say, barcode SNPs are dependent under hmmIBD providing k is small because ρ is low.

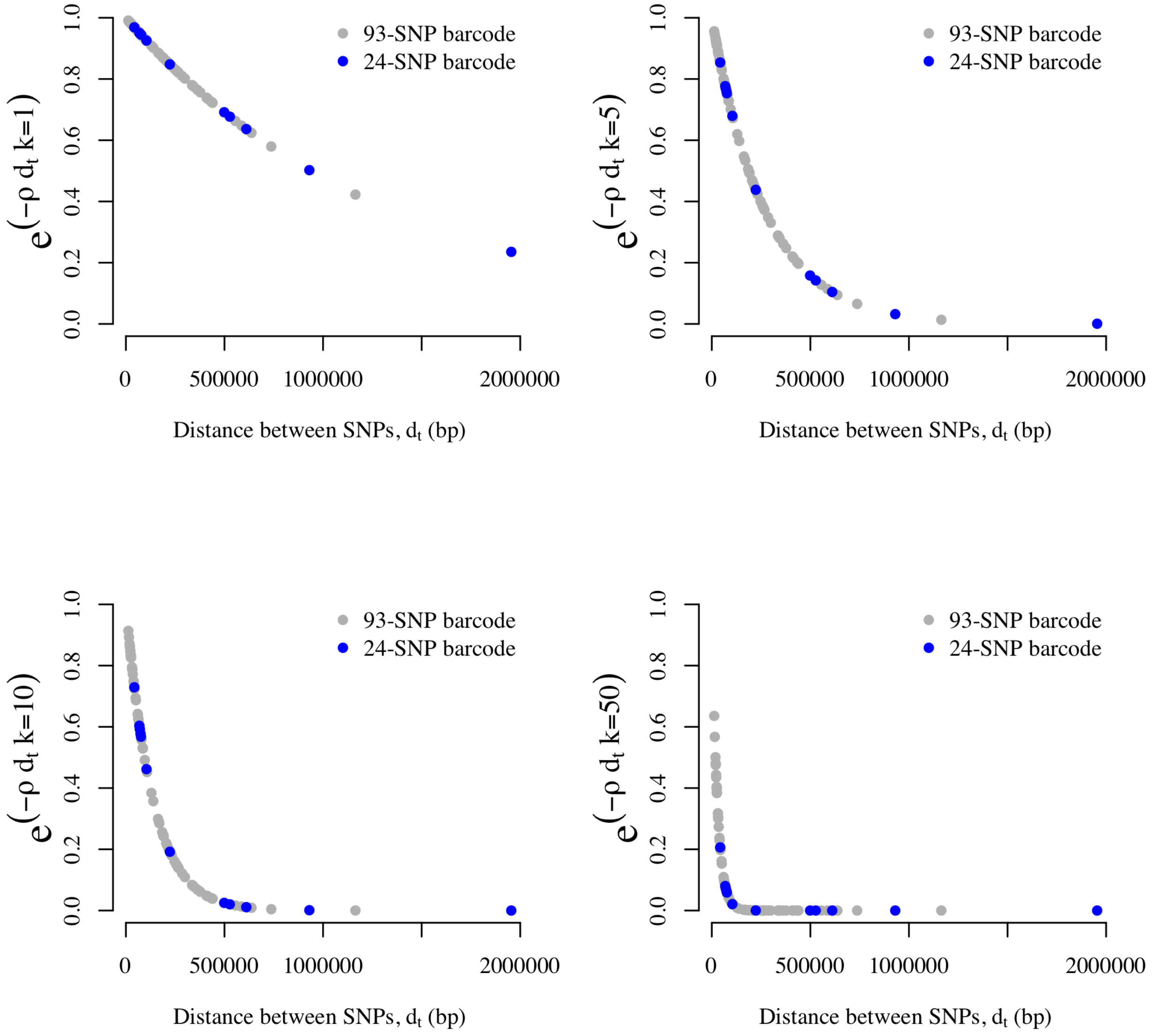

Figure C: The effect of inter-93 and 24 barcode SNP distances on $\exp(-\rho d_t k)$ given different numbers of generations, k.

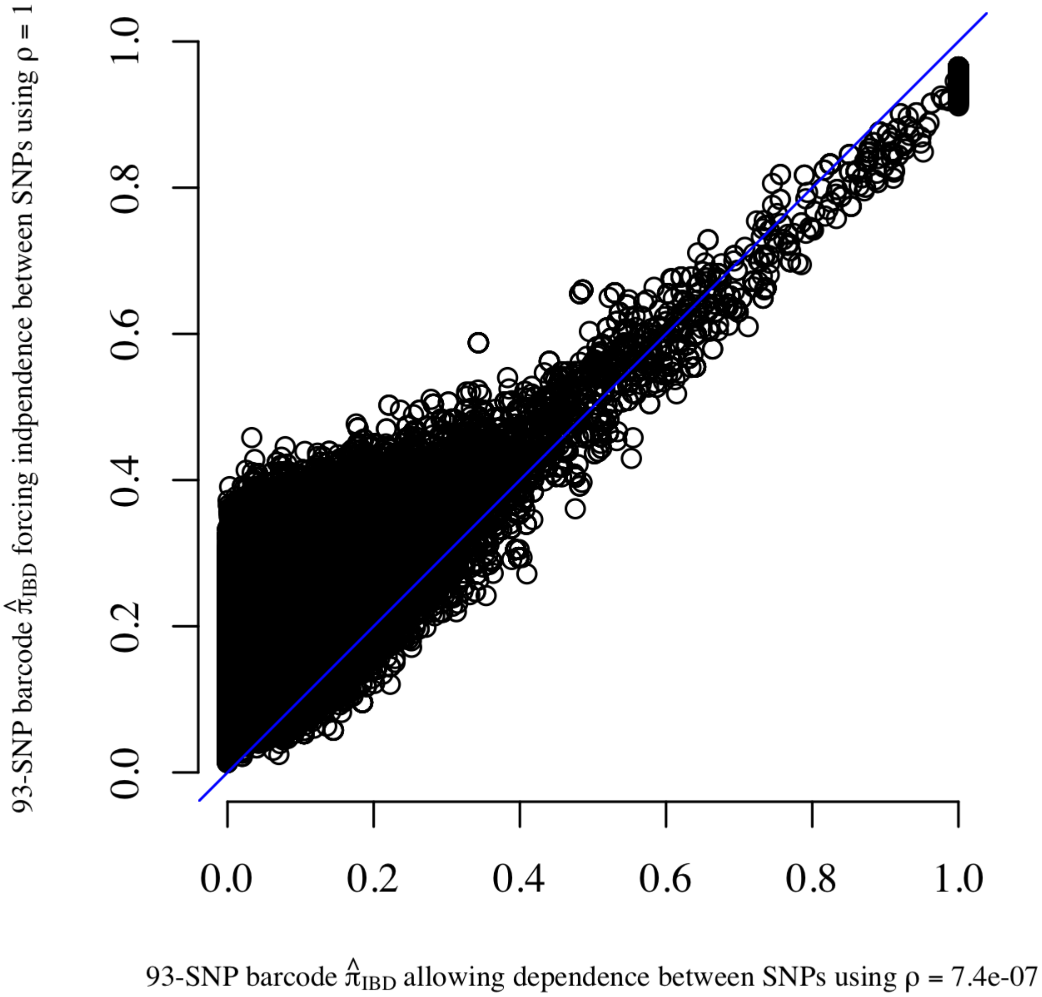

Figure D: IBD proportion estimates generated under hmmIBD both allowing dependence and forcing independence between SNPs by varying ρ.

References

Miles, Alistair, Zamin Iqbal, Paul Vauterin, Richard Pearson, Susana Campino, Michel Theron, Kelda Gould, et al. 2016. “Indels, structural variation and recombination drive genomic diversity in Plasmodium falciparum.” *Genome Research* 26 (9): 1288–99.

Schaffner, Stephen F, Aimee R Taylor, Wesley Wong, F Dyann, and Daniel E Neafsey. n.d. “hmmIBD: software to infer pairwise identity by descent between haploid genotypes; 2017. Preprint. Available from: bioRxiv doi: 10.1101/188078. Cited 4 October 2017.”
